# An estrogen receptor α-derived peptide improves glucose homeostasis during obesity

**DOI:** 10.1038/s41467-024-47687-6

**Published:** 2024-04-22

**Authors:** Wanbao Yang, Wen Jiang, Wang Liao, Hui Yan, Weiqi Ai, Quan Pan, Wesley A. Brashear, Yong Xu, Ling He, Shaodong Guo

**Affiliations:** 1 Department of Nutrition, College of Agriculture and Life Sciences, Texas A&M University, College Station, TX 77843 USA; 2 High Performance Research Computing, Texas A&M University, College Station, TX USA; 3 USDA/ARS Children’s Nutrition Research Center, Department of Pediatrics, Baylor College of Medicine, Houston, TX 77030 USA; 4 Departments of Pediatrics and Pharmacology, Johns Hopkins University School of Medicine, Baltimore, MD USA

**Keywords:** Type 2 diabetes, Metabolic syndrome, Obesity

## Abstract

Estrogen receptor α (ERα) plays a crucial role in regulating glucose and energy homeostasis during type 2 diabetes mellitus (T2DM). However, the underlying mechanisms remain incompletely understood. Here we find a ligand-independent effect of ERα on the regulation of glucose homeostasis. Deficiency of ERα in the liver impairs glucose homeostasis in male, female, and ovariectomized (OVX) female mice. Mechanistic studies reveal that ERα promotes hepatic insulin sensitivity by suppressing ubiquitination-induced IRS1 degradation. The ERα 1-280 domain mediates the ligand-independent effect of ERα on insulin sensitivity. Furthermore, we identify a peptide based on ERα 1-280 domain and find that ERα-derived peptide increases IRS1 stability and enhances insulin sensitivity. Importantly, administration of ERα-derived peptide into obese mice significantly improves glucose homeostasis and serum lipid profiles. These findings pave the way for the therapeutic intervention of T2DM by targeting the ligand-independent effect of ERα and indicate that ERα-derived peptide is a potential insulin sensitizer for the treatment of T2DM.

## Abstract (web summary)

Type 2 diabetes mellitus is an increasing global health issue, which is caused by systemic insulin resistance. Here, the authors show a ligand-independent effect of hepatic ERα in regulating insulin sensitivity and identify an ERα-derived peptide that functions as an insulin sensitizer.

## Introduction

Approximately 415 million people worldwide have diabetes with an estimated 193 million people having undiagnosed diabetes^1^. More than 90% of diabetic patients have type 2 diabetes mellitus (T2DM) that is characterized by insulin resistance and relative insulin deficiency. Insulin resistance is known as an impaired insulin action in target organs, which eventually leads to a relative insulin deficiency. The underlying mechanisms of insulin resistance are complicated, including direct consequence of toxic metabolic by-products accumulation, dysregulation of peptide hormones and inflammatory molecules, as well as activation of intracellular stress response pathways^2^. The antidiabetic medications have been developed by targeting hepatic glucose production (HGP), insulin secretion, and insulin sensitivity during the past several decades^3^. Considering the complexity of diabetes and side-effects of current antidiabetic medicines, it is important to develop the novel therapeutic target of T2DM.

Estradiol-17β (E_2_) plays a critical role in the regulation of energy balance and glucose homeostasis, which may explain the fact that diabetes is more prevalent in young men than young women^4–7^. The function of E_2_ is mainly mediated through estrogen receptors (ER). Ablation of ERα, but not ERβ, leads to metabolic dysregulation in mice, including increased body weight, enhanced adiposity, and impaired glucose homeostasis^8,9^. Although the role of E_2_-ERα signaling pathway in energy balance and glucose homeostasis is well-documented, its underlying mechanisms are less known. E_2_-ERα signaling pathway functions through a genomic mechanism, where E_2_ activates ERα to bind to the estrogen response element (ERE) motif and regulate the transcription of target genes. ERα activated by E_2_ also triggers downstream signaling pathway through a non-genomic mechanism, including interaction with cell membrane receptors and activation of protein kinases^10^. A previous study has shown that the rescue of ERα non-genomic function normalizes the dysregulation of energy balance and glucose homeostasis in obese ERα knockout female mice; this suggests that the non-genomic action of E_2_-ERα signaling plays a pivotal role in the regulation of glucose and energy homeostasis^11^. However, the underlying mechanism of E_2_-ERα non-genomic action is not fully understood.

Our previous study showed that E_2_ suppressed HGP and increased insulin sensitivity through the activation of AKT-FOXO1 signaling pathway^12^. Other study revealed that ERα interacted with and activated p85α regulatory subunit of phosphatidylinositol-3-OH kinase (PI3K) in response to E_2_ in endothelial cells^13^. Thus, E_2_-ERα-PI3K-AKT-FOXO1 signaling pathway partially mediates the non-genomic effect of E_2_-ERα on energy balance and glucose homeostasis. In addition, we found that overexpression of ERα significantly promoted insulin sensitivity in absence of E_2_^12^; this suggests that ERα enhances insulin sensitivity in a ligand-independent manner. Insulin receptor substrate (IRS) 1 and 2 are the key targets of insulin receptor to control glucose homeostasis in response to insulin^14^. Hepatic ablation of IRS1 and IRS2 leads to insulin resistance and hyperglycemia in mice^15^. In breast cancer cells, ERα interacts with IRS1 and IRS2 independent of E_2_, thereby protecting against ubiquitination-induced degradation^16^. Therefore, we hypothesized that ERα binds to the IRS proteins in a ligand-independent manner, thereby regulating insulin sensitivity and glucose homeostasis. In this study, we found that hepatic ERα deletion resulted in glucose intolerance in male, female, and ovariectomized (OVX) female mice. Mechanistically, ERα increased IRS1 protein stability through inhibiting the phosphorylation of IRS1 at S302 and ubiquitin-induced degradation. Furthermore, we found that ERα 1-280 domain mediated the ligand-independent effect of ERα on insulin sensitivity and glucose homeostasis. Finally, we designed a peptide (AF1 peptide) based on amino acid sequence of ERα 1-280 domain and demonstrated that AF1 peptide was a potential insulin sensitizer to increase insulin sensitivity and improve glucose homeostasis in obese mice.

## Results

### Deletion of hepatic ERα impairs glucose tolerance and insulin sensitivity in both male and female mice

**Fig. 1:**
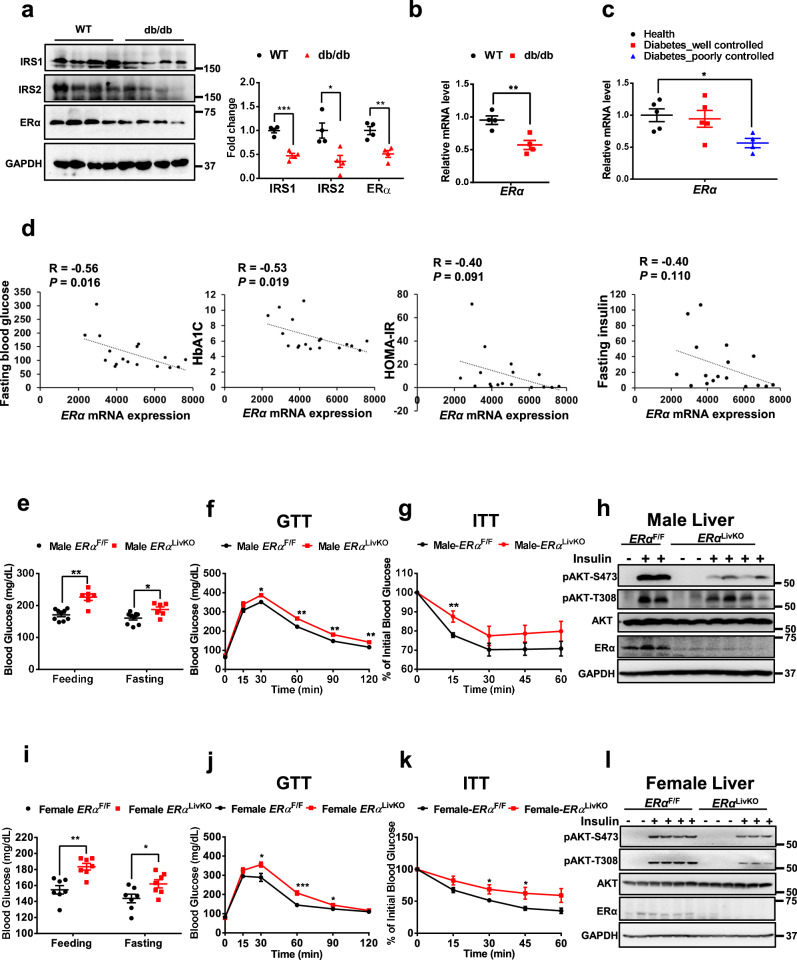
Hepatic ERα knockout impairs glucose tolerance and insulin sensitivity in both male and female mice. **a** IRS1, IRS2, and ERα protein levels in the livers of random-feeding WT and db/db mice, *n* = 4 mice/group; for IRS1, *P* = 0.0002; for IRS2, *P* = 0.0183; for ERα, *P* = 0.0045. **b** mRNA expression levels of *ERα* in the livers of random feeding WT and db/db mice, *n* = 4 mice/group; *P* = 0.0078. **c** mRNA expression levels of *ERα* in the livers of humans with diabetes, *n* = 4 (Diabetes poorly controlled) and 5 (Health and Diabetes well controlled); *P* = 0.0444. **d** Person correlation coefficient between the Fasting blood glucose/HbA1C/HOMA-IR/Fasting insulin and the mRNA expression levels of *ERα* in the livers of humans, *n* = 17 (Fasting blood glucose, Fasting insulin, and HOMA-IR) and 18 (HbA1C). **e** Random feeding and 5 h fasting blood glucose in *ERα*^F/F^ and *ERα*^LivKO^ male mice, *n* = 6 (*ERα*^LivKO^) and 9 (*ERα*^F/F^) mice/group; feeding blood glucose, *P* = 0.0002; 5 h fasting blood glucose, *P* = 0.0194. **f** Glucose tolerance tests in *ERα*^F/F^ and *ERα*^LivKO^ male mice, *n* = 8 (*ERα*^LivKO^) and 10 (*ERα*^F/F^) mice/group; 30 min, *P* = 0.0124; 60 min, *P* = 0.0013; 90 min, *P* = 0.0032; 120 min, *P* = 0.0070. **g** Insulin tolerance tests in *ERα*^F/F^ and *ERα*^LivKO^ male mice, *n* = 6 (*ERα*^LivKO^) and 7 (*ERα*^F/F^) mice/group; 15 min, *P* = 0.0078. **h** Insulin signaling was detected in livers from *ERα*^F/F^ and *ERα*^LivKO^ male mice injected with 2 U insulin for 5 min. The experiments were repeated independently three times. Representative blots were shown. **i** Random feeding and 5 h fasting blood glucose in *ERα*^F/F^ and *ERα*^LivKO^ female mice, *n* = 7 mice/group; feeding blood glucose, *P* = 0.0011; 5 h fasting blood glucose, *P* = 0.0299. **j** Glucose tolerance tests in *ERα*^F/F^ and *ERα*^LivKO^ female mice, *n* = 7 mice/group; 30 min, *P* = 0.0185; 60 min, *P* = 0.0005; 90 min, *P* = 0.0444. **k** Insulin tolerance tests in *ERα*^F/F^ and *ERα*^LivKO^ female mice, *n* = 7 mice/group; 30 min, *P* = 0.0437; 45 min, *P* = 0.0309. **l** Insulin signaling was detected in livers from *ERα*^F/F^ and *ERα*^LivKO^ female mice injected with 2 U insulin for 5 min. The experiments were repeated independently three times. Representative blots were shown. Data are presented as mean ± SEM. *P < 0.05, **P < 0.01, ***P < 0.001, unpaired Two-tailed Student’s t test (**a**, **b**, **e**–**g**, **i**–**k**) or One-way ANOVA with Tukey’s multiple comparisons test (**c**). Source data are provided as a Source Data file.

We first detected the expression levels of IRS1, IRS2, and ERα in the liver of obese db/db male mice. We found that compared to wild-type (WT) mice, IRS1, IRS2, and ERα protein levels were significantly decreased by 53%, 64%, and 49%, respectively, in the liver of db/db male mice (Fig. 1a). In addition, the mRNA expression levels of *ERα* were significantly decreased in the liver of diabetic mice (Fig. 1b). We further analyzed the mRNA expression levels of hepatic *ERα* in diabetic patients^17^. Interestingly, hepatic *ERα* mRNA expression levels were significantly decreased in humans with poorly controlled diabetes but not well controlled diabetes (Fig. 1c). Compared to well controlled diabetic individuals, poorly controlled diabetic individuals showed a higher fasting insulin level (Fig. S1a). High dose of insulin treatment (200 nM) significantly decreased mRNA expression levels of *ERα* in primary hepatocytes (Fig. S1b), suggesting that decreased hepatic *ERα* mRNA expression in diabetic patients may be caused by hyperinsulinemia. We then analyzed the gene profiles in the livers of humans and found that hepatic *ERα* expression levels were negatively associated with fasting blood glucose, HbA1C, HOMA-IR, and fasting insulin (Fig. 1d). These results indicate that hepatic ERα plays an important role in regulating pathogenesis of T2DM.

To further detect the effect of hepatic ERα on glucose homeostasis, we generated liver-specific ERα knockout (*ERα*^LivKO^) mice. Glucose tolerance and insulin sensitivity were analyzed in both male and female *ERα*^LivKO^ mice. Compared to *ERα*-floxed (*ERα*^F/F^) male mice, *ERα*^LivKO^ male mice showed a 32% increase in random feeding blood glucose (*ERα*^F/F^: 170.9 ± 5.7 vs. *ERα*^LivKO^: 226.0 ± 10.2 mg/dL, *P* < 0.01) and a 19% increase in 5 h fasting blood glucose (*ERα*^F/F^: 160.3 ± 6.0 vs. *ERα*^LivKO^: 187 ± 8.4 mg/dL, *P* < 0.05, Fig. 1e). Additionally, glucose tolerance and insulin sensitivity were significantly impaired in *ERα*^LivKO^ male mice (Fig. 1f, g). Hepatic insulin signaling was significantly impaired in *ERα*^LivKO^ male mice, indicated by a 62% (*P* < 0.0001) and a 34% (*P* < 0.05) decrease in insulin-induced phosphorylated AKT at S473 (pAKT-S473) and at T308 (pATK-T308), respectively. As expected, ERα protein levels were significantly decreased by 60% in the liver of *ERα*^LivKO^ male mice (*P* < 0.01, Fig. 1h and Fig. S2a). In female mice, hepatic ERα deletion led to a 10% increase in random feeding blood glucose (*ERα*^F/F^: 154.8 ± 5.3 vs. *ERα*^LivKO^: 183.4 ± 4.2 mg/dL, *P* < 0.01) and a 13% increase in 5 h fasting blood glucose (*ERα*^F/F^: 143.7 ± 5.5 vs. *ERα*^LivKO^: 162.1 ± 5.1 mg/dL, *P* < 0.05, Fig. 1i). Compared to *ERα*^F/F^ female mice, glucose tolerance and insulin sensitivity were impaired in *ERα*^LivKO^ female mice (Fig. 1j, k). Similar to the male mice, *ERα*^F/F^ female mice showed a significant decrease in insulin-induced AKT phosphorylation at S473 by 37% (*P* < 0.01) and T308 by 72% (*P* < 0.0001) in livers. ERα protein levels were downregulated by 72% (*P* < 0.01) in the liver of *ERα*^LivKO^ female mice (Fig. 1l and Fig. S2b). We further performed ovariectomy (OVX) in *ERα*^F/F^ and *ERα*^LivKO^ female mice. Compared to intact female mice, OVX female mice showed around 70% (*P* < 0.01) decrease in circulating E_2_ levels (Fig. S2c). OVX *ERα*^LivKO^ female mice showed a 17% increase in random feeding blood glucose (*P* < 0.05) and an 11% increase in 5 h fasting blood glucose (*P* < 0.05, Fig. S2d). Compared to OVX *ERα*^F/F^ female mice, glucose tolerance and insulin sensitivity were significantly impaired in OVX *ERα*^LivKO^ female mice (Fig. S2e, f). Hepatic insulin sensitivity was significantly attenuated in OVX *ERα*^LivKO^ female mice, indicated by a 51% decrease in pAKT-S473 (*P* < 0.01) and 46% decrease in pAKT-T308 (*P* < 0.0001). Hepatic ERα protein levels were significantly decreased by 94% (*P* < 0.001, Fig. S2g, h). Considering the effect of hepatic ERα in male and OVX female mice that have a low circulating estrogen level, we proposed that hepatic ERα regulates glucose homeostasis and insulin sensitivity in male mice through a ligand-independent mechanism.

In addition to ERα, ERβ also mediates the function of estrogen. We generated hepatic ERβ knockout (*ERβ*^LivKO^) mice and evaluated its role in the regulation of glucose homeostasis. We found that hepatic ERβ knockout had no significant effect on random feeding and overnight fasting blood glucose in both male and female mice (Fig. S2i, l). Compared to control mice, glucose tolerance and insulin sensitivity were not significantly impaired in both *ERβ*^LivKO^ male and female mice (Fig. S2j, k, m–o). Taken together, these results indicate that hepatic ERα regulates glucose tolerance and insulin sensitivity in a ligand-independent manner in male mice.

### Deletion of hepatic ERα impairs glucose tolerance and insulin sensitivity in both DIO male and female mice

**Fig. 2:**
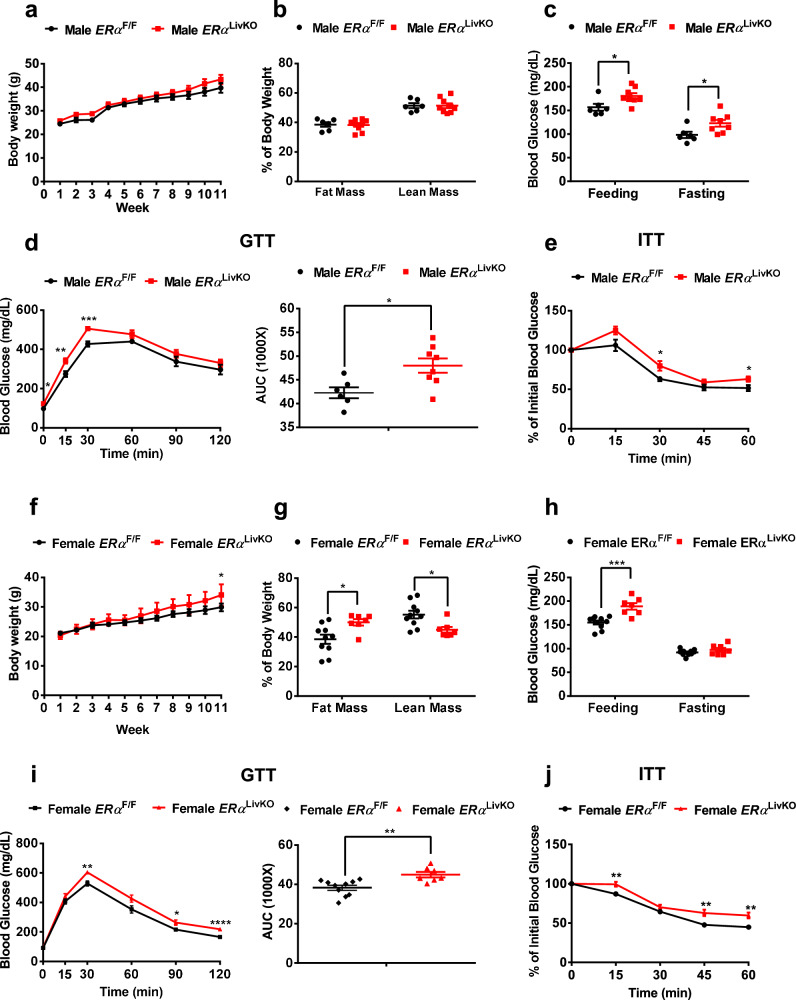
Deletion of hepatic ERα impairs glucose tolerance and insulin sensitivity in both DIO male and female mice. **a** Body weight of *ERα*^F/F^ and *ERα*^LivKO^ male mice treated with HFD, *n* = 6 (*ERα*^F/F^) and 9 (*ERα*^LivKO^) mice/group. **b** Body composition of *ERα*^F/F^ and *ERα*^LivKO^ male mice treated with HFD, *n* = 6 (*ERα*^F/F^) and 9 (*ERα*^LivKO^) mice/group. **c** Random feeding and 16 h fasting blood glucose in *ERα*^F/F^ and *ERα*^LivKO^ male mice treated with HFD, *n* = 6 (*ERα*^F/F^) and 8 (*ERα*^LivKO^) mice/group; feeding blood glucose, *P* = 0.0275; 16 h fasting blood glucose, *P* = 0.0303. **d** Glucose tolerance tests in *ERα*^F/F^ and *ERα*^LivKO^ male mice treated with HFD, *n* = 6 (*ERα*^F/F^) and 8 (*ERα*^LivKO^) mice/group; 0 min, *P* = 0.0303; 15 min, *P* = 0.0064; 30 min, *P* = 0.0008; AUC, *P* = 0.0149. **e** Insulin tolerance tests in *ERα*^F/F^ and *ERα*^LivKO^ male mice treated with HFD, *n* = 6 (*ERα*^F/F^) and 8 (*ERα*^LivKO^) mice/group; 30 min, *P* = 0.0461; 60 min, *P* = 0.0443. **f** Body weight of *ERα*^F/F^ and *ERα*^LivKO^ female mice treated with HFD, *n* = 7 (*ERα*^LivKO^) and 10 (*ERα*^F/F^) mice/group; *P* = 0.0494. **g** Body composition of *ERα*^F/F^ and *ERα*^LivKO^ female mice treated with HFD, *n* = 7 (*ERα*^LivKO^) and 10 (*ERα*^F/F^) mice/group; fat mass, *P* = 0.0146; lean mass, *P* = 0.0115. **h** Random feeding and 16 h fasting blood glucose in *ERα*^F/F^ and *ERα*^LivKO^ female mice treated with HFD, *n* = 7 (*ERα*^LivKO^) and 10 (*ERα*^F/F^) mice/group; random feeding blood glucose, *P* = 0.0006. **i** Glucose tolerance tests in *ERα*^F/F^ and *ERα*^LivKO^ female mice treated with HFD, *n* = 7 (*ERα*^LivKO^) and 10 (*ERα*^F/F^) mice/group; 30 min, *P* = 0.0039; 90 min, *P* = 0.0340; 120 min, *P* < 0.0001; AUC, *P* = 0.0036. **j** Insulin tolerance tests in *ERα*^F/F^ and *ERα*^LivKO^ female mice treated with HFD, *n* = 7 (*ERα*^LivKO^) and 10 (*ERα*^F/F^) mice/group; 15 min, *P* = 0.0088; 45 min, *P* = 0.0041; 60 min, *P* = 0.0028. Data are presented as mean ± SEM. *P < 0.05, **P < 0.01, ***P < 0.001, ****P < 0.0001, unpaired Two-tailed Student’s t test. Source data are provided as a Source Data file.

To further detect the role of hepatic ERα in the pathogenesis of diabetes, we fed male and female *ERα*^F/F^ and *ERα*^LivKO^ mice a high-fat diet (HFD) for 11 weeks. In diet-induced obesity (DIO) male mice, hepatic ERα deficiency did not change body weight and body mass (Fig. 2a, b). Compared to *ERα*^F/F^ DIO male mice, *ERα*^LivKO^ DIO male mice showed a 12% increase in random feeding blood glucose and a 25% increase in overnight fasting blood glucose (*P* < 0.05, Fig. 2c). Additionally, glucose tolerance and insulin sensitivity were significantly impaired in *ERα*^LivKO^ DIO male mice (Fig. 2d, e). In DIO female mice, hepatic ERα deficiency led to a significant increase in body weight at the 11^th^ week of HFD treatment (Fig. 2f). *ERα*^LivKO^ DIO female mice showed a 30% increase in fat mass and a 19% decrease in lean mass (*P* < 0.05, Fig. 2g). In female DIO mice, hepatic ERα deficiency led to a 22% increase in random feeding blood glucose (*P* < 0.001) and a 6% increase in overnight fasting blood glucose (Fig. 2h). Compared to *ERα*^F/F^ DIO female mice, glucose tolerance and insulin sensitivity were significantly attenuated in *ERα*^LivKO^ DIO female mice (Fig. 2i, j). These results indicate that hepatic ERα plays a protective role in diet-induced glucose dysregulation and insulin resistance.

### Hepatic ERα regulates glucose tolerance and insulin sensitivity in an IRS1/2-dependent manner in male mice

**Fig. 3:**
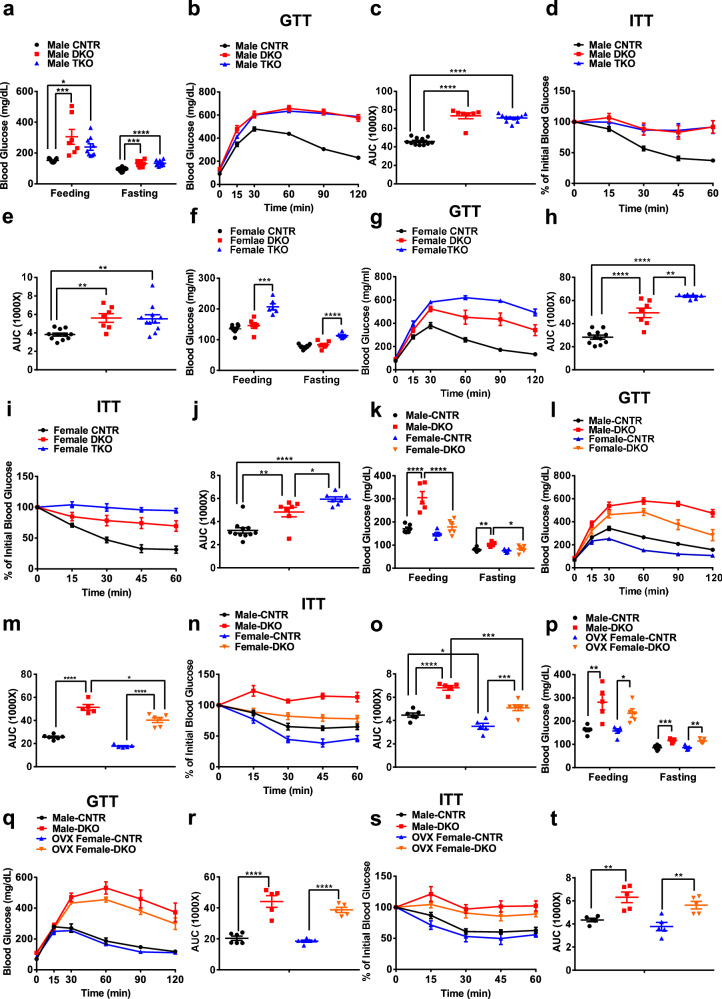
Hepatic ERα regulates glucose homeostasis and insulin sensitivity in an IRS1/2-independent manner. **a** Random feeding and 16 h fasting blood glucose levels in CNTR, DKO, and TKO male mice under regular chow diet, *n* = 7 (DKO), 9 (TKO), and 11 (control) mice/group; for feeding blood glucose, CNTR versus DKO, *P* = 0.0007, CNTR versus TKO, *P* = 0.0379; for fasting blood glucose, CNTR versus DKO, *P* = 0.0002, CNTR versus TKO, *P* < 0.0001. **b**, **c** Glucose tolerance tests in in control, DKO, and TKO male mice under regular chow diet, *n* = 7 (DKO) and 11 (control and TKO) mice/group; CNTR versus DKO, *P* < 0.0001, CNTR versus TKO, *P* < 0.0001. **d**, **e** Insulin tolerance tests in control, DKO, and TKO male mice under regular chow diet, *n* = 7 (DKO) and 11 (control and TKO) mice/group; CNTR versus DKO, *P* = 0.0096, CNTR versus TKO, *P* = 0.0053. **f** Random feeding and 16 h fasting blood glucose levels in control, DKO, and TKO female mice under regular chow diet, *n* = 6 (DKO and TKO) and 8 (control) mice/group; for feeding blood glucose, DKO versus TKO, *P* = 0.0002; for fasting blood glucose, DKO versus TKO, *P* < 0.0001. **g**, **h** Glucose tolerance tests in in control, DKO, and TKO female mice under regular chow diet, *n* = 7 (DKO and TKO) and 11 (control) mice/group; CNTR versus DKO, *P* < 0.0001, CNTR versus TKO, *P* < 0.0001, DKO versus TKO, *P* = 0.0024. **i**, **j** Insulin tolerance tests in control, DKO, and TKO female mice under regular chow diet, *n* = 7 (DKO and TKO) and 11 (control) mice/group; CNTR versus DKO, *P* = 0.0016, CNTR versus TKO, *P* < 0.0001, DKO versus TKO, *P* = 0.0452. **k** Random feeding and 16 h fasting blood glucose levels in control and DKO male/female mice under regular chow diet, *n* = 5 (Male-DKO and Female-CNTR) and 6 (Male-CNTR and Female-DKO) mice/group; for feeding blood glucose, Male-CNTR versus Male-DKO, *P* < 0.0001, Male-DKO versus Female-DKO, *P* < 0.0001; for fasting blood glucose, Male-CNTR versus Male-DKO, *P* = 0.0095, Male-DKO versus Female-DKO, *P* = 0.0270. **l**, **m** Glucose tolerance tests in control and DKO male/female mice under regular chow diet, *n* = 5 (Male-DKO and Female-CNTR) and 6 (Male-CNTR and Female-DKO) mice/group; Male-CNTR versus Male-DKO, *P* < 0.0001, Female-CNTR versus Female-DKO, *P* < 0.0001, Male-DKO versus Female-DKO, *P* = 0.0242. **n**, **o** Insulin tolerance tests in control and DKO male/female mice under regular chow diet, *n* = 5 (Male-DKO and Female-CNTR) and 6 (Male-CNTR and Female-DKO) mice/group; Male-CNTR versus Male-DKO, *P* < 0.0001, Female-CNTR versus Female-DKO, *P* = 0.0005, Male-CNTR versus Female-CNTR, *P* = 0.0318, Male-DKO versus Female-DKO, *P* = 0.0003. **p** Random feeding and 16 h fasting blood glucose levels in control and DKO male/OVX female mic under regular chow diet, *n* = 5 (Male-DKO, OVX Female-CNTR, and OVX Female-DKO) and 6 (Male-CNTR) mice/group; for feeding blood glucose, Male-CNTR versus Male-DKO, *P* = 0.0013, OVX Female-CNTR versus OVX Female-DKO, *P* = 0.0473, for fasting blood glucose, Male-CNTR versus Male-DKO, *P* = 0.0004, OVX Female-CNTR versus OVX Female-DKO, *P* = 0.0032. **q**, **r** Glucose tolerance tests in control and DKO male/OVX female mice under regular chow diet, *n* = 5 (Male-DKO, OVX Female-CNTR, and OVX Female-DKO) and 6 (Male-CNTR) mice/group; Male-CNTR versus Male-DKO, *P* < 0.0001, OVX Female-CNTR versus OVX Female-DKO, *P* < 0.0001. **s**, **t** Insulin tolerance tests in control and DKO male/OVX female mice under regular chow diet, *n* = 5 mice/group; Male-CNTR versus Male-DKO, *P* = 0.0045, OVX Female-CNTR versus OVX Female-DKO, *P* = 0.0075. Data are presented as mean ± SEM. *P < 0.05, **P < 0.01, ***P < 0.001, ****P < 0.0001, One-way ANOVA (**a**–**j**) or Two-way ANOVA with Tukey’s multiple comparisons test (**k**–**t**). CNTR: Control. Source data are provided as a Source Data file.

Previous study showed that ERα protected IRS1 and IRS2 against ubiquitination-induced degradation independent of E_2_^16^. We thus reasoned that the ligand-independent effect of ERα on glucose homeostasis may be mediated by IRS1 and IRS2. To detect this hypothesis, we generated liver-specific IRS1 and IRS2 double knockout (DKO) and liver-specific IRS1, IRS2, and ERα triple knockout (TKO) mice. In male mice, compared to control (CNTR) mice, liver IRS1 and ISR2 deletion led to a 102% increase in random feeding blood glucose (CNTR: 151.5 ± 3.5 vs. DKO: 305.6 ± 48.3 mg/dL, *P* < 0.001) and a 33% increase in overnight fasting blood glucose (CNTR: 95.7 ± 3.4 vs. DKO: 127.0 ± 11.6 mg/dL, *P* < 0.001). Hepatic ERα deletion did not further significantly increase random feeding and overnight fasting blood glucose in DKO male mice (Fig. 3a). In addition, DKO male mice showed severe glucose intolerance (*P* < 0.0001) and insulin resistance (*P* < 0.01), as compared to CNTR male mice. Hepatic ERα deletion in DKO male mice had no significant effect on glucose tolerance and insulin sensitivity compared to DKO mice (Fig. 3b–e). Hepatic insulin sensitivity was significantly attenuated in DKO and TKO male mice, indicated by ~86% decreases in insulin-induced AKT phosphorylation at S473 (*P* < 0.0001) and T308 (*P* < 0.0001). Compared to DKO male mice, hepatic insulin sensitivity was not significantly affected in TKO male mice (Fig. S3a). Gene expression analysis showed that compared to CNTR male mice, mRNA expression levels of gluconeogenic genes (*G6pc* and *Pck1*) were significantly increased by 4.2- and 2.9-fold, respectively, in the livers of DKO and TKO male mice. TKO male mice did not show significant differences in mRNA expression levels of *G6pc* and *Pck1* in liver, as compared to DKO male mice. As expected, expression levels of *Irs1*, *Irs2*, and *ERα* were significantly decreased in DKO and TKO male mouse livers (Fig. S3b). In female mice, liver IRS1 and IRS2 deletion had no significant effects on random feeding (CNTR: 135.3 ± 5.0 vs. DKO: 146.3 ± 9.1 mg/dL) and overnight fasting (CNTR: 77.6 ± 2.5 vs. DKO: 83.2 ± 6.0 mg/dL) blood glucose. However, TKO female mice showed a 45% increase in random feeding blood glucose (TKO: 207.0 ± 9.6 mg/dL, *P* < 0.001) and 39% increase in overnight fasting blood glucose (TKO: 113.3 ± 3.1 mg/dL, *P* < 0.0001), as compared to DKO female mice (Fig. 3f). Glucose tolerance and insulin sensitivity were significantly impaired in DKO female mice, compared to CNTR female mice. Notably, liver IRS1, IRS2, and ERα triple knockout led to a significant decrease in glucose tolerance (*P* < 0.01) and insulin sensitivity (*P* < 0.05), as compared to DKO female mice (Fig. 3g–j). Additionally, DKO female mice showed a 79% and 95% reduction in insulin-induced AKT phosphorylation at S473 and T308, respectively (*P* < 0.0001). Liver ERα deletion in DKO mice further decreased insulin-induced pAKT-S473 and pAKT-T308 by 70% (*P* < 0.01) and 62%, respectively, as compared to DKO female mice (Fig. S3c). DKO female mice showed an increasing trend in the mRNA expression levels of *G6pc* and a significant increase in the mRNA expression levels of *Pck1* in the livers, as compared to CNTR female mice. Hepatic ERα deletion significantly increased mRNA expression levels of *G6pc* by 1.4-fold (*P* < 0.05) and *Pck1* by 80% (*P* < 0.01) in the livers of DKO female mice. The expression levels of *Irs1*, *Irs2*, and *ERα* were significantly decreased in the livers of DKO and TKO female mice (Fig. S3d).

To further investigate the sex difference in DKO mice, we analyzed glucose homeostasis in male and female DKO mice. Consistently, DKO male mice showed significant increases in random feeding (*P* < 0.0001) and overnight fasting (*P* < 0.01) blood glucose compared to CNTR male mice. On the other hand, compared to CNTR female mice, DKO female mice did not show significant increases in blood glucose under both random feeding and overnight fasting conditions. Compared to DKO male mice, DKO female mice showed a 41% decrease in random feeding blood glucose (Male DKO: 306.2 ± 26.9 vs. Female DKO: 178.7 ± 11.3 mg/dL, *P* < 0.0001) and 18% decrease in overnight fasting blood glucose (Male DKO: 103.8 ± 4.5 vs. Female DKO: 83.3 ± 6.1 mg/dL, *P* < 0.05, Fig. 3k). CNTR female mice showed better glucose tolerance and insulin sensitivity than CNTR male mice. However, glucose tolerance and insulin resistance were significantly impaired in both DKO male and female mice. Compared to DKO male mice, glucose tolerance and insulin sensitivity were significantly improved by 21% (*P* < 0.05) and 25% (*P* < 0.001), respectively, in DKO female mice (Fig. 3l–o). In the liver, insulin sensitivity was significantly impaired in both DKO male and female mice. DKO female mice showed an improvement in hepatic insulin sensitivity compared to that in DKO male mice, indicated by a 123% and 35% increase in insulin-induced pAKT-S473 and pAKT-T308, respectively (*P* < 0.01, Fig. S3e). Consistently, the mRNA expression levels of *G6pc* and *Pck1* were significantly increased in the livers of DKO male mice compared to CNTR male mice. The DKO female mice showed a significant decrease in mRNA expression levels of *G6pc* by 59% (*P* < 0.01) and *Pck1* by 39% (*P* < 0.05), as compared to DKO male mice (Fig. S3f). To test whether the sex difference in glucose homeostasis in DKO mice is mediated by ovary-secreted hormones, such as estrogen, we performed ovariectomy (OVX) in DKO female mice. Consistent with results from DKO male mice, DKO OVX female mice showed significant increases in both random feeding (CNTR OVX: 157.2 ± 9.6 vs. DKO OVX: 234.2 ± 18.8 mg/dL, *P* < 0.05) and overnight fasting (CNTR OVX: 86.2 ± 3.9 vs. DKO OVX: 114.4 ± 4.4 mg/dL, *P* < 0.01) blood glucose (Fig. 3p). Both DKO male and OVX female mice exhibited severe glucose intolerance (*P* < 0.0001) and insulin resistance (*P* < 0.01) compared to CNTR mice. However, there were no significant differences in both glucose tolerance and insulin sensitivity between DKO male and OVX female mice (Fig. 3q–t). Consistently, hepatic insulin signaling was diminished in both DKO male and OVX female mice (*P* < 0.0001) and we did not observe significant differences in insulin-stimulated ATK phosphorylation between DKO male and OVX female mouse livers (Fig. S3g). Consistently, DKO OVX female mice showed comparable mRNA expression levels of *G6pc* and *Pck1* in the livers, as compared to DKO male mice (Fig. S3h). Of note, E_2_ supplement significantly improved glucose tolerance (*P* < 0.05) and insulin sensitivity (*P* < 0.05) in the DKO OVX female mice (Fig. S3i–l). Consistently, hepatic insulin signaling was significantly enhanced by E_2_ supplement in the DKO OVX female mice (Fig. S3m). The mRNA expression levels of *G6pc* (*P* < 0.05) and *Pck1* (*P* < 0.05) were significantly decreased by E_2_ supplement in the livers of DKO OVX female mice (Fig. S3h). These results indicate that the effect of hepatic ERα on insulin sensitivity is mediated through IRS1 and IRS2 in male mice, while in female mice, ovary hormone estrogen regulates insulin sensitivity independent of IRS1 and IRS2. Taken together, IRS1 and IRS2 are required by the ligand-independent effect of ERα on insulin sensitivity in male mice.

### ERα stimulates hepatic insulin signaling through increasing IRS1 protein stability

**Fig. 4:**
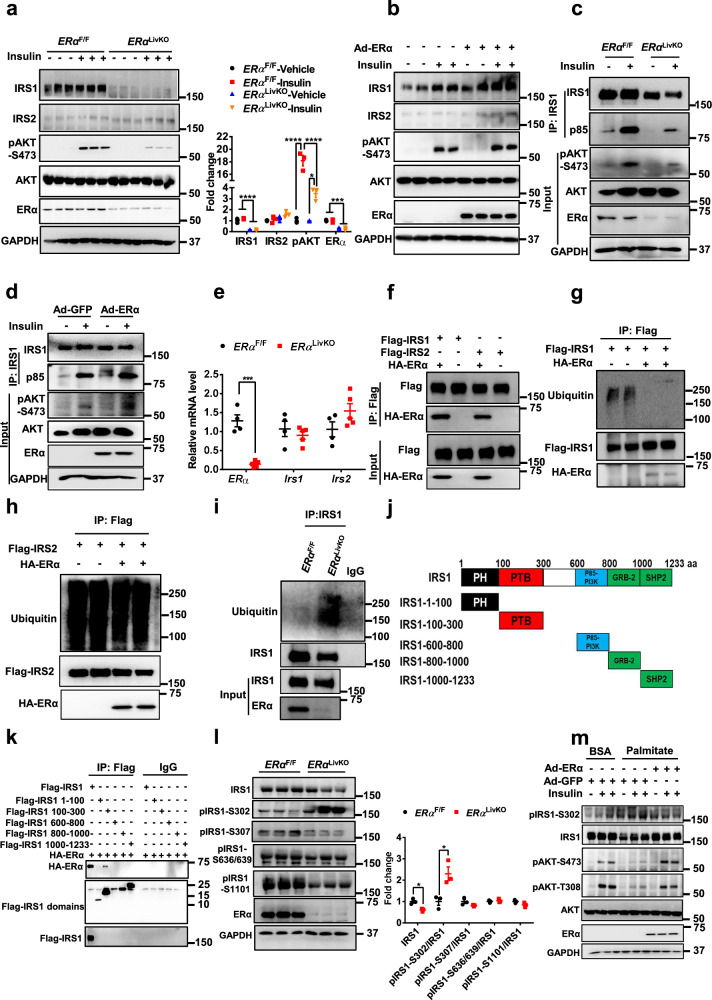
ERα increases IRS1 protein stability and promotes hepatic insulin sensitivity. **a** Insulin signaling activity in primary hepatocytes from *ERα*^F/F^ and *ERα*^LivKO^ male mice, *n* = 3 independent cells; for IRS1, *ERα*^F/F^-Vehicle versus *ERα*^LivKO^-Vehicle, *P* < 0.0001, *ERα*^F/F^-Insulin versus *ERα*^LivKO^-Insulin, *P* < 0.0001; for pAKT-S473, *ERα*^F/F^-Vehicle versus *ERα*^F/F^-Insulin, *P* < 0.0001, *ERα*^LivKO^-Vehicle versus *ERα*^LivKO^-Insulin, *P* = 0.0186, *ERα*^F/F^-Insulin versus *ERα*^LivKO^-Insulin, *P* < 0.0001; for *ERα*, *ERα*^F/F^-Vehicle versus *ERα*^LivKO^-Vehicle, *P* = 0.0002, *ERα*^F/F^-Insulin versus *ERα*^LivKO^-Insulin, *P* = 0.0001. **b** Effect of ERα gain-of-function on hepatic insulin sensitivity. The experiments were repeated independently twice. Representative blots were shown. **c** Effect of ERα deletion on insulin-induced IRS1 and p85 interaction. The experiments were repeated independently twice. Representative blots were shown. **d** Effect of ERα gain-of-function on insulin-induced IRS1 and p85 interaction. The experiments were repeated independently twice. Representative blots were shown. **e** Effect of ERα deletion on mRNA expression of *Irs1* and *Irs2* in primary mouse hepatocytes, *n* = 4 (*ERα*^F/F^) and 5 (*ERα*^LivKO^); for *ERα*, *P* = 0.0001. **f** Interaction between ERα and IRS1 or IRS2 in HEK293T cells. The experiments were repeated independently three times. Representative blots were shown. **g** Effect of ERα on IRS1 ubiquitination in HEK293T cells. The experiments were repeated independently twice. Representative blots were shown. **h** Effect of ERα on IRS2 ubiquitination in HEK293T cells. The experiments were repeated independently twice. Representative blots were shown. **i** Effect of ERα deletion on IRS1 ubiquitinat**i**on in primary mouse hepatocytes. The experiments were repeated independently twice. Representative blots were shown. **j** Diagram of IRS1 and its truncated domains. **k** Interaction between ERα and IRS1 domains in HEK293T cells. The experiments were repeated independently three times. Representative blots were shown. **l** Effect of ERα deletion on IRS1 phosphorylation in primary mouse hepatocytes, *n* = 3 independent cells; for IRS1, *P* = 0.0102; for pIRS1-S302, *P* = 0.0236. **m** Effect of ERα gain-of-function on palmitate-induced insulin resistance in primary mouse hepatocytes. The experiments were repeated independently twice. Representative blots were shown. Data are presented as mean ± SEM. *P < 0.05, ***P < 0.001, ****P < 0.0001, unpaired Two-tailed Student’s t test (e and l), Two-way ANOVA with Tukey’s multiple comparisons test (a). Source data are provided as a Source Data file.

We next explored how ERα regulates the expression of IRS1 and IRS2 to improve hepatic insulin sensitivity. In mouse primary hepatocytes, ERα deletion decreased IRS1 protein levels by 80% (*P* < 0.0001) but had no significant effect on IRS2 protein abundance. Insulin-stimulated AKT phosphorylation at S473 was diminished by 81% (*P* < 0.0001) in *ERα*^LivKO^ hepatocytes (Fig. 4a). ERα gain-of-function increased IRS1 and pAKT-S473 protein levels but had a limited effect on IRS2 protein levels in control primary hepatocytes (Fig. 4b). Insulin-induced interaction between IRS1 and p85 was attenuated in *ERα*^LivKO^ hepatocytes (Fig. 4c). ERα overexpression in control hepatocytes enhanced the interaction between IRS1 and p85 (Fig. 4d). Compared to control hepatocytes, *Irs1* and *Irs2* mRNA levels were not significantly affected in *ERα*^LivKO^ hepatocytes (Fig. 4e), which indicates that ERα regulates IRS1 protein expression through a post-translational modification. To test this hypothesis, we treated control and *ERα*^LivKO^ hepatocytes with MG132, a proteasome inhibitor and found that ERα deficiency significantly decreased IRS1 protein levels by 30% (*P* < 0.01) and MG132 treatment significantly increased IRS1 proteins levels (*P* < 0.05) in *ERα*^LivKO^ hepatocytes (Fig. S4a). We found that ERα interacted with both IRS1 and IRS2 (Fig. 4f). ERα largely attenuated the ubiquitination of IRS1 but had a limited effect on IRS2 ubiquitination (Fig. 4g, h). Consistently, ERα deletion increased IRS1 ubiquitination in primary hepatocytes (Fig. 4i). E_2_ treatment had no effect on the interaction between IRS1 and ERα (Fig. S4b), suggesting that ERα interacts with IRS1 in a ligand-independent manner. To further investigate how ERα regulates IRS1 ubiquitination, we constructed plasmids of truncated IRS1 domains based on its functional unit (Fig. 4j). We found that ERα interacted with the IRS1 100-300 domain (Fig. 4k). Furthermore, we detected the effect of ERα on the serine phosphorylation of IRS1 that leads to ubiquitin-mediated degradation. ERα deletion decreased IRS1 protein levels by 39% (*P* < 0.05) and stimulated IRS1-S302 phosphorylation by 130% (*P* < 0.05) in hepatocytes. However, IRS1 phosphorylation at S307, S636/639, and S1101 were not significantly affected in *ERα*^LivKO^ hepatocytes (Fig. 4l). Palmitate treatment stimulated IRS1 phosphorylation at S302 and reduced IRS1 protein levels and insulin-induced AKT phosphorylation. ERα overexpression improved palmitate-induced insulin resistance, indicated by a decrease in pIRS1-S302 and increases in IRS1, pAKT-S473, and pAKT-T308 protein levels (Fig. 4m). Taken together, these results indicate that ERα interacts with IRS1 and increases IRS1 protein levels, thereby stimulating insulin sensitivity.

### ERα 1-280 domain increases insulin sensitivity through enhancing IRS1 protein stability

**Fig. 5:**
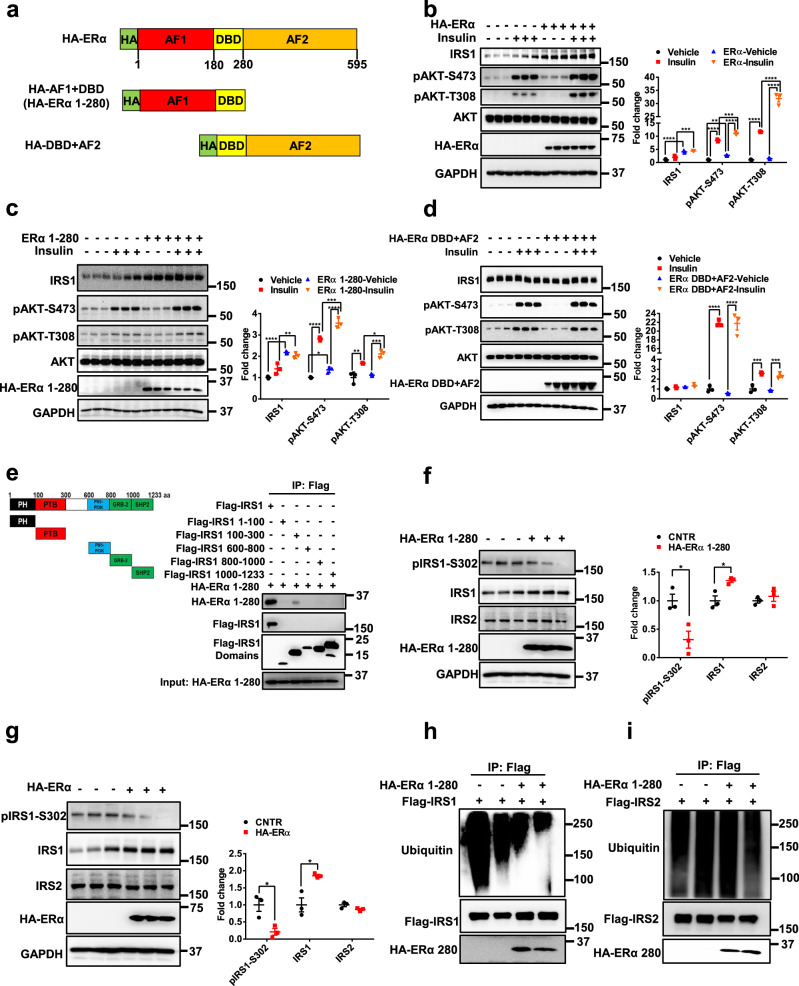
ERα 1-280 domain increases IRS1 protein stability and enhances insulin signaling. **a** Diagram of truncated ERα protein domains. **b** Effect of ERα on insulin sensitivity in HepG2 cells, *n* = 3 independent cells; for IRS1, Vehicle versus ERα-Vehicle, *P* < 0.0001, Insulin versus ERα-Insulin, *P* = 0.0005; for pAKT-S473, Vehicle versus ERα-Vehicle, *P* = 0.0025, Vehicle versus Insulin, *P* < 0.0001, ERα-Vehicle versus ERα-Insulin, *P* < 0.0001, Insulin versus ERα-Insulin, *P* = 0.0001; for pAKT-T308, Vehicle versus Insulin, *P* < 0.0001, ERα-Vehicle versus ERα-Insulin, *P* < 0.0001, Insulin versus ERα-Insulin, *P* < 0.0001. **c** Effect of ERα 1-280 on insulin sensitivity in HepG2 cells, *n* = 3 independent cells; for IRS1, Vehicle versus ERα 1-280-Vehicle, *P* < 0.0001, Insulin versus ERα 1-280-Insulin, *P* = 0.0034; for pAKT-S473, Vehicle versus ERα 1-280-Vehicle, *P* = 0.0457, Vehicle versus Insulin, *P* < 0.0001, ERα 1-280-Vehicle versus ERα 1-280-Insulin, *P* < 0.0001, Insulin versus ERα 1-280-Insulin, *P* = 0.0007; for pAKT-T308, Vehicle versus Insulin, *P* = 0.0040, ERα 1-280-Vehicle versus ERα 1-280-Insulin, *P* = 0.0003, Insulin versus ERα 1-280-Insulin, *P* = 0.0397. **d** Effect of ERα DBD + AF2 on insulin sensitivity in HepG2 cells, *n* = 3 independent cells; for pAKT-S473, Vehicle versus Insulin, *P* < 0.0001, ERα DBD + AF2-Vehicle versus ERα DBD + AF2-Insulin, *P* < 0.0001; for pAKT-T308, Vehicle versus Insulin, *P* = 0.0002, ERα DBD + AF2-Vehicle versus ERα DBD + AF2-Insulin, *P* = 0.0003. **e** Interaction between ERα 1-280 and IRS1 protein domains. The experiments were repeated independently twice. Representativ**e** blots were shown. **f** Effect of ERα 1-280 on IRS1 phosphorylation at S302 in HepG2 cells, *n* = 3 independent cells; for pIRS1-S302, *P* = 0.0227; for IRS1, *P* = 0.0187. **g** Effect of ERα on IRS1 phosphorylation at S302 in HepG2 cells, *n* = 3 independent cells; for pIRS1-S302, *P* = 0.0207; for IRS1, *P* = 0.0154. **h** Effect of ERα 1-280 on IRS1 ubiquitination i*n* HEK293T cells. The experiments were repeated independently twice. Representative blots were shown. **i** Effect of ERα 1-280 on IRS2 ubiquitination in HEK293T cells. The experiments were repeated independently twice. Representative blots were shown. Data are presented as mean ± SEM. *P < 0.05, **P < 0.01, ***P < 0.001, ****P < 0.0001, unpaired Two-tailed Student’s t test (**f**, **g**), Two-way ANOVA with Tukey’s multiple comparisons test (**b**, **c**, **d**). CNTR: Control. Source data are provided as a Source Data file.

ERα is composed of different function domains, including AF (activation function) 1, DBD (DNA binding domain), and AF2. To further investigate how ERα enhances IRS1 protein stability, we generated different ERα domain plasmids and tested their effects on hepatic insulin sensitivity (Fig. 5a). Consistent with the results in primary hepatocytes, ERα overexpression significantly increased insulin sensitivity in HepG2 cells, indicated by significant increases in IRS1 protein levels and insulin-induced AKT phosphorylation (*P* < 0.0001; Fig. 5b). ERα AF1 + DBD (1-280) overexpression significantly increased IRS1 protein levels by 43% (*P* < 0.01) and insulin-induced AKT phosphorylation by 26% (*P* < 0.05) in HepG2 cells (Fig. 5c). However, overexpression of ERα DBD + AF2 did not significantly stimulate insulin sensitivity (Fig. 5d). In line with ERα, ERα 1-280 showed interaction with the IRS1 100-300 domain (Fig. 5e). Gain-of-function of ERα 1-280 decreased pIRS1-S302 by 62% (*P* < 0.05) and increased IRS1 protein abundance by 36% (*P* < 0.05), but had no effect on IRS2 protein abundance in HepG2 cells (Fig. 5f). Consistently, HepG2 cells with ERα overexpression showed a 79% decrease in pIRS1-S302 (*P* < 0.05), 85% increase in IRS1 protein levels (*P* < 0.05), and no significant effect on IRS2 levels (Fig. 5g). The ubiquitination of IRS1 but not IRS2 was attenuated by ERα 1-280 (Fig. 5h, i).

We then detected the role of ERα and ERα 1-280 in the regulation of insulin sensitivity in WT mice. Adenovirus (Ad)-mediated ERα overexpression led to a 15% decrease in random feeding blood glucose (Ad-GFP: 196.0 ± 10.5 vs. Ad-ERα: 167.5 ± 4.3 mg/dL; *P* < 0.05) in WT male mice (Fig. S5a). In mice with ERα overexpression, glucose tolerance and insulin sensitivity were significantly improved by 16% and 22%, respectively (*P* < 0.05, Fig. S5b–e). Moreover, hepatic insulin sensitivity was increased by ERα gain-of-function, indicated by increases in IRS1 protein levels by 46% (*P* < 0.01), insulin-induced pAKT-S473 by 55% (*P* < 0.01), and pAKT-T308 by 22% (*P* < 0.01, Fig. S5f, g). We further injected Ad-ERα 1-280 into WT mice and found that ERα 1-280 overexpression had no significant effect on the blood glucose in both random feeding and overnight fasting male WT mice (Fig. S5h). During the glucose and insulin challenge, glucose disposal rate was significantly increased in ERα 1-280 overexpressed male WT mice (Fig. S5i–l). Accordingly, ERα 1-280 gain-of-function increased IRS1 abundance by 110% and enhanced insulin-induced AKT phosphorylation by ~50% in the liver of male WT mice (*P* < 0.01, Fig. S5m, n). In female WT mice, ERα 1-280 overexpression had no significant effect on the blood glucose under both feeding and fasting conditions (Fig. S5o). Glucose tolerance and insulin sensitivity were significantly improved in female WT mice infected with Ad-ERα 1-280 (Fig. S5p-r). Furthermore, hepatic insulin sensitivity was enhanced by ERα 1-280 overexpression in the liver of female WT mice, as evidenced by a 160% increase in IRS1 protein levels (*P* < 0.0001) and ~20-30% increase in insulin-stimulated AKT phosphorylation (*P* < 0.01, Fig. S5s, t). These results suggest that ERα 1-280 is an important domain to mediate ERα-stimulated insulin sensitivity in vivo and in vitro.

### ERα-derived AF1 peptide increases hepatic insulin sensitivity through IRS1

**Fig. 6:**
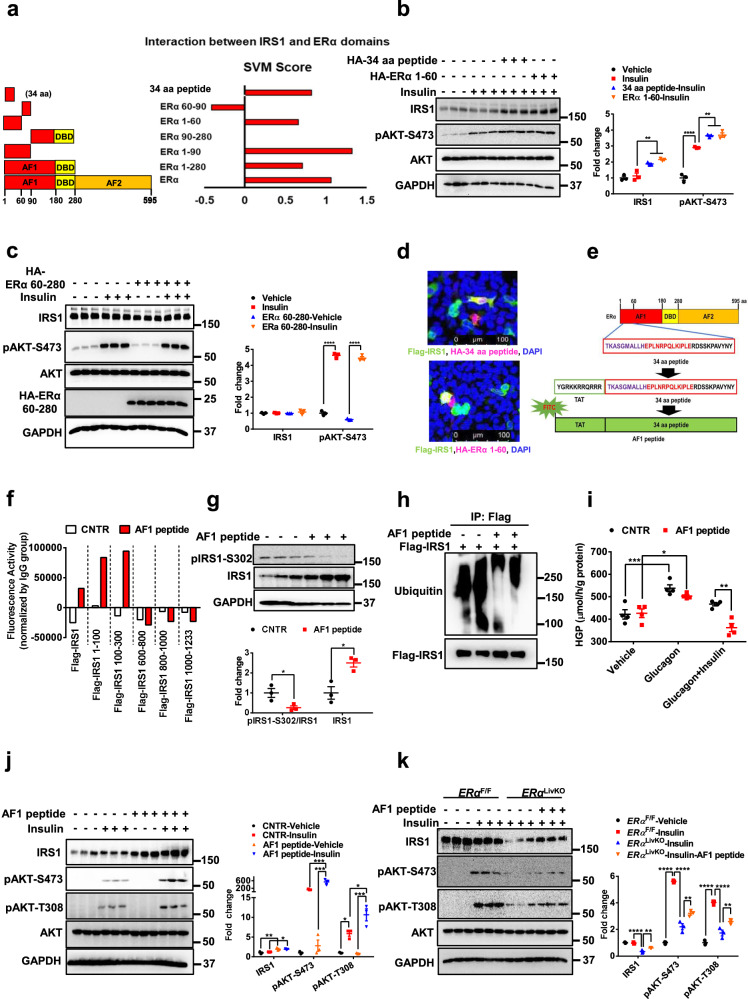
ERα-derived AF1 peptide increases hepatic insulin sensitivity through IRS1. **a** SVM score of interaction between IRS1 and ERα domains. **b** Effect of ERα 1-60 domain and AF1 peptide (34 aa) on insulin sensitivity in HepG2 cells, *n* = 3 independent cells; for IRS1, Insulin versus 34 aa peptide-Insulin, *P* = 0.0046, Insulin versus ERα 1-60-Insulin, *P* = 0.0006; for pAKT-S473, Vehicle versus Insulin, *P* < 0.0001, Insulin versus 34 aa peptide-Insulin, *P* = 0.0044, Insulin versus ERα 1-60-Insulin, *P* = 0.0030. **c** Effect of ERα 60-280 domain on insulin sensitivity in HepG2 cells, *n* = 3 indepe*n*dent cells; for pAKT-S473, Vehicle versus Insulin, *P* < 0.0001, ERα 60-280-Vehicle versus ERα 60-280-Insulin, *P* < 0.0001. **d** Immunofluorescence staining of AF1 peptide (34 aa) and ERα 1-60 domain in HEK293T cells. The experiments were repeated independently twice. Representative images were shown. **e** Amino acid sequence of FITC labeled AF1 peptide conjugated with TAT. **f** Interaction between AF1 peptide and IRS1 protein domains. The experiments were repeated independently twice. Representative results were shown. **g** Effect of AF1 peptide on IRS1 phosphorylation at S302 in primary mouse hepatocytes, *n* = 3 independent cells; for pIRS1-S302, *P* = 0.0377; for IRS1, *P* = 0.0156. **h** Effect of AF1 peptide on IRS1 ubiquitination in HEK293T cells. The experiments were repeated independently twice. Representative blots were shown. **i** Effect of AF1 peptide on insulin-induced suppression of HGP in primary hepatocytes upon glucagon treatment, *n* = 4 independent cells; CNTR-Vehicle versus CNTR-Glucagon, *P* = 0.0006, AF1 peptide-Vehicle versus AF1 peptide-Glucagon, *P* = 0.0272, CNTR-Glucagon-Insulin versus AF1 peptide-Glucagon-Insulin, *P* = 0.0019. **j** Effect of AF1 peptide on insulin sensitivity in primary mouse hepatocytes, *n* = 3 independent cells; for IRS1, CNTR-Vehicle versus AF1 peptide-Vehicle, *P* = 0.0067, CNTR-Insulin versus AF1 peptide-Insulin, *P* = 0.0314, for pAKT-S473, AF1 peptide-Vehicle versus AF1 peptide-Insulin, *P* = 0.0001, CNTR-Insulin versus AF1 peptide-Insulin, *P* = 0.0008; for pAKT-T308, CNTR-Vehicle versus CNTR-Insulin, *P* = 0.0154, AF1 peptide-Vehicle versus AF1 peptide-Insulin, *P* = 0.0002, CNTR-Insulin versus AF1 peptide-Insulin, *P* = 0.0167. **k** Effect of AF1 peptide on insulin sensitivity in control and ERα deficient primary mouse hepatocytes, *n* = 3 independent cells, for IRS1, *ERα*^F/F^-Insulin versus *ERα*^LivKO^-Insulin, *P* < 0.0001, *ERα*^LivKO^-Insulin versus *ERα*^LivKO^-Insuin-AF1 peptide, *P* = 0.0061, for pAKT-S473, *ERα*^F/F^-Vehicle versus *ERα*^F/F^-Insulin, *P* < 0.0001, *ERα*^F/F^-Insulin versus *ERα*^LivKO^-Insulin, *P* < 0.0001, *ERα*^LivKO^-Insulin versus *ERα*^LivKO^-Insulin-AF1 peptide, *P* = 0.0034; for pAKT-T308, *ERα*^F/F^-Vehicle versus *ERα*^F/F^-Insulin, *P* < 0.0001, *ERα*^F/F^-Insulin versus *ERα*^LivKO^-Insulin, *P* < 0.0001, *ERα*^LivKO^-Insulin versus *ERα*^LivKO^-Insulin-AF1 peptide, *P* = 0.0090. Data are presented as mean ± SEM. *P < 0.05, **P < 0.01, ***P < 0.001, ****P < 0.0001, unpaired Two-tailed Student’s *t* test (**g**), One-way ANOVA (**b**, **i**, **k**), Two-way ANOVA with Tukey’s multiple comparisons test (**c**, **j**). CNTR: Control. Source data are provided as a Source Data file.

Considering ERα 1-280 is an important domain in the regulation of insulin sensitivity, we analyzed its amino acid sequence with a goal of designing a peptide that functions as an insulin sensitizer. We performed computational analysis and calculated interaction score (SVM score) between ERα domain and IRS1. We set “0” as threshold (SVM score > 0 indicates interaction; SVM score <0 indicates no interaction) since it gives a high sensitivity, specificity, and accuracy. We found that the ERα 1-90, but not the 90-280, domain showed a higher interaction score with IRS1, indicating that ERα 1-90 has a higher potential to interact with IRS1. Further analysis of ERα 1-90 domain showed that ERα 1-60 domain and IRS1 had a high interaction score (Fig. 6a). To further shorten the functional peptide, we analyzed the amino acid of ERα 1-60 domain. The N and C terminals of ERα 1-60 domain (1-6 aa and 55-60 aa) are not essential for its interaction with IRS1, indicated by that ERα 7-54 domain showed a high interaction score with IRS1. However, deletion of ERα 7-12 aa or 49-54 aa impaired its interaction with IRS1, indicated by a low interaction score of ERα 13-54 and 7-48 domains. Finally, we narrowed down the ERα 7-54 domain into 34 aa by deleting QIQGENL and PLGEVYL sequences, which generates a higher interaction score with IRS1 (Fig. S6a). This ERα-derived 34 aa showed a high similarity (95.59%) among different species (Fig. S6b). To examine the effects of ERα 1-60 and 34-amino acid-peptide on insulin sensitivity, we performed gain-of-function experiments in HepG2 cells. Both overexpression of ERα 1-60 and 34-amino acid-peptide significantly increased IRS1 by ~80% (*P* < 0.01) and enhanced insulin-stimulated AKT phosphorylation by ~26% (*P* < 0.01) in HepG2 cells (Fig. 6b). However, the ERα 60-280 domain had no significant effect on IRS1 protein levels and insulin-induced AKT phosphorylation (Fig. 6c). The ERα 1-60 domain and 34-amino acid-peptide showed colocalization with IRS1, indicating a potential interaction (Fig. 6d). To further confirm the effect of 34-amino acid-peptide, we synthesized this peptide with a FITC-labeled TAT sequence at the N-terminal of the 34-aa-peptide (termed as AF1 peptide; Fig. 6e). The co-immunoprecipitation assay showed that AF1 peptide interacted with the IRS1 1-300 domain (Fig. 6f). AF1 peptide treatment significantly decreased pIRS1-S302 by 75% (*P* < 0.05) and increased IRS1 protein levels by 50% (*P* < 0.05) in WT primary hepatocytes (Fig. 6g). The ubiquitination of IRS1 was attenuated by AF1 peptide (Fig. 6h). AF1 peptide did not impair glucagon-induced hepatic glucose production (HGP) and significantly enhanced insulin-mediated suppression of HGP by 22% under glucagon challenge (*P* < 0.01) in WT primary hepatocytes (Fig. 6i). Consistently, AF1 peptide increased insulin sensitivity in WT primary hepatocytes, indicated by a 90% increase in IRS1 protein levels (*P* < 0.01) and enhancement in insulin-stimulated AKT phosphorylation at S473 by 210% (*P* < 0.001) and T308 by 82% (*P* < 0.05, Fig. 6j). To detect whether AF1 peptide can rescue ERα deficiency-induced hepatic insulin resistance, we treated ERα deficient primary hepatocytes with the AF1 peptide. Insulin sensitivity was significantly enhanced in ERα deficient primary hepatocytes by AF1 peptide, indicated by increases in IRS1, pAKT-S473, and pAKT-T308 protein levels by 80%, 46%, and 50%, respectively (*P* < 0.01). Compared to *ERα*^F/F^ hepatocytes, the AF1 peptide partially rescued ERα deficiency-induced insulin resistance (Fig. 6k). Collectively, these results indicate that ERα-derived AF1 peptide significantly increases hepatic insulin sensitivity through enhancing IRS1 protein stability.

### AF1 peptide improves glucose tolerance and insulin sensitivity in diabetic mouse models

**Fig. 7:**
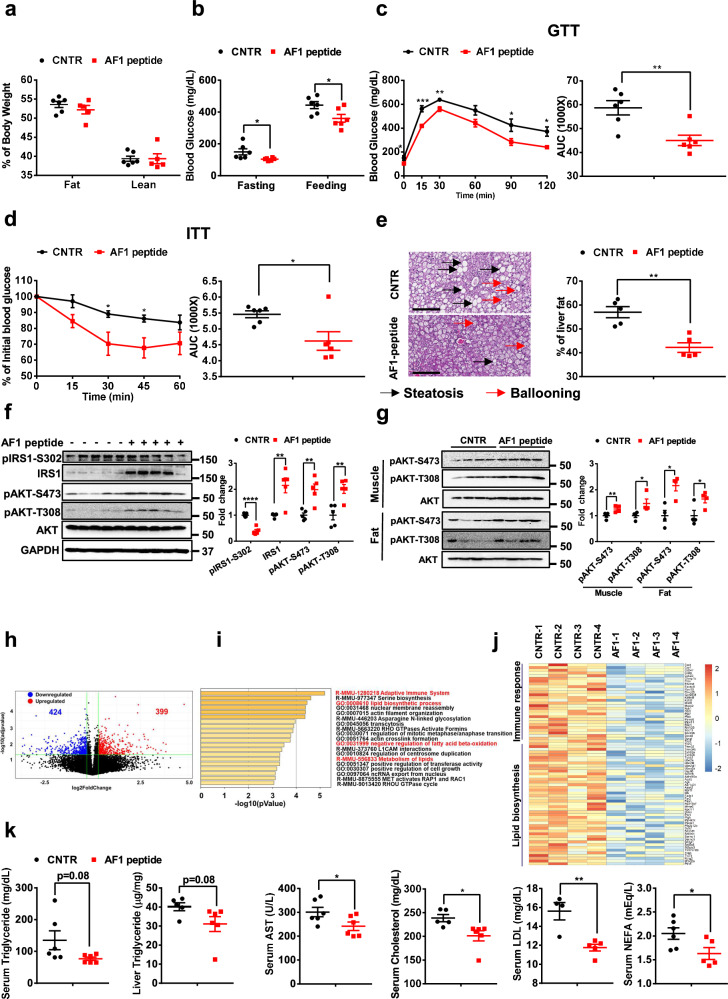
AF1 peptide improves glucose tolerance and insulin sensitivity in diabetic mice. **a** Body weight of db/db mice treated with control and AF1 peptide for 5 weeks, *n* = 5 (AF1 peptide) and 6 (control) mice/group. **b** Random feeding and 16 h fasting blood glucose in db/db mice treated with control and AF1 peptide for 5 weeks, *n* = 6 mice/group; fasting blood glucose, *P* = 0.0409; feeding blood glucose, *P* = 0.0317. **c** Glucose tolerance tests in db/db mice treated with control and AF1 peptide for 5 weeks, *n* = 6 mice/group; 0 min, *P* = 0.0409; 15 min, *P* = 0.0006; 30 min, *P* = 0.0032; 90 min, *P* = 0.0375; 120 min, *P* = 0.0104; AUC, *P* = 0.0042. **d** Insulin tolerance tests in db/db mice treated with control and AF1 peptide for 5 weeks, *n* = 6 mice/group; 30 min, *P* = 0.0328; 45 min, *P* = 0.0198, AUC, *P* = 0.0217. **e** H&E staining of livers from db/db mice treated with control and AF1 peptide. Scale: 200 µm. Liver fat content was calculated, *n* = 5 mice/group; *P* = 0.0013. Representative images were shown. **f** Liver insulin sensitivity in db/db mice treated with control and AF1 peptide, *n* = 5 mice/group; pIRS1-S302, *P* < 0.0001, IRS1, *P* = 0.0040, pAKT-S473, *P* = 0.0026, pAKT-T308, *P* = 0.0037. **g** Phosphorylation of AKT in epididymal fat and skeleton muscle from db/db mice treated with control and AF1 peptide, *n* = 4 mice/group; for fat, pAKT-S473, *P* = 0.0257, pAKT-T308, *P* = 0.0491, for muscle, pAKT-S473, *P* = 0.0069, pAKT-T308, *P* = 0.0450. **h** Volcano plots of differentially expressed genes (DEGs) in livers from db/db mice treated with control and AF1 peptide. Genes upregulated or downregulated by more than 1.3-fold are shown in red and blue, respectively. **i** KEGG pathway analysis of DEGs in livers from db/db mice treated with control and AF1 peptide. **j** Heatmap of representative DEGs in livers from db/db mice treated with control and AF1 peptide. **k** Liver triglyceride, serum triglyceride, AST, cholesterol, LDL, and NEFA levels in db/db mice treated with control and AF1 peptide, *n* = 4 (serum LDL of CNTR group), 5 (liver triglyceride and serum cholesterol of CNTR group as well as serum LDL and NEFA of AF1 peptide group), and 6 (serum triglyceride and AST of CNTR and AF1 peptide group, liver triglyceride and serum cholesterol of AF1 peptide group, and serum NEFAof CNTR group); serum AST, *P* = 0.0487, serum cholesterol, *P* = 0.0234, serum LDL, *P* = 0.0037, serum NEFA, *P* = 0.0411. Data are presented as mean ± SEM. *P < 0.05, **P < 0.01, ***P < 0.001, ****P < 0.0001, unpaired Two-tailed Student’s t test. CNTR: Control. Source data are provided as a Source Data file.

To examine the effect of the AF1 peptide on diet-induced glucose dysregulation, AF1 peptide (5 mg/kg body weight, twice per week) was intravenously administered to male diet-induced obesity (DIO) or db/db mice for 5 weeks. In db/db mice, AF1 peptide treatment had no significant effect on body weight and body composition (Fig. 7a and Fig. S7a). However, the db/db mice treated with AF1 peptide had a 35% decrease in overnight fasting blood glucose (CNTR: 150.2 ± 19.4 vs. AF1 peptide: 103.8 ± 3.6 mg/dL, *P* < 0.05) and a 22% reduction in random feeding blood glucose (CNTR: 443.7 ± 21.9 vs. AF1 peptide: 360.7 ± 25.1 mg/dL, *P* < 0.05, Fig. 7b). Blood glucose was significantly decreased by AF1 peptide in db/db mice upon glucose or insulin challenge (Fig. 7c, d). Both control and AF1 peptide-treated db/db mice showed hepatocellular ballooning in livers. AF1 peptide significantly decreased liver steatosis, indicated by decreases in liver steatosis structure and liver fat amount (*P* < 0.01; Fig. 7e and Fig. S7b). Hepatic insulin sensitivity was significantly increased by AF1 peptide treatment in db/db mice, indicated by a 61% decrease in pIRS1-S302 (*P* < 0.0001) as well as increases in IRS1 by 114%, pAKT-S473 by 98%, and pAKT-T308 by 101% (*P* < 0.01, Fig. 7f). In both skeletal muscle and epididymal white adipose tissue (eWAT), pAKT-S473 and pAKT-T308 were significantly increased by AF1 peptide treatment in db/db mice (Fig. 7g). Liver RNA-Seq analysis showed that 399 genes were upregulated and 424 genes downregulated by AF1 peptide treatment in db/db mice (Fig. 7h). Pathway analysis of differentially expressed genes showed that immune response, lipid biosynthesis, and lipid metabolism pathways were significantly attenuated by AF1 peptide treatment (Fig. 7i–j). AF1 peptide treated db/db mice showed a decreasing trend in both serum and liver triglycerides. Serum AST, cholesterol, LDL, HDL, and NEFA levels were significantly reduced in AF1 peptide treated db/db mice. AF1 peptide had a limited effect on serum insulin, ALP, and ALT levels (Fig. 7k, Fig. S7a).

In DIO mice, AF1 peptide treatment did not change body weight (Fig. S7c). However, AF1 peptide treated DIO mice had significant decreases in random feeding blood glucose by 12% (CNTR: 175.5 ± 5.3 vs. AF1 peptide: 154.2 ± 4.7 mg/dL, *P* < 0.05) and overnight fasting blood glucose by 20% (CNTR: 106.2 ± 6.9 vs. AF1 peptide: 85.4 ± 0.2 mg/mL, *P* < 0.05, Fig. S7d). Consistently, glucose tolerance and insulin sensitivity were significantly improved by AF1 peptide in DIO mice (Fig. S7e–h). In the liver of DIO mice, AF1 peptide ameliorated fat accumulation (Fig. S7i) and significantly decreased pIRS1-302 by 32% (*P* < 0.05) and increased IRS1, pAKT-S473, and pAKT-308 by 49%,100%, and 51%, respectively (*P* < 0.05, Fig. S7j, k). Gene expression analysis showed that AF1 peptide treatment significantly decreased mRNA expression of gluconeogenic gene (*Pck1*), and inflammatory cytokine (*Il6*) as well as increased mRNA expression of FAO gene (*Hadha*) in the liver of DIO mice (Fig. S7l). Serum insulin, AST, ALT, and ALP were not changed by AF1 peptide. Serum lipid profile was significantly improved by AF1 peptide treatment, indicated by decreases in cholesterol by 8% (*P* < 0.01), triglyceride by 21% (*P* < 0.05), and NEFA by 21% (*P* < 0.05). AF1 peptide treated DIO mice also showed decreases in both serum HDL (*P* < 0.05) and LDL (Fig. S4m, n). We further detected the effect of AF1 peptide in liver-specific IRS1 and IRS2 knockout (DKO) male mice. DKO male mice showed severe glucose intolerance and insulin resistance and AF1 peptide treatment significantly improved glucose homeostasis and insulin sensitivity in DKO male mice (Fig. S8a-c). In the liver, insulin-induced AKT phosphorylation was dramatically diminished in DKO male mice, which could not be rescued by AF1 peptide treatment (Fig. S8d). In the skeletal muscle and eWAT, DKO male mice showed a decrease in insulin-stimulated AKT phosphorylation, compared to control mice, which is partially improved by AF1 peptide treatment (Fig. S8e, f). These results indicate that AF1 peptide improves glucose homeostasis in diabetic mouse models.

Postmenopause in old women is highly associated with increased risk of diabetes. To further examine whether AF1 peptide improves glucose homeostasis in aged females, we treated 12-month-old female mice with 5 mg/kg body weight AF1 peptide via intravenous injection (twice per week) and found that AF1 peptide led to a nonsignificant increase in glucose tolerance and modest improvement in insulin sensitivity (Fig. S8g–i). These results indicate that AF1 peptide may improve glucose homeostasis in aging females, especially after menopause.

## Discussion

ERα is the major isoform of estrogen receptor in the regulation of energy balance and glucose homeostasis, which is mediated by a non-genomic pathway^7,8,11^. Our previous study showed that ERα increased insulin sensitivity by activating the AKT-FOXO1 pathway in response to E_2_^12^. In this study, we found that ERα enhanced insulin action in a ligand-independent manner. Mechanistically, ERα bound to IRS1 and increased its stability potentially through inhibiting IRS1-S302 phosphorylation and ubiquitin-induced degradation. We further found that the ERα 1-280 domain played an important role in ERα-stimulated insulin sensitivity. Finally, based on the feature of ERα 1-280 domain amino acid sequence, we designed a peptide that acts as an insulin sensitizer, increasing insulin sensitivity and improving glucose homeostasis in obese mice.

The incidence of diabetes in men is much higher than that in women^5,18^. After menopause, women gradually develop obesity and insulin resistance and are at high risk of type 2 diabetes, but hormone replacement therapy improves insulin sensitivity and glycemic control^19–21^. These results suggest that E_2_ signaling pathway plays an important role in the regulation of insulin sensitivity and glucose homeostasis. ERα plays a dominant role in mediating the effect of E_2_ on the regulation of energy balance and glucose homeostasis^8^. Consistently, we found that hepatic ERα, but not ERβ, deficiency impaired glucose tolerance and insulin sensitivity. E_2_-ERα signaling exerts its function through both genomic and non-genomic mechanisms^10^. A previous study showed that recovery of ERα non-genomic function rescues ERα deficiency-induced dysregulation of energy metabolism and glucose homeostasis^11^, suggesting that ERα non-genomic pathway mainly mediates its role in metabolic regulation. E_2_-ERα signaling stimulates PI3K activity, thereby increasing insulin sensitivity and suppressing HGP^12,13^. Here we provided evidence that E_2_-ERα activates insulin signaling pathway independent of IRS1 and IRS2. Compared to control mice, DKO female mice showed significantly impaired glucose tolerance and insulin sensitivity upon glucose and insulin challenge. However, glucose handling and insulin sensitivity in DKO female mice were significantly improved, as compared to DKO male mice; such effect is abolished in TKO female mice. Therefore, in addition to the classic insulin signaling pathway (insulin receptor-IRS-PI3K-AKT), E_2_-ERα-PI3K-AKT signaling pathway enhances insulin sensitivity independent of IRS. Moreover, we found that hepatic ERα deletion impaired insulin sensitivity and glucose homeostasis in both male and OVX female mice, indicating that ERα also regulates glucose metabolism in a ligand-independent manner. In DKO male mice, hepatic ERα knockout did not further aggravate glucose intolerance and insulin resistance, which suggests that IRS1 and 2 are required for ERα ligand-independent effect. Therefore, ERα controls insulin sensitivity in two major non-genomic pathways: the ligand-dependent E_2_-ERα-PI3K-AKT pathway and the ligand-independent ERα-IRS-PI3K-AKT pathway. Estrogen-ERα signaling plays an important role in control of energy homeostasis^4^. In HFD-fed female but not male mice, hepatic ERα deletion led to a significant increase in body weight and fat mass, which may be attributed to estrogen-mediated ERα signaling pathway. However, further study is warranted to investigate the underlying mechanism by which hepatic estrogen-ERα signaling pathway regulates energy homeostasis.

IRS1 and IRS2 are required for insulin-induced carbohydrate and lipid metabolism^15,22^. A previous study reported that ERα regulated endogenous IRS1 and IRS2 degradation in breast cancer cells^16^, suggesting that IRS proteins may be the target of ERα in regulating glucose homeostasis and insulin sensitivity in mice. In this study, we found that ERα interacted with both IRS1 and IRS2 but only regulated IRS1 protein abundance, with a limited effect on IRS2 protein levels in mouse primary hepatocytes. The mRNA expression levels of *Irs1* and *Irs2* were not affected in ERα deficient hepatocytes, suggesting that ERα controls IRS expression through a post-translational modification. We found that ERα protected against ubiquitination-mediated IRS1 degradation but not IRS2. IRS1 and IRS2 contain a highly similar amino-terminal pleskstrin homology (PH) and phosphostyrosin-binding domain (PTB), whereas the tail region of IRS1 and IRS2 are poorly conserved^14^. A previous study showed that E3 ubiquitin ligase MG53 promoted IRS1 ubiquitination and degradation but had no effect on IRS2^23^. Therefore, we assume that the distinct effects of ERα on IRS1 and IRS2 protein expression may be attributed to the differential regulation of E3 ubiquitin ligase in IRS1 and IRS2. Our data showed that E_2_ treatment had no effect on the interaction between ERα and IRS1, suggesting that ERα interacts with IRS1 in a ligand-independent manner. Considering E_2_ stimulates ERα nuclear translocation and IRS1 exists in both cytosol and nucleus^24^, it is possible that ERα interacts with IRS1 in both cytosol and nucleus. A previous study showed that nuclear ERα but not membrane ERα gain-of-function rescued ERα deficiency-induced glucose intolerance and insulin resistance^25^. It would be interesting to further investigate the physiological significance of nuclear IRS1-ERα interaction in future study.

Multiple serine phosphorylation sites of IRS1 have been indicated to be correlated to insulin resistance, including S302, S307, S636/639, and S1101^14,26–29^. Here, we showed that ERα interacted with the IRS1 PTB (100-300 amino acids) and inhibited IRS1-S302 phosphorylation, which prevents IRS1 degradation. In diabetic mouse liver, IRS1-S302 phosphorylation was significantly increased and suppression of IRS1-302 phosphorylation blocked JNK1-induced insulin resistance^27^. IRS1-S302 phosphorylation, mainly stimulated by mTOR/S6K pathway, impaired its binding to insulin receptor and disrupted insulin signaling^28^. Alanine mutations of IRS1 at S302, S307, and S612 protected against fat-induced insulin resistance in the mouse skeletal muscle^26^. These results indicate that IRS1-302 phosphorylation potentially plays an important role in the development of T2DM. However, a previous study showed that IRS1-S302A knock-in mice had normal glucose homeostasis and muscle insulin sensitivity under physiological condition^30^. Considering the potential role of IRS1-S302 phosphorylation in insulin resistance, the phenotype of IRS1-S302A knock-in mice under pathological condition should be evaluated. In this study, we observed that ERα enhanced IRS1 protein abundance through inhibiting IRS1 ubiquitination, which is potentially mediated by IRS1-S302 phosphorylation. However, further experiments are warranted to detect the role of pIRS1-S302 in ERα-regulated hepatic insulin sensitivity in the future.

Hepatic ERα expression levels were decreased in both mice and humans with diabetes. Interestingly, we found that hepatic ERα expression levels were only significantly decreased in poorly controlled diabetic patients, which may be attributed to hyperinsulinemia-induced reprogramming of liver transcriptomics. These results indicate that hepatic ERα may be a key checkpoint for the progression of diabetes. ERα consists of the N-terminal activation function-1 (AF1) domain, the DNA binding domain (DBD), and the C-terminal activation function-2 (AF2) domain^31^. The AF1 domain is constitutively active, whereas the AF2 domain is located within the hormone binding domain and is more ligand-regulatable. Both the AF1 and AF2 domains contribute to the transcriptional activity of target genes^32^. The AF2 domain, but not AF1 domain, is required by estrogen-mediated prevention of obesity and insulin resistance^33^. Selective activation of the AF1 domain by tamoxifen protects mice from diet-induced obesity, steatosis, and insulin resistance^34^; this result suggests that AF1 domain plays an important role in the regulation of metabolic homeostasis in a ligand-dependent manner. In this study, we identified that AF1 domain mediated the ligand-independent effect of ERα on insulin sensitivity. We found that liver ERα deletion resulted in hepatic insulin resistance in male, female, and OVX female mice, which is consistent with a previous study^35^. Overexpression of ERα in the liver of male mice increased hepatic insulin sensitivity, suggesting that ERα potentially regulates insulin sensitivity in a ligand-independent manner. The AF1-DBD domain, rather than DBD-AF2 domain, copied the effect of ERα on hepatic insulin action in vitro and in vivo. Recombinant AF1 domains can be made to fold by conjunction with the DBD domain, thereby affecting AF1 domain function and promoting its interaction with other factors in a ligand-independent manner^31,36^. The conjunction of AF1 with the DBD domain potentially changes AF1 function state and renders its interaction with IRS1, thus increasing IRS1 protein stability. Therefore, the ligand-independent effect of ERα on insulin sensitivity is mainly mediated through the AF1 domain.

Peptides have gained an increased interest in pharmaceutical research due to their high specificity, efficiency, and safety^37^. A number of peptides have been designed to prevent the development of obesity, including glucagon-like peptide-1 (GLP-1), natriuretic peptide, and defensin-derived peptide^38–40^. In this study, we designed an insulin sensitizing peptide based on the interaction between IRS1 and ERα. We found that N-terminal of AF1-DBD domain (1-60 aa) exhibited a high interaction score with IRS1 and enhanced insulin sensitivity. We narrowed down the AF1-DBD 1-60 domain and screened out 34-aa-peptide as a potential insulin sensitizer that interacts with IRS1 and increases insulin sensitivity. HIV TAT-derived peptide, a small basic peptide, can deliver target proteins into living cells^41,42^. Thus, the conjunction of TAT-derived peptide to the N-terminal of 34-aa-peptide (AF1 peptide) was prepared. Our results showed that the AF1 peptide interacted with the IRS1 1-300 domain, increased IRS1 protein stability, and enhanced insulin sensitivity. However, the AF1 peptide did not completely normalize ERα deficiency-induced insulin resistance in primary hepatocytes, suggesting that ERα also mediates hepatic insulin sensitivity through other mechanisms. In the obese mouse model, the AF1 peptide improved glucose tolerance, insulin sensitivity, and serum lipid profiles without affecting body weight. Although we found that the AF1 peptide increased IRS1 protein abundance in the obese mouse liver, we cannot rule out other potential targets regulated by AF1 peptide to mediate its beneficial effects on glucose and lipid homeostasis. Previous studies showed that ERα regulated glucose homeostasis and insulin sensitivity in skeletal muscle and adipose tissue^8,9,43,44^. In skeletal muscle, global ERα knockout significantly decreased insulin-mediated glucose uptake in female mice^8^. Adipose tissue ERα deletion resulted in enlarged adipocytes as well as increased adipose tissue inflammation and fibrosis in both male and female mice. Glucose tolerance and insulin sensitivity were largely impaired in the adipose tissue knockout male mice^44^. Indeed, we found that AF1 peptide significantly increased insulin sensitivity in both skeletal muscle and adipose tissue (eWAT) of db/db mice. In the DKO mouse model, the AF1 peptide had no effect on hepatic insulin sensitivity, indicating that the effect AF1 peptide requires IRS proteins. However, glucose homeostasis was still improved in AF1 peptide treated DKO mice, which is partially attributed to the improvement of insulin sensitivity in adipose tissue and skeletal muscle. These results indicate that AF1 peptide potentially targets adipose tissue and skeletal muscle, thereby regulating insulin sensitivity and glucose homeostasis. Moreover, we found that AF1 peptide improved insulin sensitivity and glucose homeostasis in aging female mice (12-month-old); this result suggests that AF1 peptide potentially improves menopause-induced dysregulation of glucose homeostasis. However, the effect of AF1 peptide in aged female mice (18-month-old) needs to be further investigated. Overall, the AF1 peptide is a potentially effective insulin sensitizer to treat T2DM. Collectively, we found that ERα enhanced insulin sensitivity in a ligand-independent manner. Mechanistically, ERα interacted with IRS1 (100-300 domain), thereby increasing IRS1 protein stability. Based on ERα function, we designed an AF1 peptide that potentially acts as an insulin sensitizer to improve glucose homeostasis and lipid profile in diabetic mouse models.

There are several limitations in the current study that warrant future investigation. Our study showed that ERα increased IRS1 protein stability potentially through phosphorylation of IRS1-S302. Whether IRS1-S302 phosphorylation is sufficient to mediate the effect of ERα on insulin signaling needs to be further investigated. Our study showed that AF1 peptide is a promising insulin sensitizer. However, we do not show whether AF1 peptide improves liver microenvironment during obesity, especially the effect of AF1 peptide on hepatic stellate cells and Kupffer cells during obesity. In addition, our study only detected the effect of AF1 peptide in obese mouse model. The effect of AF1 peptide on insulin sensitivity in other animal models should be further validated.

## Methods

### Animal studies

All animal experiments were performed following procedures approved by the Texas A&M University Institutional Animal Care and Use Committee. Mice are housed under controlled environmental conditions, with a temperature of 22-24 C°, humidity maintained at 55% ± 5%, and a 12 h light/12 h dark cycle with a standard chow diet (58% calories from carbohydrate, 18% from fat, and 24% from protein, 3.1 kcal/g, with a reduced phytoestrogen content; 2018, Teklad Diet) *ad libitum*. Liver-specific ERα (*ERα*^LivKO^) or ERβ (*ERβ*^LivKO^) knockout mice were generated by crossing ERα or ERβ flox mice (gift from Dr. Yong Xu, Baylor College of Medicine) with Albumin-Cre mice purchased from The Jackson Laboratory (Strain #003574), respectively. Liver IRS1 and IRS2 double knockout (DKO) mice were generated by breeding IRS1 and IRS2 flox (IRS1^L/L^::IRS2 ^L/L^) mice with Albumin-Cre mice^15^. Liver IRS1, IRS2, and ERα triple knockout (TKO) mice were generated by crossing DKO mice with ERα^LivKO^ mice. The mice at the age of 8-12-week-old were used in the experiments. All mice were on C57B/6 J background. Db/db mice were purchased from The Jackson Laboratory (Strain #000697). Female mice at age of 2-month-old underwent a bilateral ovariectomy (OVX) surgery^12^; these mice were used to perform experiments 4 weeks after OVX surgery. All the mice experiments were performed with age- and gender- matched mice with their health status examined daily by the investigators and technicians from Texas A&M University LARR Animal Facility. Littermate controls were used if possible. Euthanasia of mice was performed using carbon dioxide inhalation. Carbon dioxide was delivered into the mouse cage at a rate of 2 L/min until the cessation of respiration and then cervical dislocation was performed to guarantee death.

### Obese mouse models and AF1 peptide treatment

For the high-fat diet (HFD)-induced obese mouse model, the 6-8-week-old male mice were fed with HFD (HFD; 60% kcal from fat, D12492; Research diet, New Brunswick, NJ) for 11 weeks. Then mice received 5 mg/kg body weight control or AF1 peptide through retro orbital injection (twice per week; 5 weeks). For genetic obesity mouse model, the 8-week-old db/db mice were administered with 5 mg/kg body weight control or AF1 peptide through retro orbital injection (twice per week; 5 weeks). Glucose tolerance and insulin tolerance tests were performed in the fourth and fifth week after treatment, respectively. Body composition was measured using EchoMRI^TM^ one day before sacrificing the mice.

### Ovariectomy and estrogen replacement

Female mice were randomly assigned to experimental groups. Bilateral ovariectomy surgery was performed in female mice^12^. Placebo or 17β-estradiol (E_2_) pellet (0.05 mg/pellet, 60-day release; Innovative Research of America, Sarasota, FL) was subcutaneously implanted into ovariectomized female mice^45^. Female mice were subjected to E_2_ or placebo pellet implantation at same time of bilateral ovariectomy surgery.

### Primary hepatocytes isolation and cell culture

Primary hepatocytes were isolated, as previously described^46^. Male mice at age of 8-12 weeks were infused with a perfusion buffer A (calcium and magnesium-free HBSS supplemented with 10 mM HEPES, 50 mM EGTA, 1 mM glucose, and 1% penicillin-streptomycin, pH 7.4) through portal vein for 6-8 min. Then perfusion buffer A was changed to buffer B (calcium and magnesium -free HBSS supplemented with 10 mM HEPES, 1 mM CaCl_2_, 1 mM glucose, 1% penicillin-streptomycin, and 0.5 mg/mL collagenase II, pH 7.4). When showed signs of cracking at the surface, the liver was transferred into the ice-cold serum-free DMEM. Liver cells were suspended and passed through a 70 µm cell strainer, followed by centrifuge at 320 x g for 2 min. Pellets were resuspended with Percoll at final concentration of 36% and centrifuged at 360 x g for 6 min. The hepatocyte pellet was washed and suspended with DMEM supplemented with 10% FBS and 1% penicillin-streptomycin in collagen-coated plates. The HepG2 cell line was purchased from Sigma (Cat# 85011430) and cultured with DMEM medium supplemented with 10% FBS and 1% penicillin-streptomycin in collagen-coated plates. The human embryo kidney (HEK) 293 cell line was purchased from Abcam (Cat# ab259776) and cultured with DMEM medium supplemented with 10% FBS and 1% penicillin-streptomycin. FBS-free DMEM medium was used to culture cells during the experiments.

### Glucose and insulin tolerance tests

For glucose tolerance tests, mice were fasted for 16 h and intraperitoneally (i.p.) injected with dextrose (2 g/kg body weight for mice fed with regular diet; 1 g/kg body weight for db/db mice or mice fed with HFD). For insulin tolerance tests, mice were fasted for 6 h and i.p. injected with insulin solution (1 U/kg body weight for male mice; 0.75 U/kg body weight for female mice). Blood glucose levels were measured using glucometer at indicated time points.

### Hepatic glucose production

The primary mouse hepatocytes were isolated from 8-12-week-old male mice and glucose production was measured. Cells were resuspended in DMEM medium (2% FBS and 1% penicillin-streptomycin) for 3 h and cultured in HGP buffer (118 mM NaCl, 2.5 mM CaCl2, 4.8 mM KCl, 25 mM NaHCO3, 1.1 mM KH2PO4, 1.2 mM MgSO4, 10 mM ZnSO4, 0.6% BSA, 10 mM HEPES, 10 mM sodium DL-lactate, and 5 mM pyruvate, pH 7.4). Then cells were pretreated with 50 nM glucagon for 30 min, followed by 10 nM insulin treatment for 3 h, and glucose level in the medium was measured using Amplex Red Glucose Assay^47^. The results were normalized by cell protein abundance.

### RNA extraction and quantitative real-time PCR

Total RNA was extracted from livers or primary hepatocytes using TRIzol (Invitrogen, Thermo Fisher Scientific), purified with chloroform, 80% ethanol, isopropanol, and RNA column, eluted with RNase free water, and reverse transcribed into cDNA using iScript Reverse Transcription Supermix (Bio-Rad, Hercules, CA). Quantitative real-time PCR (qPCR) was conducted using using SsoAdvanced Universal SYBR Green Supermix (Bio-Rad, Hercules, CA) on CFX384 real-time PCR detection system (Bio-Rad, Hercules, CA). Q-PCR started with 95 °C incubation for 3 min, followed by 39 cycles of 95 °C for 5 s and 60 °C for 30 s. The threshold cycle (CT) values were obtained and used to calculate the relative transcriptional level of target genes by 2^-ΔΔCT^ method with Cyclophillin (*Cyp*) as an internal control. Statistical analysis was performed using 2^-ΔΔCT^. The primers are listed in Supplementary Table 1.

### Immunofluorescence staining

Cells were fixed with 4% formaldehyde solution, followed by incubation with blocking buffer (1X PBS/ 5% normal serum/ 0.3% Triton X-100) for 1 h. After rinsing three times using PBS, cells were incubated with primary antibodies (anti-Flag antibody, Cell Signaling Technology, Cat# 8146, Dilution: 1:800; anti-HA antibody, Cell Signaling Technology, Cat# 3724, Dilution: 1:800) overnight at 4 °C. Cells were washed with PBS and incubated with secondary antibodies (anti-rabbit IgG Alexa Flour 594 Conjugate, Cell Signaling Technology, Cat# 8889, Dilution: 1:500 and anti-mouse IgG Alexa Flour 488 Conjugate, Cell Signaling Technology, Cat# 4408, Dilution: 1:500) at room temperature 1–2 h. Cells were covered with DAPI-contained mounting medium. Fluorescent signal was observed using Confocal microscopy (Leica).

### Hematoxylin and eosin staining and Oil Red O staining

Livers were embedded into paraffin after being fixed by PFA, and then cut into 5 µm sections. After deparaffinization and hydration, liver sections were stained with hematoxylin for 5 min. After differentiation by 1% acid alcohol, liver sections were stained with 1% eosin for 3 min. Finally, liver sections were dehydrated and mounted with mounting media. For Oil Red O staining, liver frozen sections were prepared and fixed in 10% neutral buffered formalin for 10 min after air dry for 1 h. Liver sections were stained in Oil Red O working solution for 15 min after a quick dip in 60% isopropanol. Liver sections were then stained with hematoxylin for 3 min and covered with aqueous mounting gel. Liver sections were observed using slide scanner (Leica).

### Adenovirus infection

For ERα and ERα 1-280 overexpression mouse model, 8-12-week-old male mice were administered with adenovirus-Flag-ERα and Flag-ERα 1-280 (1 × 10^10^ genome copies/mouse) through retro-orbital injection. Two weeks after adenovirus injection, glucose and insulin tolerance tests were performed. For ERα overexpression in primary hepatocytes, 100 MOI adenovirus ERα was used to infect primary mouse hepatocytes for 24 h, followed by 100 nM insulin treatment for 1 h. Cell proteins were collected using lysis buffer for Western blot analysis.

### Co-immunoprecipitation and western blotting analysis

Flag-labeled IRS or truncated IRS proteins were co-transfected into HEK293 cells with HA-labeled ERα and its truncated proteins for 30 h using lipofectamine 3000 (Thermo Fisher), followed by 10 µM MG132 treatment for 1 h. Cell proteins were extracted using TNE buffer and incubated with primary antibody (anti-Flag antibody, Cell Signaling Technology, Cat# 14793, Dilution: 1:50) overnight at 4 °C. Flag-labeled proteins were immunoprecipitated using Dynabeads Protein G. HA-labeled proteins were detected using Western blotting. For Western blotting, tissues or cells were homogenized in a RIPA buffer supplemented with protease and phosphatase inhibitors. Equal amount of proteins were analyzed using Wester blotting assays. The proteins were separated with Sodium dodecyl-sulfate polyacrylamide gel electrophoresis (SDS-PAGE) system. The gel was transferred onto a 0.45 µm PVDF membrane at 100 V for 2 h at 4 °C, followed by blocking in 5% BSA for 1 h at room temperature. The membrane was then incubated with primary antibodies including anti-IRS1 antibody (Cell Signaling Technology, Cat# 2390, Dilution: 1:1000), anti-IRS2 antibody (Cell Signaling Technology, Cat# 4502, Dilution: 1:1000), anti-pAKT-S473 antibody (Cell Signaling Technology, Cat# 9271, Dilution: 1:1000), anti-pAKT-T308 antibody (Cell Signaling Technology, Cat# 13038, Dilution: 1:1000), anti-AKT antibody (Cell Signaling Technology, Cat# 4691, Dilution: 1:1000), anti-ERα antibody (Cell Signaling Technology, Cat# 13258, Dilution: 1:1000), anti-pIRS1-S302 antibody (Cell Signaling Technology, Cat# 2384, Dilution: 1:1000), anti-pIRS1-S307 antibody (Cell Signaling Technology, Cat# 2381, Dilution: 1:1000), anti-pIRS1-S636/639 antibody (Cell Signaling Technology, Cat# 2388, Dilution: 1:1000), anti-pIRS1-S1101 antibody (Cell Signaling Technology, Cat# 2385, Dilution: 1:1000), anti-p85 antibody (Cell Signaling Technology, Cat# 4292, Dilution: 1:1000), anti-Ubiquitin antibody (Cell Signaling Technology, Cat# 58395, Dilution: 1:1000), anti-HA antibody (Cell Signaling Technology, Cat# 2367, Dilution: 1:1000), anti-Flag antibody (Cell Signaling Technology, Cat# 8146, Dilution: 1:1000), and anti-GAPDH antibody (Cell Signaling Technology, Cat# 5174, Dilution: 1:3000) at 4 °C overnight, followed by secondary antibody incubation (anti-rabbit IgG HRP-linked, Cell Signaling Technology, Cat# 7074, Dilution: 1:3000 and anti-mouse IgG HRP-linked, Cell Signaling Technology, Cat# 7076, Dilution: 1:3000) for 1 h at room temperature. Protein bands were detected using a ChemiDoc image system. Protein signals were quantified using ImageJ 1.53 K (National Institutes of Health, Bethesda, Maryland).

### Protein interaction prediction

Interaction between IRS1 protein and ERα protein domains was predicted using ProPrInt (URL: https://webs.iiitd.edu.in/raghava/proprint/submit.html).

### AF1 peptide and IRS1 protein interaction

HEK293 cells were transfected with Flag-IRS1 or Flag-IRS1 truncated domains using lipofectamine 3000 for 30 h, followed by treatment of 10 µM control and AF1 peptides labeled with FITC fluorescence for 3 h. After 10 µM MG132 treatment for 1 h, cell protein was extracted, and Flag-labeled proteins were immunoprecipitated. The intensity of control and AF1 peptide was calculated through measuring FITC fluorescence signal with multiplex reader. The results were normalized using IgG group.

### Serum lipid profile analysis

Serum triglyceride, AST, ALT, cholesterol, LDL, HDL, NEFA, and ALP were measured using DxC 700 AU Chemistry Analyzer (Beckman Coulter).

### Serum insulin and liver triglyceride analysis

Serum insulin and triglyceride levels were measured by using mouse insulin ELISA kit (Mercodia) and triglyceride assay kit (Abcam), respectively.

### RNA-Seq analysis

Total RNA was isolated from frozen liver tissues of db/db mice treated with control and AF1 peptide (*n* = 4 per group). The RNA-Seq was performed at TIGSS molecular genomics core in Texas A&M University. Analysis of RNA -seq data was performed at Texas A&M University high performance research computing institute. Briefly, reads were trimmed for adapters and low-quality bases with TrimGalore v0.6.7 with default settings. Trimmed reads were aligned to the domestic mouse genome using HiSat2.2.1. Alignment files were sorted and indexed with SAMtools v1.17. Read counts were generated with the R package GenomicAlignments v11.34.1. The significantly expressed genes were determined by p value less than 0.05. Function analysis of differentially expressed genes was performed using Metascape.

### Human liver gene expression analysis

The human liver gene profile dataset was downloaded from NCBI (GSE15653)^17^. The differential gene expression was analyzed using GEO2R. Pearson correlation coefficient between liver *ERα* gene expression level and fasting blood glucose, HbA1C, or HOMA-IR was calculated.

### Statistical analysis

Results were shown as mean ± SEM, with n representing the number of biological replicates. GraphPad Prism 6.01 was used for statistical analysis and graphs. No samples or data were excluded from the study for statistical purposes. Each in vitro experiment was independently performed twice or three times to ensure reproducibility. Animals were randomly assigned into control and treatment groups in all studies. The correlation coefficient and its significance between two independent variables were evaluated by Person’s correlation test. To calculate p value in the studies with two groups, unpaired two-tailed Student’s t tests were performed for statistical analysis. To determine the different significance among multiple groups, one-way ANOVA or two-way ANOVA with Tukey’s multiple comparisons test was used. p < 0.05 was considered to be statistically significant.

### Reporting summary

Further information on research design is available in the Nature Portfolio Reporting Summary linked to this article.

### Supplementary information

Supplementary information

Peer Review File

Reporting Summary

### Source data

Source Data

## Data Availability

New generated data and source data have been deposited in Figshare. Publicly available Affymetrix human genome array data from healthy and diabetic individuals were downloaded on the Gene Expression Omnibus under the number GSE15653. The bulk RNA-seq data generated in this study have been deposited in the NCBI database under accession code GSE262841. Source data, uncropped blots, and raw counts for bulk RNA-seq for livers of db/db mice treated with control and AF1 peptide are provided with this paper and under the 10.6084/m9.figshare.25153235. Source data are provided with this paper.

## References

[1] Chatterjee S, Khunti K, Davies MJ (2017). Type 2 diabetes. The lancet.

[2] Muoio DM, Newgard CB (2008). Molecular and metabolic mechanisms of insulin resistance and β-cell failure in type 2 diabetes. Nat. Rev. Mol. Cell Biol..

[3] DeFronzo RA (2015). Type 2 diabetes mellitus. Nat. Rev. Dis. Primers.

[4] Mauvais-Jarvis F, Clegg DJ, Hevener AL (2013). The role of estrogens in control of energy balance and glucose homeostasis. Endocr. Rev..

[5] Zhou B (2016). Worldwide trends in diabetes since 1980: a pooled analysis of 751 population-based studies with 4· 4 million participants. Lancet.

[6] Peters SA, Muntner P, Woodward M (2019). Sex differences in the prevalence of, and trends in, cardiovascular risk factors, treatment, and control in the United States, 2001 to 2016. Circulation.

[7] Yang W., Guo J., Guo S. Insulin Resistance in Obesity. In: *Metabolic Syndrome: A Comprehensive Textbook*). Springer (2024).

[8] Bryzgalova G (2006). Evidence that oestrogen receptor-α plays an important role in the regulation of glucose homeostasis in mice: insulin sensitivity in the liver. Diabetologia.

[9] Heine P, Taylor J, Iwamoto G, Lubahn D, Cooke P (2000). Increased adipose tissue in male and female estrogen receptor-α knockout mice. Proc. Natl. Acad. Sci..

[10] Simoncini T, Genazzani AR (2003). Non-genomic actions of sex steroid hormones. Eur. J. Endocrinol..

[11] Park CJ (2011). Genetic rescue of nonclassical ERα signaling normalizes energy balance in obese Erα-null mutant mice. J. Clin. Investig..

[12] Yan H (2019). Estrogen improves insulin sensitivity and suppresses gluconeogenesis via the transcription factor Foxo1. Diabetes.

[13] Simoncini T (2000). Interaction of oestrogen receptor with the regulatory subunit of phosphatidylinositol-3-OH kinase. Nature.

[14] Copps K, White M (2012). Regulation of insulin sensitivity by serine/threonine phosphorylation of insulin receptor substrate proteins IRS1 and IRS2. Diabetologia.

[15] Dong XC (2008). Inactivation of hepatic Foxo1 by insulin signaling is required for adaptive nutrient homeostasis and endocrine growth regulation. Cell Metab..

[16] Morelli C, Garofalo C, Bartucci M, Surmacz E (2003). Estrogen receptor-α regulates the degradation of insulin receptor substrates 1 and 2 in breast cancer cells. Oncogene.

[17] Pihlajamaki J (2009). Thyroid hormone-related regulation of gene expression in human fatty liver. J. Clin. Endocrinol. Metab..

[18] Danaei G (2011). National, regional, and global trends in fasting plasma glucose and diabetes prevalence since 1980: systematic analysis of health examination surveys and epidemiological studies with 370 country-years and 2· 7 million participants. Lancet.

[19] Louet J-F, LeMay C, Mauvais-Jarvis F (2004). Antidiabetic actions of estrogen: insight from human and genetic mouse models. Curr. Atherosclerosis Rep..

[20] Espeland MA (1998). Effect of postmenopausal hormone therapy on glucose and insulin concentrations. Diabete Care.

[21] Salpeter S (2006). Meta‐analysis: effect of hormone‐replacement therapy on components of the metabolic syndrome in postmenopausal women. Diabetes Obesity Metab..

[22] Michael MD (2000). Loss of insulin signaling in hepatocytes leads to severe insulin resistance and progressive hepatic dysfunction. Mol. Cell.

[23] Song R (2013). Central role of E3 ubiquitin ligase MG53 in insulin resistance and metabolic disorders. Nature.

[24] Prisco M (2002). Nuclear translocation of insulin receptor substrate-1 by the simian virus 40 T antigen and the activated type 1 insulin-like growth factor receptor. J. Biol. Chem..

[25] Allard C (2019). Loss of nuclear and membrane estrogen receptor-α differentially impairs insulin secretion and action in male and female mice. Diabetes.

[26] Morino K (2008). Muscle-specific IRS-1 Ser→ Ala transgenic mice are protected from fat-induced insulin resistance in skeletal muscle. Diabetes.

[27] Werner ED, Lee J, Hansen L, Yuan M, Shoelson SE (2004). Insulin resistance due to phosphorylation of insulin receptor substrate-1 at serine 302. J. Biol. Chem..

[28] Harrington LS (2004). The TSC1-2 tumor suppressor controls insulin–PI3K signaling via regulation of IRS proteins. J. Cell Biol..

[29] Li Y (2004). Protein kinase C θ inhibits insulin signaling by phosphorylating IRS1 at Ser1101. J. Biol. Chem..

[30] Copps KD, Hançer NJ, Qiu W, White MF (2016). Serine 302 phosphorylation of mouse insulin receptor substrate 1 (IRS1) is dispensable for normal insulin signaling and feedback regulation by hepatic S6 kinase. J. Biol. Chem..

[31] Ascenzi P, Bocedi A, Marino M (2006). Structure–function relationship of estrogen receptor α and β: impact on human health. Mol. Aspects Med..

[32] Smith CL, O’malley BW (2004). Coregulator function: a key to understanding tissue specificity of selective receptor modulators. Endocr. Rev..

[33] Handgraaf S (2013). Prevention of obesity and insulin resistance by estrogens requires ERα activation function-2 (ERαAF-2), whereas ERαAF-1 is dispensable. Diabetes.

[34] Guillaume M (2017). Selective activation of estrogen receptor α activation function-1 is sufficient to prevent obesity, steatosis, and insulin resistance in mouse. Am. J. Pathol..

[35] Zhu L, Martinez MN, Emfinger CH, Palmisano BT, Stafford JM (2014). Estrogen signaling prevents diet-induced hepatic insulin resistance in male mice with obesity. Am. J. Physiol.-Endocrinol. Metab..

[36] Kumar R, Thompson EB (2003). Transactivation functions of the N-terminal domains of nuclear hormone receptors: Protein folding and coactivator interactions. Mol. Endocrinol..

[37] Fosgerau K, Hoffmann T (2015). Peptide therapeutics: current status and future directions. Drug Discov. Today.

[38] Bordicchia M (2012). Cardiac natriuretic peptides act via p38 MAPK to induce the brown fat thermogenic program in mouse and human adipocytes. J. Clin. Investig..

[39] Xu F (2016). GLP-1 receptor agonist promotes brown remodelling in mouse white adipose tissue through SIRT1. Diabetologia.

[40] Li Z (2023). A novel peptide protects against diet-induced obesity by suppressing appetite and modulating the gut microbiota. Gut.

[41] Fawell S (1994). Tat-mediated delivery of heterologous proteins into cells. Proc. Natl. Acad. Sci..

[42] Brooks H, Lebleu B, Vivès E (2005). Tat peptide-mediated cellular delivery: back to basics. Adv. Drug Del. Rev..

[43] Galluzzo P (2009). 17β-Estradiol regulates the first steps of skeletal muscle cell differentiation via ER-α-mediated signals. Am. J. Physiol.-Cell Physiol..

[44] Davis KE (2013). The sexually dimorphic role of adipose and adipocyte estrogen receptors in modulating adipose tissue expansion, inflammation, and fibrosis. Mol. Metab..

[45] Yan H (2022). Estrogen protects cardiac function and energy metabolism in dilated cardiomyopathy induced by loss of cardiac IRS1 and IRS2. Circul.: Heart Failure.

[46] Zhang K (2012). Hepatic suppression of Foxo1 and Foxo3 causes hypoglycemia and hyperlipidemia in mice. Endocrinology.

[47] Yang W (2023). Hepatic p38α MAPK controls gluconeogenesis via FOXO1 phosphorylation at S273 during glucagon signalling in mice. Diabetologia.

