# Supplementary material for: An estrogen receptor α-derived peptide improves glucose homeostasis during obesity
Source: Nat Commun. 2024 Apr 22;15:3410. doi: 10.1038/s41467-024-47687-6 (PMC11035554; doi:10.1038/s41467-024-47687-6)
Supplement: Supplementary file 1 — Supplementary information [file 41467_2024_47687_MOESM1_ESM.pdf]

Supplementary Information

An estrogen receptor α-derived peptide improves glucose homeostasis during obesity

Wanbao Yang, Wen Jiang, Wang Liao, Hui Yan, Weiqi Ai, Quan Pan, Wesley A. Brashear, Yong Xu, Ling He, Shaodong Guo

Figure S1

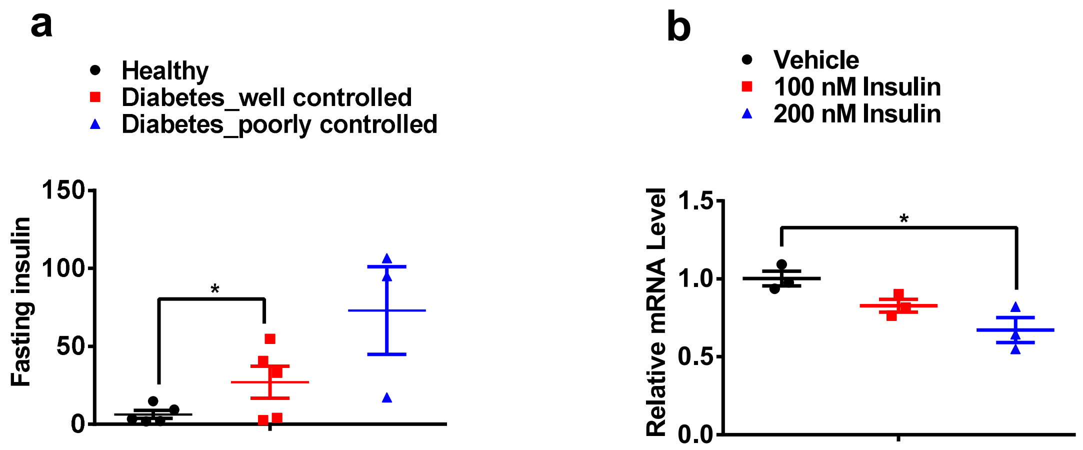

Fig. S1 Hyperinsulinemia decreases *ERα* mRNA expression in mouse primary hepatocytes. a Fasting insulin levels in healthy and diabetic patients, $n = 3$-5; $P = 0.0154$. **b** The mRNA expression levels of *ERα* in mouse primary hepatocytes treated with 100 nM and 200 nM insulin for 12 h, $n = 3$ independent cells; $P = 0.0169$. Data are presented as mean ± SEM. *$P < 0.05$, One-way ANOVA with Tukey's multiple comparisons test. Source data are provided as a source data file.

Figure S2

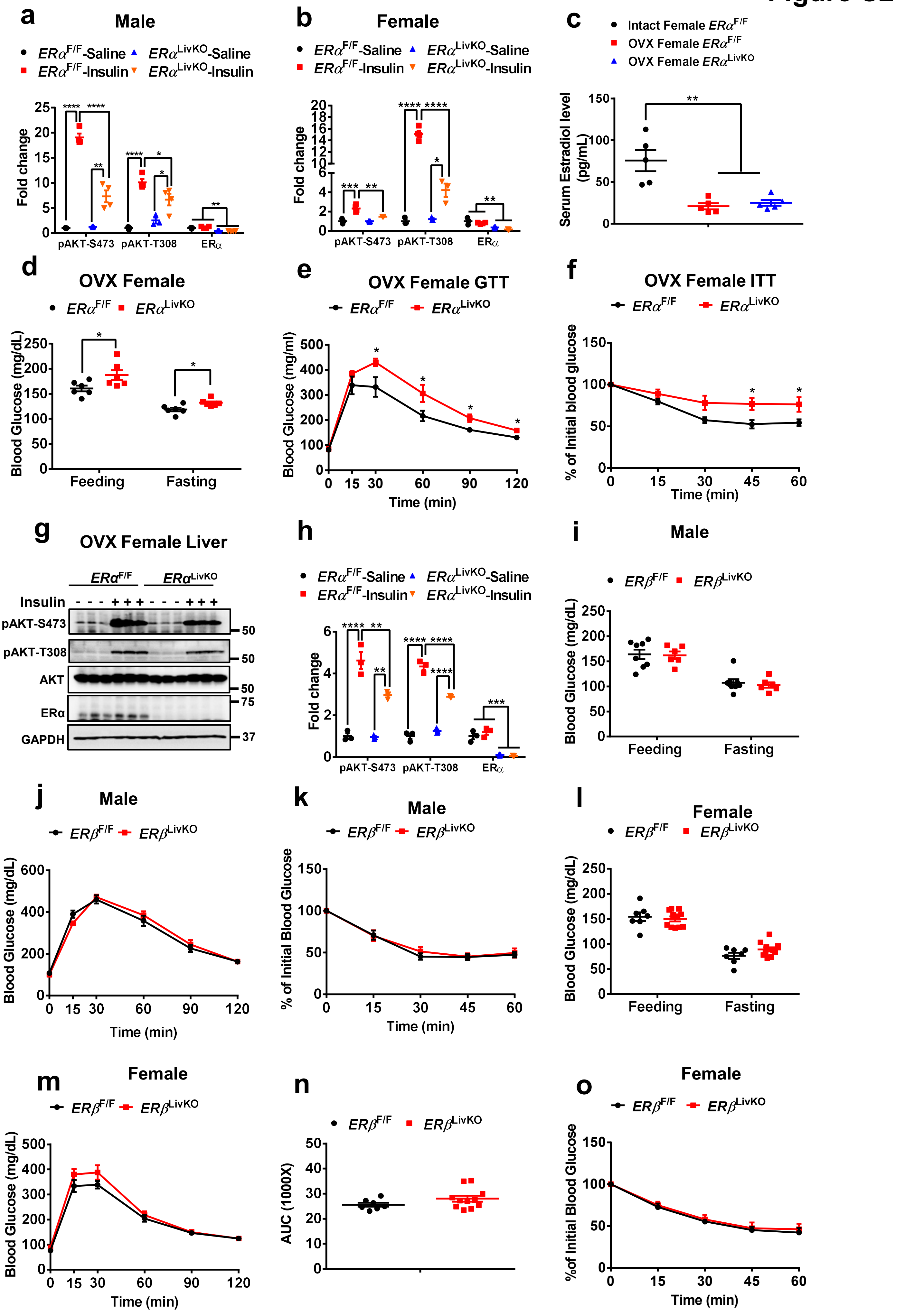
a
Male
ERα F/F-Saline
ERα F/F-Insulin
ERα LivKO-Saline
ERα LivKO-Insulin
Fold change
pAKT-S473
pAKT-T308
ERα
b
Female
c
Intact Female ERα F/F
OVX Female ERα F/F
OVX Female ERα LivKO
Serum Estradiol level (pg/mL)
d
OVX Female
Blood Glucose (mg/dL)
Feeding
Fasting
e
OVX Female GTT
Blood Glucose (mg/ml)
Time (min)
f
OVX Female ITT
% of Initial blood glucose
g
OVX Female Liver
Insulin
pAKT-S473
pAKT-T308
AKT
ERα
GAPDH
50
75
37
h
i
Male
ERβ F/F
ERβ LivKO
j
k
% of Initial Blood Glucose
l
Female
m
n
AUC (1000X)
o
%of Initial Blood Glucose

Fig. S2 Effect of hepatic ERβ on glucose homeostasis in chow diet feeding mice. a Quantification of Figure 1h, $n = 3$ ($ER\alpha^{F/F}$-Saline and $ER\alpha^{LivKO}$-Saline) and 4 ($ER\alpha^{F/F}$-Insulin and $ER\alpha^{LivKO}$-Insulin) mice/group; for pAKT-S473, $ER\alpha^{F/F}$-Saline versus $ER\alpha^{F/F}$-Insulin, $P < 0.0001$, $ER\alpha^{LivKO}$-Saline versus $ER\alpha^{LivKO}$-Insulin, $P = 0.0022$, $ER\alpha^{F/F}$-Insulin versus $ER\alpha^{LivKO}$-Insulin, $P < 0.0001$; for pAKT-T308, $ER\alpha^{F/F}$-Saline versus $ER\alpha^{F/F}$-Insulin, $P < 0.0001$, $ER\alpha^{LivKO}$-Saline versus $ER\alpha^{LivKO}$-Insulin, $P = 0.0230$, $ER\alpha^{F/F}$-Insulin versus $ER\alpha^{LivKO}$-Insulin, $P = 0.0422$; for ERα, $ER\alpha^{F/F}$-Saline versus $ER\alpha^{LivKO}$-Saline, $P = 0.0029$, $ER\alpha^{F/F}$-Insulin versus $ER\alpha^{LivKO}$-Insulin, $P < 0.0001$. **b** Quantification of Figure 1l, $n = 3$ ($ER\alpha^{F/F}$-Saline, $ER\alpha^{LivKO}$-Saline, and $ER\alpha^{LivKO}$-Insulin) and 4 ($ER\alpha^{F/F}$-Insulin) mice/group; for pAKT-S473, $ER\alpha^{F/F}$-Saline versus $ER\alpha^{F/F}$-Insulin, $P = 0.0003$, $ER\alpha^{F/F}$-Insulin versus $ER\alpha^{LivKO}$-Insulin, $P = 0.0058$; for pAKT-T308, $ER\alpha^{F/F}$-Saline versus $ER\alpha^{F/F}$-Insulin, $P < 0.0001$, $ER\alpha^{LivKO}$-Saline versus $ER\alpha^{LivKO}$-Insulin, $P = 0.0102$, $ER\alpha^{F/F}$-Insulin versus $ER\alpha^{LivKO}$-Insulin, $P < 0.0001$; for ERα, $ER\alpha^{F/F}$-Saline versus $ER\alpha^{LivKO}$-Saline, $P = 0.0089$, $ER\alpha^{F/F}$-Insulin versus $ER\alpha^{LivKO}$-Insulin, $P = 0.0058$. **c** Serum estradiol levels in intact and OVX female mice, $n = 5$ mice/group; Intact female $ER\alpha^{F/F}$ versus OVX Female $ER\alpha^{F/F}$, $P = 0.0010$, Intact female $ER\alpha^{F/F}$ versus OVX Female $ER\alpha^{LivKO}$, $P = 0.0019$. **d** Random feeding and 5 h fasting blood glucose in $ER\alpha^{F/F}$ and $ER\alpha^{LivKO}$ OVX female mice, $n = 6$ mice/group; for feeding blood glucose, $P = 0.0428$; for fasting blood glucose, $P = 0.0180$. **e** Glucose tolerance tests in $ER\alpha^{F/F}$ and $ER\alpha^{LivKO}$ OVX female mice, $n = 6$ mice/group; 30 min, $P = 0.0405$, 60 min, $P = 0.0486$, 90 min, $P = 0.0215$, 120 min, $P = 0.0163$. **f** Insulin tolerance tests in $ER\alpha^{F/F}$ and $ER\alpha^{LivKO}$ OVX female mice, $n = 6$ mice/group; 45 min, $P = 0.0209$, 60 min, $P = 0.0475$. **g-h** Insulin signaling was detected in the livers of $ER\alpha^{F/F}$ and $ER\alpha^{LivKO}$ OVX female mice injected with 2 U insulin for 5 min, $n = 3$ mice/group; for pAKT-S473, $ER\alpha^{F/F}$-Saline versus $ER\alpha^{F/F}$-Insulin, $P < 0.0001$, $ER\alpha^{LivKO}$-Saline versus $ER\alpha^{LivKO}$-Insulin, $P = 0.0010$, $ER\alpha^{F/F}$-Insulin versus $ER\alpha^{LivKO}$-Insulin, $P = 0.0035$; for pAKT-T308, $ER\alpha^{F/F}$-Saline versus $ER\alpha^{F/F}$-Insulin, $P < 0.0001$, $ER\alpha^{LivKO}$-Saline versus $ER\alpha^{LivKO}$-Insulin, $P < 0.0001$, $ER\alpha^{F/F}$-Insulin versus $ER\alpha^{LivKO}$-Insulin, $P < 0.0001$; for ERα, $ER\alpha^{F/F}$-Saline versus $ER\alpha^{LivKO}$-Saline, $P = 0.0009$, $ER\alpha^{F/F}$-Insulin versus $ER\alpha^{LivKO}$-Insulin, $P = 0.0002$. **i** Random feeding and 16 h fasting blood glucose in $ER\beta^{F/F}$ and $ER\beta^{LivKO}$ male mice, $n = 6$ ($ER\beta^{LivKO}$) and 8 ($ER\beta^{F/F}$) mice/group. **j** Glucose tolerance tests in $ER\beta^{F/F}$ and $ER\beta^{LivKO}$ male mice, $n = 7$ ($ER\beta^{LivKO}$) and 8 ($ER\beta^{F/F}$) mice/group. **k** Insulin tolerance tests in $ER\beta^{F/F}$ and $ER\beta^{LivKO}$ male mice, $n = 7$ ($ER\beta^{LivKO}$) and 8 ($ER\beta^{F/F}$) mice/group. **l** Random feeding and 16 h fasting blood glucose in $ER\beta^{F/F}$ and $ER\beta^{LivKO}$ female mice, $n = 7$ ($ER\beta^{F/F}$) and 11 ($ER\beta^{LivKO}$) mice/group. **m-n** Glucose tolerance tests in $ER\beta^{F/F}$ and $ER\beta^{LivKO}$ female mice, $n = 7$ ($ER\beta^{F/F}$) and 11 ($ER\beta^{LivKO}$) mice/group. **o** Insulin tolerance tests in $ER\beta^{F/F}$ and $ER\beta^{LivKO}$ female mice, $n = 7$ ($ER\beta^{F/F}$) and 11 ($ER\beta^{LivKO}$) mice/group. Data are presented as mean ± SEM. *$P < 0.05$, **$P < 0.01$, ***$P < 0.001$, ****$P < 0.0001$, unpaired Two-tailed Student's t test (d-f and i-o), One-way ANOVA with Tukey's multiple comparisons test (c), or Two-way ANOVA with Tukey's multiple comparisons test (a, b, and h). Source data are provided as a source data file.

Figure S3

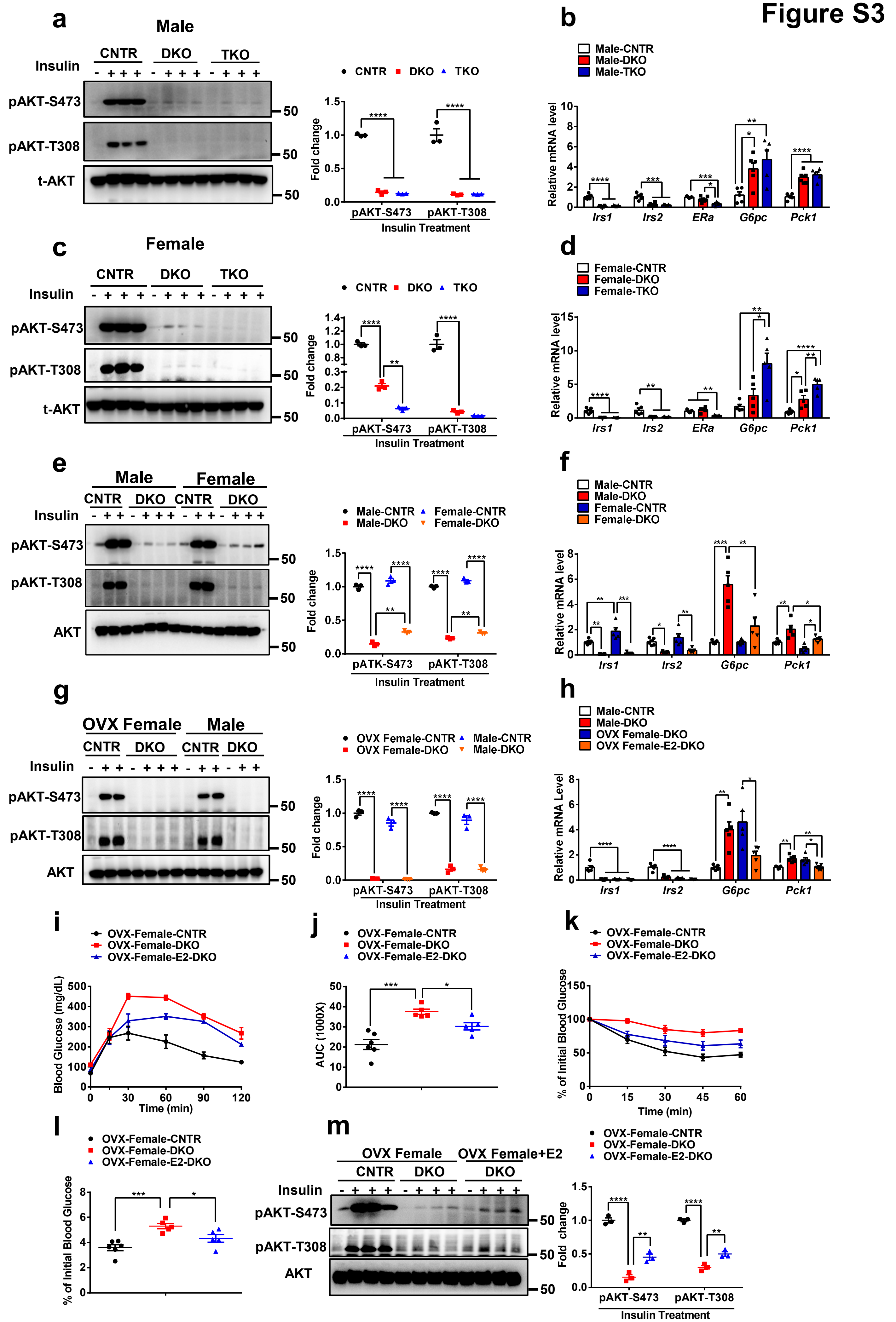

a
Male
CNTR DKO TKO
Insulin
pAKT-S473
pAKT-T308
t-AKT
50
CNTR DKO TKO
Fold change
pAKT-S473 pAKT-T308
Insulin Treatment
b
Male-CNTR
Male-DKO
Male-TKO
Relative mRNA level
Irs1 Irs2 ERa G6pc Pck1
c
Female
CNTR DKO TKO
Insulin
pAKT-S473
pAKT-T308
t-AKT
d
Female-CNTR
Female-DKO
Female-TKO
e
Male Female
CNTR DKO CNTR DKO
AKT
Male-CNTR
Male-DKO
Female-CNTR
Female-DKO
pATK-S473 pAKT-T308
f
g
OVX Female Male
OVX Female-CNTR
OVX Female-DKO
Male-CNTR
Male-DKO
h
OVX Female-DKO
OVX Female-E2-DKO
Relative mRNA Level
i
OVX-Female-CNTR
OVX-Female-DKO
OVX-Female-E2-DKO
Blood Glucose (mg/dL)
Time (min)
j
AUC (1000X)
k
% of Initial Blood Glucose
l
m
OVX Female OVX Female+E2

Fig. S3 Hepatic insulin sensitivity in control, DKO, and TKO male/female mice. a Effect of ERα on hepatic insulin sensitivity in control and DKO male mice injected with 2 U insulin for 5 min, $n = 3$ mice/group; for pAKT-S473 and pAKT-T308, CNTR versus DKO or TKO, $P < 0.0001$. **b** The mRNA expression levels of gluconeogenic genes in the livers of control and DKO male mice under random feeding, $n = 5$ (CNTR and TKO) and 6 (DKO) mice/group; for *Irs1*, CNTR versus DKO or TKO, $P < 0.0001$; for *Irs2*, CNTR versus DKO, $P = 0.0001$, CNTR versus TKO, $P < 0.0001$; for *ERα*, CNTR versus TKO, $P = 0.0007$, DKO versus TKO, $P = 0.0106$; for *G6pc*, CNTR versus DKO, $P = 0.0349$, CNTR versus TKO, $P = 0.0073$; for *Pck1*, CNTR versus DKO, $P < 0.0001$, CNTR versus TKO, $P < 0.0001$. **c** Effect of ERα on hepatic insulin sensitivity in control and DKO female mice injected with 2 U insulin for 5 min, $n = 3$ mice/group; for pAKT-S473, CNTR versus DKO, $P < 0.0001$, DKO versus TKO, $P = 0.0018$; for pAKT-T308, CNTR versus DKO, $P < 0.0001$. **d** The mRNA expression levels of gluconeogenic genes in the livers of control and DKO female mice under random feeding, $n = 5$ mice/group; for *Irs1*, CNTR versus DKO or TKO, $P < 0.0001$; for *Irs2*, CNTR versus DKO, $P = 0.0067$, CNTR versus TKO, $P = 0.0050$; for *ERα*, CNTR versus TKO, $P = 0.0031$, DKO versus TKO, $P = 0.0004$; for *G6pc*, CNTR versus TKO, $P = 0.0035$, DKO versus TKO, $P = 0.0230$; for *Pck1*, CNTR versus DKO, $P = 0.0245$, CNTR versus TKO, $P < 0.0001$, DKO versus TKO, $P = 0.0090$. **e** Hepatic insulin sensitivity in control and DKO male/female mice injected with 2 U insulin for 5 min, $n = 3$ mice/group; for pAKT-S473, Male-CNTR versus Male-DKO, $P < 0.0001$, Female-CNTR versus Female-DKO, $P < 0.0001$, Male-DKO versus Female-DKO, $P = 0.0032$; for pAKT-S473, Male-CNTR versus Male-DKO, $P < 0.0001$, Female-CNTR versus Female-DKO, $P < 0.0001$, Male-DKO versus Female-DKO, $P = 0.0092$. **f** The mRNA expression levels of gluconeogenic genes in the livers of control and DKO male/female mice under random feeding, $n = 5$ mice/group; for *Irs1*, Male-CNTR versus Female-CNTR, $P = 0.0062$, Male-CNTR versus Male-DKO, $P = 0.0022$, Female-CNTR versus Female-DKO, $P < 0.0001$; for *Irs2*, Male-CNTR versus Male-DKO, $P = 0.0135$, Female-CNTR versus Female-DKO, $P < 0.0020$; for *G6pc*, Male-CNTR versus Male-DKO, $P < 0.0001$, Male-DKO versus Female-DKO, $P = 0.0017$; for *Pck1*, Male-CNTR versus Male-DKO, $P = 0.0053$, Female-CNTR versus Female-DKO, $P = 0.0431$, Male-DKO versus Female-DKO, $P = 0.0327$. **g** Hepatic insulin sensitivity in control and DKO male/ OVX female mice injected with 2 U insulin for 5 min, $n = 3$ mice/group; $P < 0.0001$. **h** The mRNA expression levels of gluconeogenic genes in the livers of control, DKO male, DKO OVX female, and DKO OVX-E_2 supplemented mice under random feeding, $n = 5$ mice/group; for *Irs1* and *Irs2*, $P < 0.0001$; for *G6pc*, Male-CNTR versus Male-DKO, $P = 0.0075$, OVX Female-DKO versus OVX Female-E2-DKO, $P = 0.0172$; for *Pck1*, Male-CNTR versus Male-DKO, $P = 0.0052$, OVX Female-DKO versus OVX Female-E2-DKO, $P = 0.0173$, Male-DKO versus OVX Female-E2-DKO, $P = 0.0066$. **i-j** Glucose tolerance tests in OVX control, OVX female, and OVX female-E_2 supplemented mice, $n = 5$ (OVX female and OVX female-E2) and 6 (OVX control) mice/group; OVX-Female-CNTR versus OVX-Female-DKO, $P = 0.0002$, OVX-Female-DKO versus OVX-Female-E2-DKO, $P = 0.0156$. **k-l** Insulin tolerance tests in OVX control, OVX female, and OVX female-E_2 supplemented mice, $n = 5$ (OVX female and OVX female-E2) and 6 (OVX control) mice/group; OVX-Female-CNTR versus OVX-Female-DKO, $P = 0.0009$, OVX-Female-DKO versus OVX-Female-E2-DKO, $P = 0.0492$. **m** Hepatic insulin sensitivity in OVX control, OVX female, and OVX female-E_2 supplemented mice injected with 2 U insulin for 5 min, $n = 3$ mice/group; for pAKT-S473, OVX-Female-CNTR versus OVX-Female-DKO, $P < 0.0001$, OVX-Female-DKO versus OVX-Female-E2-DKO, $P = 0.0049$; for pAKT-T308, OVX-Female-CNTR versus OVX-Female-DKO, $P < 0.0001$, OVX-Female-DKO versus OVX-Female-E2-DKO, $P = 0.0058$. Data are presented as mean ± SEM. *$P < 0.05$, **$P < 0.01$, ***$P < 0.001$, ****$P < 0.0001$, One-way ANOVA with Tukey's multiple comparisons test (a-d and i-m), Two-way ANOVA with Tukey's multiple comparisons test (e-h). CNTR: Control. Source data are provided as a source data file.

Figure S4

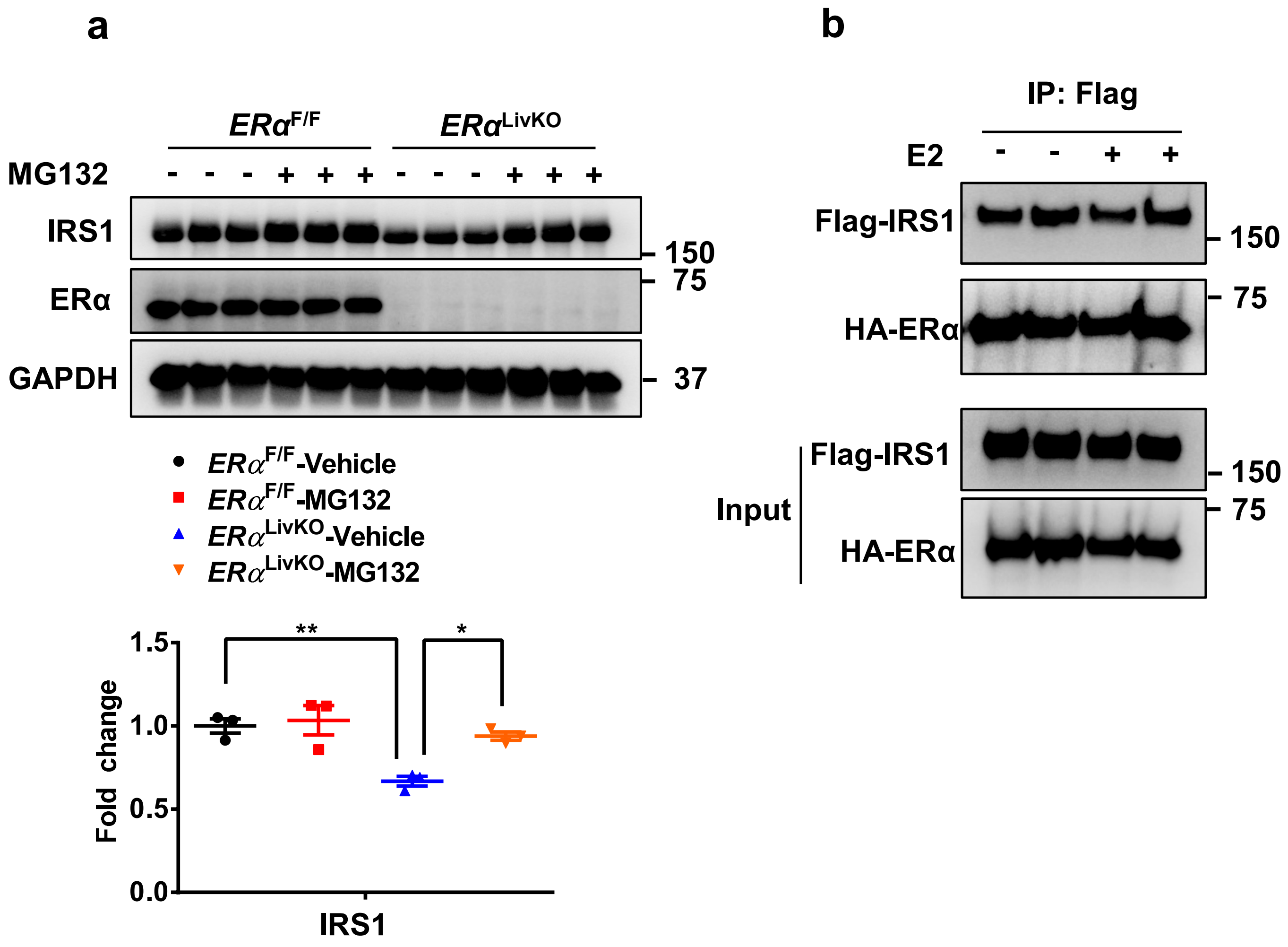

Fig. S4 ERα interacts with IRS1 and regulates IRS1 protein stability. a IRS1 protein levels in control and ERα deficient mouse primary hepatocytes treated with 10 μM MG132 for 3 h, $n = 3$ independent cells; $ER\alpha^{F/F}$ -Vehicle versus $ER\alpha^{LivKO}$ -Vehicle, $P = 0.0092$, $ER\alpha^{LivKO}$ -Vehicle versus $ER\alpha^{LivKO}$ -MG132, $P = 0.0272$. **b** Interaction between ERα and IRS1 in HEK293 cells treated with 100 nM E_2 for 1 h. HA-ERα and Flag-IRS1 plasmids were transfected into HEK293 cells for 30 h, followed by 100 nM E_2 treatment for 1 h. Co-immunoprecipitation was performed. The experiments were repeated independently twice. Data are presented as mean ± SEM. $^*P < 0.05$, $^{**}P < 0.01$, Two-way ANOVA with Tukey's multiple comparisons test. Source data are provided as a source data file.

Figure S5

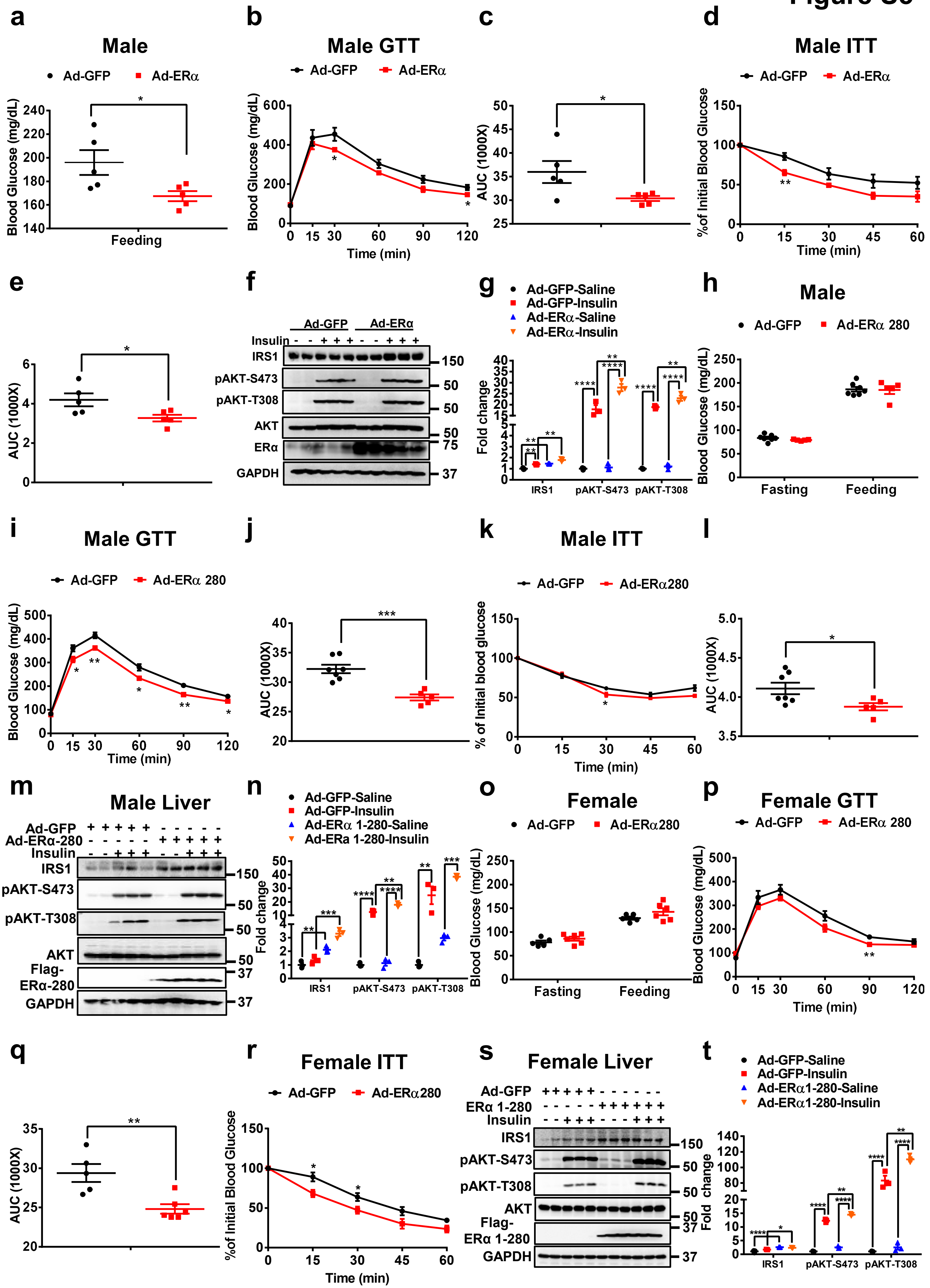

a
Male
Ad-GFP
Ad-ERα
Blood Glucose (mg/dL)
Feeding
b
Male GTT
Ad-GFP
Ad-ERα
Blood Glucose (mg/dL)
Time (min)
c
AUC (1000X)
d
Male ITT
Ad-GFP
Ad-ERα
%of Initial Blood Glucose
Time (min)
e
AUC (1000X)
f
Ad-GFP
Ad-ERα
Insulin
IRS1
pAKT-S473
pAKT-T308
AKT
ERα
GAPDH
g
Ad-GFP-Saline
Ad-GFP-Insulin
Ad-ERα-Saline
Ad-ERα-Insulin
Fold change
IRS1
pAKT-S473
pAKT-T308
h
Male
Ad-GFP
Ad-ERα 280
Blood Glucose (mg/dL)
Fasting
Feeding
i
Male GTT
Ad-GFP
Ad-ERα 280
Blood Glucose (mg/dL)
Time (min)
j
AUC (1000X)
k
Male ITT
Ad-GFP
Ad-ERα280
% of Initial blood glucose
Time (min)
l
AUC (1000X)
m
Male Liver
Ad-GFP
Ad-ERα-280
Insulin
IRS1
pAKT-S473
pAKT-T308
AKT
Flag-ERα-280
GAPDH
n
Ad-GFP-Saline
Ad-GFP-Insulin
Ad-ERα 1-280-Saline
Ad-ERa 1-280-Insulin
Fold change
IRS1
pAKT-S473
pAKT-T308
o
Female
Ad-GFP
Ad-ERα280
Blood Glucose (mg/dL)
Fasting
Feeding
p
Female GTT
Ad-GFP
Ad-ERα 280
Blood Glucose (mg/dL)
Time (min)
q
AUC (1000X)
r
Female ITT
Ad-GFP
Ad-ERα280
%of Initial Blood Glucose
Time (min)
s
Female Liver
Ad-GFP
ERα 1-280
Insulin
IRS1
pAKT-S473
pAKT-T308
AKT
Flag-ERα 1-280
GAPDH
t
Ad-GFP-Saline
Ad-GFP-Insulin
Ad-ERα1-280-Saline
Ad-ERα1-280-Insulin
Fold change
IRS1
pAKT-S473
pAKT-T308

Fig. S5 ERα and ERα 1-280 improves glucose tolerance and insulin sensitivity in mice. a Random feeding blood glucose in male mice infected with Ad-GFP and Ad-ERα, *n* = 5 mice/group; *P* = 0.0366. **b-c** Glucose tolerance tests in male mice infected with Ad-GFP and Ad-ERα, *n* = 5 mice/group; 30 min, *P* = 0.0485, 120 min, *P* = 0.0399, AUC, *P* = 0.0470. **d-e** Insulin tolerance tests in male mice infected with Ad-GFP and Ad-ERα, *n* = 5 mice/group; 15 min, *P* = 0.0063, AUC, *P* = 0.0369. **f-g** Effect of ERα gain-of-function on hepatic insulin sensitivity in male mice treated with 2 U insulin for 5 min, *n* = 3 mice/group; for IRS1, Ad-GFP-Saline versus Ad-GFP-Insulin, *P* = 0.0025, Ad-GFP-Saline versus Ad-ERα-Saline, *P* = 0.0010, Ad-GFP-Insulin versus Ad-ERα-Insulin, *P* = 0.0023, for pAKT-S473, Ad-GFP-Saline versus Ad-GFP-Insulin, *P* < 0.0001, Ad-ERα-Saline versus Ad-ERα-Insulin, *P* < 0.0001, Ad-GFP-Insulin versus Ad-ERα-Insulin, *P* = 0.0021; for pAKT-T308, Ad-GFP-Saline versus Ad-GFP-Insulin, *P* < 0.0001, Ad-ERα-Saline versus Ad-ERα-Insulin, *P* < 0.0001, Ad-GFP-Insulin versus Ad-ERα-Insulin, *P* = 0.0074. **h** Random feeding and 16 h fasting blood glucose in male mice infected with Ad-GFP and Ad-ERα 1-280, *n* = 5 (Ad-ERα 1-280) and 7 (Ad-GFP) mice/group. **i-j** Glucose tolerance tests in male mice infected with Ad-GFP and Ad-ERα 1-280, *n* = 5 (Ad-ERα 1-280) and 7 (Ad-GFP) mice/group; 15 min, *P* = 0.0323, 30 min, *P* = 0.0061, 60 min, *P* = 0.0249, 90 min, *P* = 0.0012, 120 min, *P* = 0.0325, AUC, *P* = 0.0006. **k-l** Insulin tolerance tests in male mice infected with Ad-GFP and Ad-ERα 1-280, *n* = 5 (Ad-ERα 1-280) and 7 (Ad-GFP) mice/group; 30 min, *P* = 0.0344, AUC, *P* = 0.0375. **m-n** Effect of ERα 1-280 gain-of-function on hepatic insulin sensitivity in male mice treated with 2 U insulin for 5 min, *n* = 3 mice/group; for IRS1, Ad-GFP-Saline versus Ad-ERα 1-280-Saline, *P* = 0.0055, Ad-GFP-Insulin versus Ad-ERα 1-280-Insulin, *P* = 0.0001; for pAKT-S473, Ad-GFP-Saline versus Ad-GFP-Insulin, *P* < 0.0001, Ad-ERα 1-280-Saline versus Ad-ERα 1-280-Insulin, *P* < 0.0001, Ad-GFP-Insulin versus Ad-ERα 1-280-Insulin, *P* = 0.0028; for pAKT-T308, Ad-GFP-Saline versus Ad-GFP-Insulin, *P* = 0.0042, Ad-ERα 1-280-Saline versus Ad-ERα 1-280-Insulin, *P* = 0.0003. **o** Random feeding and 16 h fasting blood glucose in female mice infected with Ad-GFP and Ad-ERα 1-280, *n* = 5 (Ad-GFP) and 6 (Ad-ERα 1-280) mice/group. **p-q** Glucose tolerance tests in female mice infected with Ad-GFP and Ad-ERα 1-280, *n* = 5 (Ad-GFP) and 6 (Ad-ERα 1-280) mice/group; 90 min, *P* = 0.0031, AUC, *P* = 0.0047. **r** Insulin tolerance tests in female mice infected with Ad-GFP and Ad-ERα 1-280, *n* = 5 (Ad-GFP) and 6 (Ad-ERα 1-280) mice/group; 15 min, *P* = 0.0173, 30 min, *P* = 0.0372. **s-t** Effect of ERα 1-280 gain-of-function on hepatic insulin sensitivity in female mice treated with 2 U insulin for 5 min, *n* = 3 mice/group; for IRS1, Ad-GFP-Saline versus Ad-ERα 1-280-Saline, *P* < 0.0001, Ad-GFP-Insulin versus Ad-ERα 1-280-Insulin, *P* = 0.0023; for pAKT-S473, Ad-GFP-Saline versus Ad-GFP-Insulin, *P* < 0.0001, Ad-ERα 1-280-Saline versus Ad-ERα 1-280-Insulin, *P* < 0.0001, Ad-GFP-Insulin versus Ad-ERα 1-280-Insulin, *P* = 0.0012; for pAKT-T308, Ad-GFP-Saline versus Ad-GFP-Insulin, *P*< 0.0001, Ad-ERα 1-280-Saline versus Ad-ERα 1-280-Insulin, *P* < 0.0001, Ad-GFP-Insulin versus Ad-ERα 1-280-Insulin, *P* = 0.0021. Data are presented as mean ± SEM. **P* < 0.05, ***P* < 0.01, ****P* < 0.001, *****P* < 0.0001, unpaired Two-tailed Students' t test (a-e, h-l, and o-r), Two-way ANOVA with Tukey's multiple comparisons test (f-g, m-n, and s-t). Source data are provided as a source data file.

Figure S6

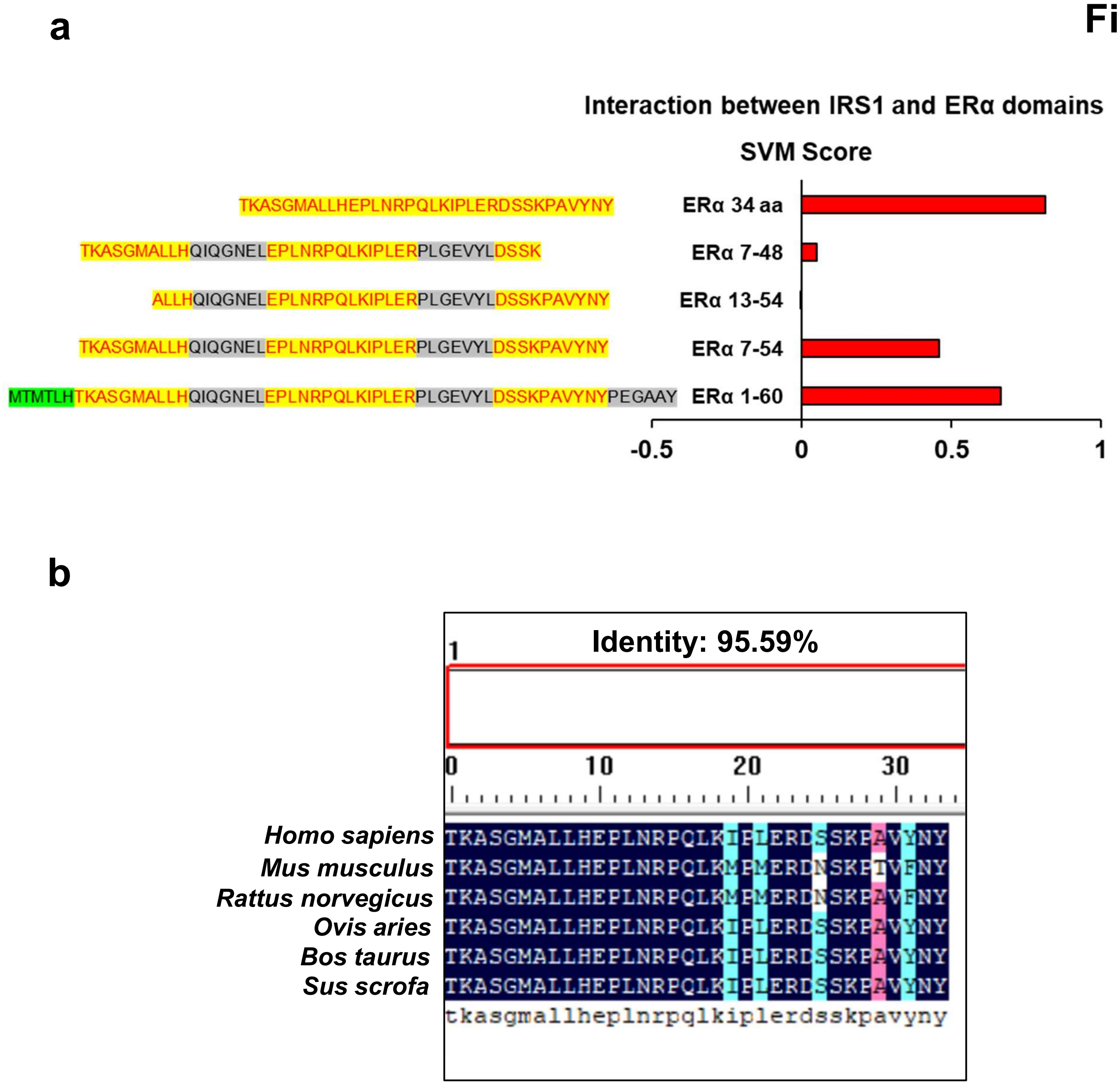

Fig. S6 Optimization of AF1 peptide. a The SVM interaction score between ERα domains and IRS1. **b** AF1 peptide sequence similarity among different species. Source data are provided as a source data file.

Figure S7

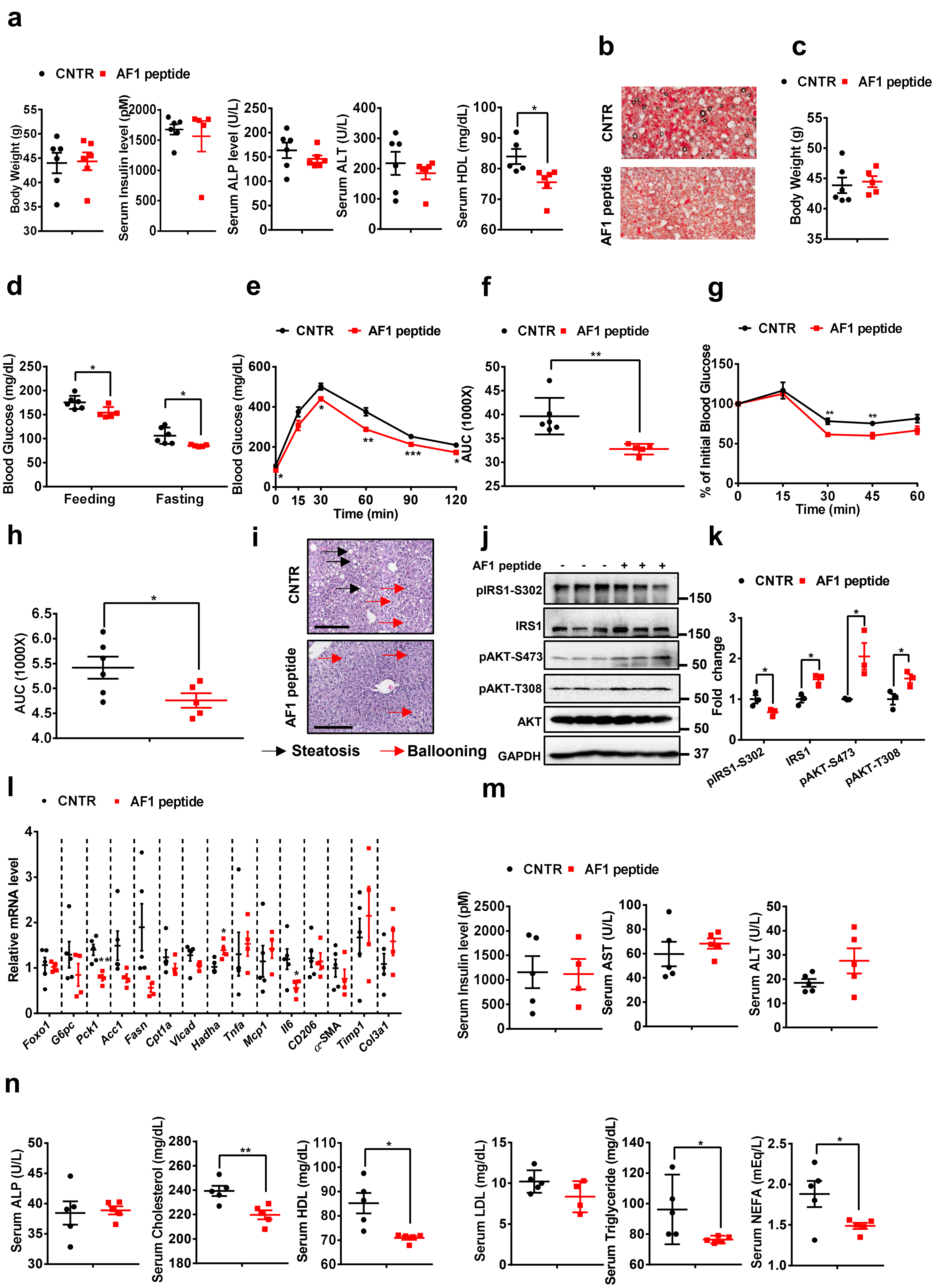

a
CNTR
AF1 peptide
Body Weight (g)
Serum Insulin level (pM)
Serum ALP level (U/L)
Serum ALT (U/L)
Serum HDL (mg/dL)
b
CNTR
AF1 peptide
c
CNTR
AF1 peptide
Body Weight (g)
d
Blood Glucose (mg/dL)
Feeding
Fasting
e
CNTR
AF1 peptide
Blood Glucose (mg/dL)
Time (min)
f
CNTR
AF1 peptide
AUC (1000X)
g
CNTR
AF1 peptide
% of Initial Blood Glucose
Time (min)
h
AUC (1000X)
i
CNTR
AF1 peptide
Steatosis
Ballooning
j
AF1 peptide
pIRS1-S302
IRS1
pAKT-S473
pAKT-T308
AKT
GAPDH
150
150
50
50
50
37
k
CNTR
AF1 peptide
Fold change
pIRS1-S302
IRS1
pAKT-S473
pAKT-T308
l
CNTR
AF1 peptide
Relative mRNA level
Foxo1
G6pc
Pck1
Acc1
Fasn
Cpt1a
Vlcad
Hadha
Tnfa
Mcp1
Il6
CD206
α-SMA
Timp1
Col3a1
m
CNTR
AF1 peptide
Serum Insulin level (pM)
Serum AST (U/L)
Serum ALT (U/L)
n
Serum ALP (U/L)
Serum Cholesterol (mg/dL)
Serum HDL (mg/dL)
Serum LDL (mg/dL)
Serum Triglyceride (mg/dL)
Serum NEFA (mEq/L)

Fig. S7. AF1 peptide improves glucose homeostasis in HFD-induced obesity mice. a Body weight, serum insulin, ALP, ALT, and HDL levels in db/db mice treated with control and AF1 peptide, $n = 5$ (HDL of CNTR group) and 6 mice/group; for serum HDL, $P = 0.0246$. **b** Oil Red O staining in the livers of db/db mice treated with control and AF1 peptide. Representative images were shown. **c** Body weight of HFD-induced obesity mice treated with control and AF1 peptide, $n = 5$ (AF1 peptide) and 6 (control) mice/group. **d** Random feeding and 16 h fasting blood glucose in HFD-induced obesity mice treated with control and AF1 peptide, $n = 5$ (AF1 peptide) and 6 (control) mice/group; feeding blood glucose, $P = 0.0218$, fasting blood glucose, $P = 0.0131$. **e-f** Glucose tolerance tests in HFD-induced obesity mice treated with control and AF1 peptide, $n = 5$ (AF1 peptide) and 6 (control) mice/group; 0 min, $P = 0.0245$, 30 min, $P = 0.0114$, 60 min, $P = 0.0032$, 90 min, $P = 0.0010$, 120 min, $P = 0.0134$, AUC, $P = 0.0039$. **g-h** Insulin tolerance tests in HFD-induced obesity mice treated with control and AF1 peptide, $n = 5$ (AF1 peptide) and 6 (control) mice/group; 30 min, $P = 0.0051$, 45 min, $P = 0.0095$, AUC, $P = 0.0424$. **i** H&E staining of livers from HFD-induced obesity mice treated with control and AF1 peptide. Scale: 200 µm. Representative images were shown. **j-k** Effect of AF1 peptide on hepatic insulin sensitivity in HFD-induced obesity mice, $n = 3$ mice/group; for pIRS1-S302, $P = 0.0413$; for IRS1, $P = 0.0131$; for pAKT-S473, $P = 0.0323$; for pAKT-T308, $P = 0.0413$. **l** Q-PCR analysis of gene expression in livers from HFD-induced obesity mice treated with control and AF1 peptide, $n = 4$ (AF1 peptide) and 5 (control) mice/group; for *Pck1*, $P = 0.0041$; for *Hadha*, $P = 0.0149$; for *Il6*, $P = 0.0499$. **m-n** Serum insulin, ALP, ALT, AST, cholesterol, HDL, LDL, triglyceride, and NEFA levels in HFD-induced obesity mice treated with control and AF1 peptide, $n = 4$ (serum insulin and LDL of AF1 peptide group) and 5 mice/group; serum cholesterol, $P = 0.0080$, serum HDL, $P = 0.0103$, serum triglyceride, $P = 0.0435$, serum NEFA, $P = 0.0443$. Data are presented as mean ± SEM. *$P < 0.05$, **$P < 0.01$, ***$P < 0.001$, unpaired Two-tailed Students' t test. CNTR: Control. Source data are provided as a source data file.

Figure S8

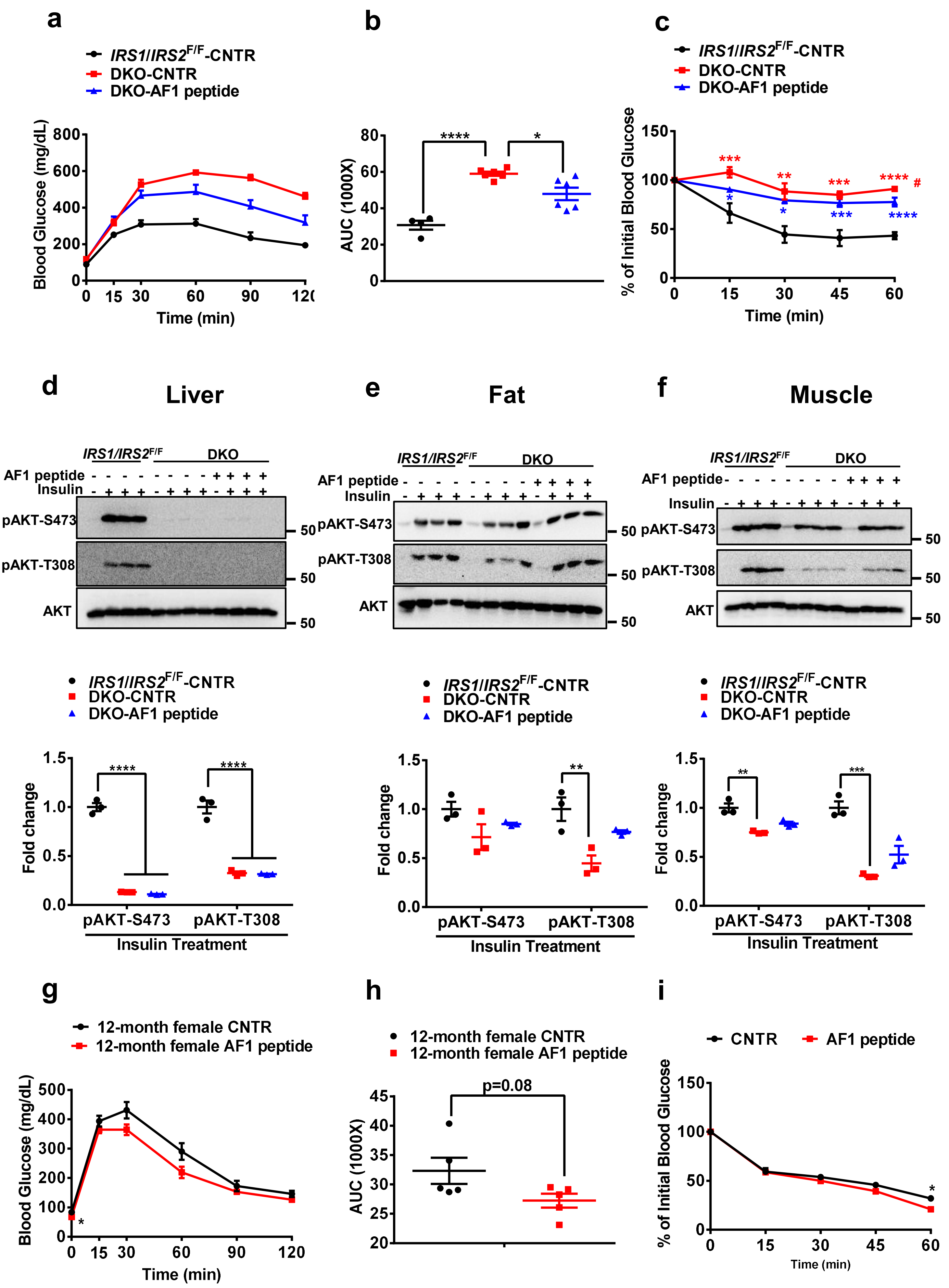

a
IRS1/IRS2F/F-CNTR
DKO-CNTR
DKO-AF1 peptide
Blood Glucose (mg/dL)
Time (min)
b
AUC (1000X)
****
*
c
% of Initial Blood Glucose
#
d
Liver
e
Fat
f
Muscle
IRS1/IRS2F/F
DKO
AF1 peptide
Insulin
pAKT-S473
pAKT-T308
AKT
50
Fold change
Insulin Treatment
g
12-month female CNTR
12-month female AF1 peptide
h
p=0.08
i
CNTR
AF1 peptide

Fig. S8 AF1 peptide improves glucose homeostasis in DKO male and control aging female mice. **a-b** Glucose tolerance tests in DKO male mice treated with control and AF1 peptide, *n* = 4 (CNTR) and 6 (DKO-CNTR and DKO-AF1 peptide) mice/group; *IRS1/IRS2*$^{F/F}$-CNTR versus DKO-CNTR, *P* < 0.0001, DKO-CNTR versus DKO-AF1 peptide, *P* = 0.0179. **c** Insulin tolerance tests in DKO male mice treated with control and AF1 peptide, *n* = 4 (CNTR) and 6 (DKO-CNTR and DKO-AF1 peptide) mice/group. * *IRS1/IRS2*$^{F/F}$-CNTR versus DKO-CNTR, # DKO-CNTR versus DKO-AF1 peptide; 15 min, *IRS1/IRS2*$^{F/F}$-CNTR versus DKO-CNTR, *P* = 0.0005, *IRS1/IRS2*$^{F/F}$-CNTR versus DKO-AF1 peptide, *P* = 0.0285; 30 min, *IRS1/IRS2*$^{F/F}$-CNTR versus DKO-CNTR, *P* = 0.0020, *IRS1/IRS2*$^{F/F}$-CNTR versus DKO-AF1 peptide, *P* = 0.0106; 45 min, *IRS1/IRS2*$^{F/F}$-CNTR versus DKO-CNTR, *P* = 0.0001, *IRS1/IRS2*$^{F/F}$-CNTR versus DKO-AF1 peptide, *P* = 0.0008; 60 min, *IRS1/IRS2*$^{F/F}$-CNTR versus DKO-CNTR, *P* < 0.0001, *IRS1/IRS2*$^{F/F}$-CNTR versus DKO-AF1 peptide, *P* < 0.0001, DKO-CNTR versus DKO-AF1 peptide, *P* = 0.0454. **d** Effect of AF1 peptide on insulin sensitivity in livers from DKO male mice treated with control and AF1 peptide, *n* = 3 mice/group; for pAKT-S473, *IRS1/IRS2*$^{F/F}$-CNTR versus DKO-CNTR or DKO-AF1 peptide, *P* < 0.0001; for pAKT-T308, *IRS1/IRS2*$^{F/F}$-CNTR versus DKO-CNTR or DKO-AF1 peptide, *P* < 0.0001. **e** Effect of AF1 peptide on insulin sensitivity in eWAT from DKO male mice treated with control and AF1 peptide, *n* = 3 mice/group; for pAKT-T308, *IRS1/IRS2*$^{F/F}$-CNTR versus DKO-CNTR, *P* = 0.0083. **f** Effect of AF1 peptide on insulin sensitivity in skeleton muscle from DKO male mice treated with control and AF1 peptide, *n* = 3 mice/group; for pAKT-S473, *IRS1/IRS2*$^{F/F}$-CNTR versus DKO-CNTR, *P* = 0.0008; for pAKT-T308, *IRS1/IRS2*$^{F/F}$-CNTR versus DKO-CNTR, *P* = 0.0006. **g-h** Glucose tolerance tests in aging female mice (12-month-old) treated with AF1 peptide for 5 weeks, *n* = 5 mice/group; 0 min, *P* = 0.0367. **i** Insulin tolerance tests in aging female mice (12-month-old) treated with AF1 peptide for 5 weeks, *n* = 5 mice/group; 60 min, *P* = 0.0436. Data are presented as mean ± SEM. **P* < 0.05, ***P* < 0.01, ****P* < 0.001, *****P* < 0.0001; #*P* < 0.05, unpaired Two-tailed Students' t test (g-i), One-way ANOVA with Tukey's multiple comparisons test (a-f). CNTR: Control. Source data are provided as a source data file.

Supplementary Table 1. Mouse Primer List

Gene Name	Forward 5'-3'	Reverse 5'-3'
ERα	ctgtccagcagtaacgagaaag	cacagtagcgagtctccttgg
G6pc	cattgtggcttccttggtcc	ggcagtatgggataagactg
Pck1	ccatcggctacatccctaag	gacctggtcctccagata
IRS1	cccgttcggtgccaaatagc	gccactggtgaggtatccacatagc
IRS2	acttcccagggtcccactgctg	ggctttggaggtgccacgatag
Foxo1	agatgagtgccctgggcagc	gatggactccatgtcacagt
TNFα	gagaaagtcaacctcctctctg	gaagactcctcccaggtatatg
Il6	ccagagatacaaagaaatgatgg	actccagaagaccagaggaaat
MCP1	caggtgtcccaaagaagctgtag	gggtcagcacagacctctctct
CD206	tgattacgagcagtggaagc	gttcaccgtaagcccaattt
Cpt1α	gccgatcatggttaacagcaact	agaccttgaagtaacggcctc
Vlcad	ccttggtgttagcgttaccct	ggctacatcggatccactcg
Hadha	cgtatgccatgactgtccca	ccaaatttctgcgattcagcaag
Fasn	gcaaggtgtaacattcccctct	accgagtaatgccattcagt
Acc1	cctccgtcagctcagataca	tttactaggtgcaagccagaca
Col3a1	gttctagaggatggctgtactaaacaca	ttgccttgcgtgtttgatattc
Timp1	ggcatcctcttgttgctatcact	gtcatcttgatctcataacgctc
α-SMA	tgctgacagaggcaccactgaa	cagttgtacgtccagaggcatag
Cyclophilin	actgaatggctggatggcaag	tgcccgcaagtcaaaagaaat
