# Supplementary material for: An estrogen receptor α-derived peptide improves glucose homeostasis during obesity
Source: Nat Commun. 2024 Apr 22;15:3410. doi: 10.1038/s41467-024-47687-6 (PMC11035554; doi:10.1038/s41467-024-47687-6)
Supplement: Supplementary file 3 — Reporting Summary [file 41467_2024_47687_MOESM3_ESM.pdf]

nature portfolio

Corresponding author(s): Shaodong Guo

Last updated by author(s): 2024-3-28

Reporting Summary

Nature Portfolio wishes to improve the reproducibility of the work that we publish. This form provides structure for consistency and transparency in reporting. For further information on Nature Portfolio policies, see our Editorial Policies and the Editorial Policy Checklist.

Statistics

For all statistical analyses, confirm that the following items are present in the figure legend, table legend, main text, or Methods section.

n/a	Confirmed	
☐	☒	The exact sample size (n) for each experimental group/condition, given as a discrete number and unit of measurement
☐	☒	A statement on whether measurements were taken from distinct samples or whether the same sample was measured repeatedly
☐	☒	The statistical test(s) used AND whether they are one- or two-sided<br>*Only common tests should be described solely by name; describe more complex techniques in the Methods section.*
☒	☐	A description of all covariates tested
☐	☒	A description of any assumptions or corrections, such as tests of normality and adjustment for multiple comparisons
☐	☒	A full description of the statistical parameters including central tendency (e.g. means) or other basic estimates (e.g. regression coefficient) AND variation (e.g. standard deviation) or associated estimates of uncertainty (e.g. confidence intervals)
☐	☒	For null hypothesis testing, the test statistic (e.g. F, t, r) with confidence intervals, effect sizes, degrees of freedom and P value noted<br>*Give P values as exact values whenever suitable.*
☒	☐	For Bayesian analysis, information on the choice of priors and Markov chain Monte Carlo settings
☒	☐	For hierarchical and complex designs, identification of the appropriate level for tests and full reporting of outcomes
☐	☒	Estimates of effect sizes (e.g. Cohen's d, Pearson's r), indicating how they were calculated

Our web collection on statistics for biologists contains articles on many of the points above.

Software and code

Policy information about availability of computer code

Data collection	ChemiDoc Imaging System (Bio-Rad)<br>CFX 384 Real-Time Sytem (Bio-Rad)<br>CLARIOstar microplate reader (BMG LABTECH)<br>Confocol microscope (Leica)<br>Aperio CS2 Slidescanner (Leica)<br>MRI (EchoMRI)
Data analysis	Image J 1.53K<br>Image Lab version 6.1.0<br>ImageScope version 12.4.3.5008<br>RStudio 2022.12.0<br>Graphpad Prism 6.01<br>Microsoft Excel version 2402

For manuscripts utilizing custom algorithms or software that are central to the research but not yet described in published literature, software must be made available to editors and reviewers. We strongly encourage code deposition in a community repository (e.g. GitHub). See the Nature Portfolio guidelines for submitting code & software for further information.

Data

Policy information about availability of data

All manuscripts must include a data availability statement. This statement should provide the following information, where applicable:

- Accession codes, unique identifiers, or web links for publicly available datasets
- A description of any restrictions on data availability
- For clinical datasets or third party data, please ensure that the statement adheres to our policy

New generated data and source data have been deposited in Figshare. Publicly available Affymetrix human genome array data from healthy and diabetic individuals were downloaded on the Gene Expression Omnibus under the number GSE15653 [https://www.ncbi.nlm.nih.gov/geo/query/acc.cgi?acc=GSE15653]. The bulk RNA-seq data generated in this study have been deposited in the NCBI database under accession code GSE262841 [https://www.ncbi.nlm.nih.gov/geo/query/acc.cgi?acc=GSE262841]. Source data, uncropped blots, and raw counts for bulk RNA-seq for livers of db/db mice treated with control and AF1 peptide are provided with this paper and under the https://doi.org/10.6084/m9.figshare.25153235. Source data are provided with this paper.

Research involving human participants, their data, or biological material

Policy information about studies with human participants or human data. See also policy information about sex, gender (identity/presentation), and sexual orientation and race, ethnicity and racism.

Reporting on sex and gender	N/A
Reporting on race, ethnicity, or other socially relevant groupings	N/A
Population characteristics	N/A
Recruitment	N/A
Ethics oversight	N/A

Note that full information on the approval of the study protocol must also be provided in the manuscript.

Field-specific reporting

Please select the one below that is the best fit for your research. If you are not sure, read the appropriate sections before making your selection.

☒ Life sciences ☐ Behavioural & social sciences ☐ Ecological, evolutionary & environmental sciences

For a reference copy of the document with all sections, see nature.com/documents/nr-reporting-summary-flat.pdf

Life sciences study design

All studies must disclose on these points even when the disclosure is negative.

Sample size	The sample size for the animal experiments was not calculated. We tried our best to include mice at same age in the experiments. Also, we chose the sample size based on the suggestion from previous studies in this field. We included as many animals as possible in the experiments to achieve high statistical significance.
Data exclusions	No data were excluded
Replication	All in vitro experiments were repeated at least twice and data were represented when replicates were successful.
Randomization	For animal experiments, assignment to groups was based on mouse genotype. For adenovirus and peptide injection, mice were randomly allocated into experimental groups. For in vitro experiments, all samples were randomized to different treatment groups.
Blinding	Investigators were not blinded in conducting animal experiments for analysis of body weight, GTT, ITT, and body composition or in vitro experiments because same investigators performed the experiment and knew the genotype of mice or treatments in advance. Serum profile analysis was blinded during measurement and analysis.

Reporting for specific materials, systems and methods

We require information from authors about some types of materials, experimental systems and methods used in many studies. Here, indicate whether each material, system or method listed is relevant to your study. If you are not sure if a list item applies to your research, read the appropriate section before selecting a response.

Materials & experimental systems

n/a	Involved in the study	
☐	☒	Antibodies
☐	☒	Eukaryotic cell lines
☒	☐	Palaeontology and archaeology
☐	☒	Animals and other organisms
☒	☐	Clinical data
☒	☐	Dual use research of concern
☒	☐	Plants

Methods

n/a	Involved in the study	
☒	☐	ChIP-seq
☒	☐	Flow cytometry
☒	☐	MRI-based neuroimaging

Antibodies

Antibodies used

Anti-IRS1, rabbit monoclonal (Cell signaling technology, Cat# 2390, RRID:AB_10692516, 1:1000)
Anti-IRS2, rabbit polyclonal (Cell signaling technology, Cat# 4502, RRID:AB_2125774, 1:1000)
Anti-pAKT-S473, rabbit polyclonal (Cell signaling technology, Cat# 9271,RRID:AB_329825, 1:1000)
Anti-pAKT-T308, rabbit monoclonal (Cell signaling technology, Cat# 13038, RRID:AB_2629447, 1:1000)
Anti-AKT, rabbit monoclonal (Cell signaling technology, Cat# 4691, RRID:AB_915783, 1:1000)
Anti-ERa, rabbit monoclonal (Cell signaling technology, Cat# 13258, RRID:AB_2632959, 1:1000)
Anti-GAPDH, rabbit monoclonal (Cell signaling technology, Cat# 5174, RRID:AB_10622025, 1:3000)
Anti-pIRS1-S302, rabbit polyclonal (Cell signaling technology, Cat# 2384, RRID:AB_330360, 1:1000)
Anti-pIRS1-S307, rabbit polyclonal (Cell signaling technology, Cat# 2381, RRID:AB_330342, 1:1000)
Anti-pIRS1-S636/639, rabbit polyclonal (Cell signaling technology, Cat# 2388, RRID:AB_330339, 1:1000)
Anti-pIRS1-S1101, rabbit polyclonal (Cell signaling technology, Cat# 2385, RRID:AB_330363, 1:1000)
Anti-p85, rabbit polyclonal (Cell signaling technology, Cat# 4292, RRID:AB_329869, 1:1000)
Anti-Ubiquitin, rabbit polyclonal (Cell signaling technology, Cat# 58395, RRID:AB_3075532, 1:1000)
Anti-HA tag, rabbit monoclonal (Cell signaling technology, Cat# 3724, RRID:AB_1549585, 1:1000)
Anti-HA tag, mouse monoclonal (Cell signaling technology, Cat# 2367, RRID:AB_10691311, 1:1000)
Anti-Flag tag, rabbit monoclonal (Cell signaling technology, Cat# 14793, RRID:AB_257229, 1:1000)
Anti-Flag tag, mouse monoclonal (Cell signaling technology, Cat# 8146, RRID:AB_10950495, 1:1000)
Anti-rabbit IgG HRP-linked, goat (Cell signaling technology, Cat# 7074, RRID:AB_2099233, 1:3000)
Anti-mouse IgG HRP-linked, horse (Cell signaling technology, Cat# 7076, RRID:AB_330924, 1:3000)
Anti-rabbit IgG (Alexa Flour 594 Conjugate), goat (Cell signaling technology, Cat# 8889 RRID:AB_2716249, 1:500)
Anti-mouse IgG (Alexa Flour 488 Conjugate), goat (Cell signaling technology, Cat# 4408, RRID:AB_10694704, 1:500)

Validation

Anti-IRS1 (mouse), https://www.cellsignal.com/products/primary-antibodies/irs-1-59g8-rabbit-mab/2390
Anit-IRS2 (rabbit), https://www.cellsignal.com/products/primary-antibodies/irs-2-antibody/4502
Anti-pAKT-S473 (rabbit), https://www.cellsignal.com/products/primary-antibodies/phospho-akt-ser473-antibody/9271
Anti-pAKT-T308 (rabbit), https://www.cellsignal.com/products/primary-antibodies/phospho-akt-thr308-d25e6-xp-rabbit-mab/13038
Anti-AKT (rabbit), https://www.cellsignal.com/products/primary-antibodies/akt-pan-c67e7-rabbit-mab/4691
Anti-ERa (rabbit), https://www.cellsignal.com/products/primary-antibodies/estrogen-receptor-a-d6r2w-rabbit-mab/13258
Anti-GAPDH (rabbit), https://www.cellsignal.com/products/primary-antibodies/gapdh-d16h11-xp-174-rabbit-mab/5174
Anti-pIRS1-S302 (rabbit), https://www.cellsignal.com/products/primary-antibodies/phospho-irs-1-ser302-antibody/2384
Anti-pIRS1-S307 (rabbti), https://www.cellsignal.com/products/primary-antibodies/phospho-irs-1-ser307-antibody/2381
Anti-pIRS1-S636/639 (rabbit), https://www.cellsignal.com/products/primary-antibodies/phospho-irs-1-ser636-639-antibody/2388
Anti-pIRS1-S1101 (rabbit), https://www.cellsignal.com/products/primary-antibodies/phospho-irs-1-ser1101-antibody/2385
Anti-p85 (rabbit), https://www.cellsignal.com/products/primary-antibodies/pi3-kinase-p85-antibody/4292
Anti-Ubiquitin (rabbit), https://www.cellsignal.com/products/primary-antibodies/ubiquitin-p37-antibody/58395
Anti-HA tag (rabbit), https://www.cellsignal.com/products/primary-antibodies/ha-tag-c29f4-rabbit-mab/3724
Anti-HA tag (mouse), https://www.cellsignal.com/products/primary-antibodies/ha-tag-6e2-mouse-mab/2367
Anti-Flag tag (rabbit), https://www.cellsignal.com/products/primary-antibodies/dykddddk-tag-d6w5b-rabbit-mab-binds-to-same-epitope-as-sigma-aldrich-anti-flag-m2-antibody/14793
Anti-Flag tag (mouse), https://www.cellsignal.com/products/primary-antibodies/dykddddk-tag-9a3-mouse-mab-binds-to-same-epitope-as-sigma-aldrich-anti-flag-m2-antibody/8146
Anti-rabbit IgG HRP-linked (goat), https://www.cellsignal.com/products/secondary-antibodies/anti-rabbit-igg-hrp-linked-antibody/7074
Anti-mouse IgG HRP-linked (horse), https://www.cellsignal.com/products/secondary-antibodies/anti-mouse-igg-hrp-linked-antibody/7076
Anti-rabbit IgG (Alexa Flour 594 Conjugate) (goat), https://www.cellsignal.com/products/secondary-antibodies/anti-rabbit-igg-h-l-f-ab-2-fragment-alexa-fluor-594-conjugate/8889
Anti-mouse IgG (Alexa Flour 488 Conjugate) (goat), https://www.cellsignal.com/products/secondary-antibodies/anti-mouse-igg-h-l-f-ab-2-fragment-alexa-fluor-174-488-conjugate/4408

Eukaryotic cell lines

Policy information about cell lines and Sex and Gender in Research

Cell line source(s)	HEK 293 cell line (Cat# ab259776, Abcam) and HepG2 cell line (Cat# 85011430, Sigma)
Authentication	HEK293 and HepG2 cell lines were from commercial source and thus no further authentication was performed before use
Mycoplasma contamination	Cells were not contaminated with mycoplasma
Commonly misidentified lines (See ICLAC register)	No commonly misidentified cells were used in this study

Animals and other research organisms

Policy information about studies involving animals; ARRIVE guidelines recommended for reporting animal research, and Sex and Gender in Research

Laboratory animals	All mice were on C57B/6J background. Mice are housed under controlled environmental conditions, with a temperature of 22-24 C°, humidity maintained at 55% ± 5%, and a 12 h light/12 h dark cycle .<br>Liver-specific ERα (ERαLivKO) or ERβ (ERβLivKO) knockout mice were generated by crossing ERα or ERβ flox mice (gift from Dr. Yong Xu, Baylor College of Medicine) with Albumin-Cre mice purchased from The Jackson Laboratory (Strain #003574), respectively.<br>Liver IRS1 and IRS2 double knockout (DKO) mice were generated by breeding IRS1 and IRS2 flox (IRS1L/L::IRS2 L/L) mice with Albumin-Cre mice. Liver IRS1, IRS2, and ERα triple knockout (TKO) mice were generated by crossing DKO mice with ERαLivKO mice.<br>Db/db mice were purchased from The Jackson Laboratory (Strain #000697).
Wild animals	No wild animals were used in this study.
Reporting on sex	Both sex were used in this study
Field-collected samples	The study did not include field-collected samples.
Ethics oversight	All animal experiments were performed following procedures approved by the Texas A&M University Institutional Animal Care and Use Committee.

Note that full information on the approval of the study protocol must also be provided in the manuscript.
