# Supplementary material for: An estrogen receptor α-derived peptide improves glucose homeostasis during obesity
Source: Nat Commun. 2024 Apr 22;15:3410. doi: 10.1038/s41467-024-47687-6 (PMC11035554; doi:10.1038/s41467-024-47687-6)
Supplement: Supplementary file 2 — Peer Review File [file 41467_2024_47687_MOESM2_ESM.pdf]

Peer Review File

An estrogen receptor α-derived peptide improves glucose homeostasis during obesity

Open Access This file is licensed under a Creative Commons Attribution 4.0 International License, which permits use, sharing, adaptation, distribution and reproduction in any medium or format, as long as you give appropriate credit to the original author(s) and the source, provide a link to the Creative Commons license, and indicate if changes were made. In the cases where the authors are anonymous, such as is the case for the reports of anonymous peer reviewers, author attribution should be to 'Anonymous Referee' followed by a clear attribution to the source work. The images or other third party material in this file are included in the article's Creative Commons license, unless indicated otherwise in a credit line to the material. If material is not included in the article's Creative Commons license and your intended use is not permitted by statutory regulation or exceeds the permitted use, you will need to obtain permission directly from the copyright holder. To view a copy of this license, visit http://creativecommons.org/licenses/by/4.0/.

REVIEWER COMMENTS

Reviewer #1 (Remarks to the Author):

The manuscript by Yang and colleagues addresses the important question of understanding at the molecular level how the estrogen receptor ERα enhances insulin sensitivity in the liver. This work studies in depth how ERα in the absence of added ligands (ligand-independent) promotes insulin sensitivity in the liver by interacting the ERα 1-280 domain with IRS-1, decreasing its ubiquitination and subsequent degradation. The authors used three appropriate mouse models, as well as male, female and ovariectomized females, and conclude that there are two initiated extranuclear pathways, one ligand-dependent, E2-ERα-PI3K-AKT and one ligand-independent ERα-IRS-PI3K-AKT. In addition, they synthesize a peptide to mimic the actions of the ERα 1-280 domain and propose it as a potential therapeutic agent.

These results represent an advancement of knowledge in our mechanistic understanding of how estrogen receptors regulate glucose homeostasis.

Although this paper is already packed with detail, I have some suggestions that may improve the manuscript.

1. To better demonstrate the existence of an E2-ERα-PI3K-AKT pathway, which is only approached by the differences among male, female and ovariectomized, the authors should analyze the effect of E2 treatment in DKO ovariectomized females.

2. I understand this mechanism to involve constitutive control of insulin sensitivity by extranuclear ERα. Can translocation of ERα to the nucleus affect insulin sensitivity? In other words, if E2 is added, does activation of the nuclear pathway decrease the amount of ERα-IRS1 complex by translocation of ERα to the nucleus?

3. line 110. Only in poorly controlled diabetes, if glucose levels are within the appropriate range, there is no effect. Does this imply a dependence on insulin levels?

4. Please homogenize all ITT graphs, some are represented as percentage vs time and other

as [glucose] vs time. Figure 2 E should be Blood glucose (mg/dL).

5. Line 330, it looks like 1-90 have a higher interaction. Please, clarify.

Reviewer #2 (Remarks to the Author):

Yang et al found a ligand independent effect of ERα on the regulation of glucose homeostasis and identified an ERα-derived peptide as a potential insulin sensitizer. Deficiency of ERα but not ERβ in the liver impaired glucose homeostasis in male, female, and ovariectomized (OVX) female mice. Mechanistic studies revealed that ERα promoted hepatic insulin sensitivity by suppressing ubiquitination-induced IRS1 degradation.

1. What is the effect of ERα deficiency in insulin sensitive tissues in adipose tissue and skeletal muscle, in addition to the liver, especially in adipose tissue? Many studies have confirmed that ERα and β have different effects on adipose tissue.
2. With the same consideration, whether ERα-derived AF1 peptide which was found by the authors influenced insulin sensitivity of adipose tissue?
3. Guillaume M et al have revealed the specific role of ERα-AF1 activation is sufficient to elicit metabolic protection (2017).
4. In conducting mechanistic studies of hepatic ERα deficiency (DKO and TKO mouse), whether studies after a high-fat diet were performed? (Fig 3)
5. For mechanistic studies, the authors locked in the ubiquitination of IRS. How was ubiquitination found?
6. In addition to the existing results, what about liver morphological changes, such as hepatocyte steatosis and fat deposition?
7. Does ERα-derived AF1 peptide have a role in insulin sensitivity in aged female mice?

Reviewer #3 (Remarks to the Author):

The manuscript "An estrogen receptor α-derived peptide improves glucose homeostasis during obesity" by Yang et al. investigated the ligand-independent effect of ERα on the regulation of glucose homeostasis and identified an ERα-derived peptide as a potential insulin sensitizer. They found that ERα promoted hepatic insulin sensitivity by suppressing

ubiquitination-induced IRS1 degradation. They further designed a peptide based on the ERα 1-280 domain and investigated the role of this peptide in enhancing insulin sensitivity. This study represents an important advance in the therapeutic intervention of T2DM by targeting the ligand-independent effect of ERα. The manuscript is clearly written and the conclusions are largely supported by their experiments. I'd like to recommend it be accepted after the authors address the following concerns.

1. Anti-obesity peptide designs have been extensively studied, and several recent advances in this field should cited and discussed in context. e.g. defensin-derived peptides (PMID: 35803703), ghrelin, etc.
2. In Figure 2, the exact number of mice used in each group should be provided.
3. In Figure 3, the authors should provide more evidence on the gender-specific effect of hepatic ERα on regulating glucose tolerance.
4. What's the protein similarity between IRS1 and IRS2? It's not clear to me why ERα can directly interact with IRS1 but not IRS2. Does IRS2 have a similar binding domain to that in IRS1?
5. The authors need to explain how to optimize their AF1 peptides. For example, how to evaluate the interaction score between the AF1 domain and IRS1. A quantitative assay on different designed peptides may help answer this question.
6. Does this AF1 peptide work in other DIO animal models, say, rat?

Reviewer #1 (Remarks to the Author):

The manuscript by Yang and colleagues addresses the important question of understanding at the molecular level how the estrogen receptor ERα enhances insulin sensitivity in the liver. This work studies in depth how ERα in the absence of added ligands (ligand-independent) promotes insulin sensitivity in the liver by interacting the ERα 1-280 domain with IRS-1, decreasing its ubiquitination and subsequent degradation. The authors used three appropriate mouse models, as well as male, female and ovariectomized females, and conclude that there are two initiated extranuclear pathways, one ligand-dependent, E2-ERα-PI3K-AKT and one ligand-independent ERα-IRS-PI3K-AKT. In addition, they synthesize a peptide to mimic the actions of the ERα 1-280 domain and propose it as a potential therapeutic agent. These results represent an advancement of knowledge in our mechanistic understanding of how estrogen receptors regulate glucose homeostasis.

Although this paper is already packed with detail, I have some suggestions that may improve the manuscript.

1. To better demonstrate the existence of an E2-ERα-PI3K-AKT pathway, which is only approached by the differences among male, female and ovariectomized, the authors should analyze the effect of E2 treatment in DKO ovariectomized females.

Response: We greatly appreciate reviewer’s advice on analyzing the effect of E2 treatment in DKO OVX female mice. In the revised manuscript, we performed E2 implantation in DKO OVX female mice and analyzed glucose tolerance and detected insulin signaling in livers. We found that E2 supplement increased glucose tolerance, improved insulin sensitivity, and activated hepatic insulin signaling in DKO OVX female mice. These results were included in Figure S3i-m.

2. I understand this mechanism to involve constitutive control of insulin sensitivity by extranuclear ERα. Can translocation of ERα to the nucleus affect insulin sensitivity? In other words, if E2 is added, does activation of the nuclear pathway decrease the amount of ERα-IRS1 complex by translocation of ERα to the nucleus?
Response: We appreciate the reviewer’s opinion. It is a good question. A previous study showed that gain-of-function of nuclear ERα rescued ERα deficiency-induced glucose intolerance and insulin resistance in both male and female mice [1]. This result indicates that nuclear ERα plays an important role in the regulation of insulin sensitivity. Considering IR, IRS, PI3K, and AKT also exist in nucleus[2, 3, 4], we hypothesize that translocation of ERα to the nucleus sensitizes nuclear insulin signaling, thereby promoting Foxo1 nuclear exportation and regulating glucose homeostasis.

We detected the effect of E2 on ERα-IRS1 interaction. We found that E2 treatment did not affect the interaction between ERα and IRS1. This result indicates that ERα-IRS1 interaction is independent of E2. We included this result in Fig. S4b of the revised

manuscript. Considering IRS1 exists in both cytoplasm and nucleus[3], it is possible that ERα interacts with IRS1 in both cytosol and nucleus, thereby increasing insulin sensitivity. How E2-ERα signaling affects cytoplasm and nucleus insulin sensitivity needs to be further investigated. We included this point in the Discussion section on page 22 (line 483-491) of the revised manuscript with track changes.

3. line 110. Only in poorly controlled diabetes, if glucose levels are within the appropriate range, there is no effect. Does this imply a dependence on insulin levels?
Response: In the human livers, the mRNA expression levels of ERα were only significantly decreased in the livers of poorly controlled diabetic patients. We further analyzed the correlation between serum insulin levels and *ERα* mRNA expression levels. We found that there is a negative correlation between hepatic *ERα* mRNA expression and serums insulin levels. Compared to well controlled diabetic patients, poorly controlled diabetic patients showed a higher fasting insulin level. We included this result in Fig. 1d and Fig. S1a of the revised manuscript. DKO (liver IRS1 and IRS2 double knockout) male mice showed hyperglycemia and hyperinsulinemia[5]. We found that hepatic *ERα* mRNA expression levels were significantly decreased in the livers of DKO male mice (Figure. 1, below). To further detect the role of insulin on expression levels of *ERα*, we treated primary hepatocytes with high dose of insulin for 12 h, we found that 200 nM insulin treatment significantly decreased mRNA expression of *ERα*. We included this result in Fig. S2b of the revised manuscript. These results indicate that there is a negative association between insulin levels and hepatic *ERα* mRNA expression levels and high dose of insulin potentially decreases *ERα* expression levels. However, the underlying mechanisms by which insulin downregulated expression levels of *ERα* warrant further investigation in the future study. We included this point in the Discussion section on page 23 (line 509-513) of the revised manuscript with track changes.

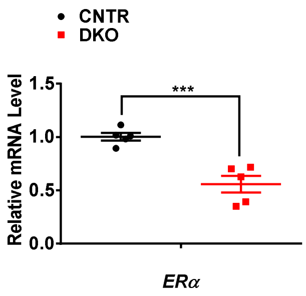

Figure. 1 *ERα* mRNA expression in the livers of control (CNTR) and DKO male mice.

4. Please homogenize all ITT graphs, some are represented as percentage vs time and other as [glucose] vs time. Figure 2 E should be Blood glucose (mg/dL).

Response: We apologize for the inconsistence in the ITT graphs. We changed all the ITT graphs into percentage vs time and corrected the Y axis label in Fig. 2e.

5. Line 330, it looks like 1-90 have a higher interaction. Please, clarify.

Response: In the protein interaction prediction, we set “0” as threshold, which gives a higher sensitivity, specificity, and accuracy. If the SVM score is more than “0”, there is interaction. If lower than “0”, there is no interaction. ERα 1-90 has a higher interaction score more than “0”, which indicates ERα 1-90 has a higher ability to interact with IRS1. To further shorten the functional domain as an insulin sensitizer, we further analyzed ERα 1-90 domain. We found that ERα 1-60 showed a positive score and ERα 60-90 domain showed a negative score. These results indicate that ERα 1-60 is a key domain interacting with IRS1. We clarified this point in the revised manuscript with track changes on page 15 (line 329-335).

Reviewer #2 (Remarks to the Author):

Yang et al found a ligand independent effect of ERα on the regulation of glucose homeostasis and identified an ERα-derived peptide as a potential insulin sensitizer. Deficiency of ERα but not ERβ in the liver impaired glucose homeostasis in male, female, and ovariectomized (OVX) female mice. Mechanistic studies revealed that ERα promoted hepatic insulin sensitivity by suppressing ubiquitination-induced IRS1 degradation.

1. What is the effect of ERα deficiency in insulin sensitive tissues in adipose tissue and skeletal muscle, in addition to the liver, especially in adipose tissue? Many studies have confirmed that ERα and β have different effects on adipose tissue.

Response: ERα also has an important effect on glucose homeostasis in skeletal muscle and adipose tissue. In skeletal muscle, global ERα knockout significantly decreased insulin-mediated glucose uptake in female mice [6]. ERα selective agonist increases membrane GLUT4 localization and silencing of ERα decreases membrane GLUT4 abundance in L6 myoblast [7]. Ablation of skeletal muscle ERα impairs glucose tolerance in the female mice under HFD treatment [8].

In adipose tissue, global ERα deletion leads to insulin resistance and increases adiposity in both male and female mice, with a significant accumulation of visceral fat [9]. The adipose tissue-specific ERα knockout mice provide direct evidence of the effect of ERα on adipose tissue function. Adipose tissue ERα deletion results in enlarged adipocytes as well as increased adipose tissue inflammation and fibrosis in both males and females. Glucose tolerance and insulin sensitivity are largely impaired in the adipose tissue ERα knockout male mice. Body weight gain is largely increased in the adipose tissue ERα knockout female mice [10]. In contrast to ERα, ERβ deletion has no significant effect on visceral fat deposition in male mice under regular diet [11]. However, previous study showed that in the absence of adipocyte ERα, ERβ plays a protective role in suppressing inflammation and fibrosis in the adipose tissues[10]; this result indicates that ERα and ERβ may have a redundant role in regulating the homeostasis of adipose tissues.

We included the effect of ERα on adipose tissue and skeletal muscle in the Discussion section on page 25 (line 558-562) of the revised manuscript with track changes.

2. With the same consideration, whether ERα-derived AF1 peptide which was found by the authors influenced insulin sensitivity of adipose tissue?

Response: In db/db mouse model, we found that AF1 peptide treatment significantly increased insulin sensitivity in adipose tissue, as indicated by significant increases in pAKT-S473 and pAKT-T308 (Fig. 7g in the revised manuscript). Furthermore, we found AF1 peptide signal in the db/db mouse adipose tissue (Figure. 2 below). Although we cannot rule out the possibility that AF1 peptide increases insulin sensitivity in adipose

tissue in an indirect manner by targeting liver, AF1 peptide found in adipose tissue may directly increase adipose tissue insulin sensitivity. In DKO (liver IRS1 and IRS2 double knockout) mice, AF1 peptide improved glucose tolerance and insulin sensitivity. Importantly, we also found that AF1 peptide treatment increased pAKT-473 and pAKT-T308 in adipose tissue but had no effect on hepatic insulin signaling (Fig. S8d, e in the revised manuscript). These results indicate that AF1 peptide could directly target adipose tissue, increase adipose tissue insulin sensitivity, and improve glucose homeostasis. We included this point in the Discussion section on page 25 (line 563-570) in the revised manuscript with track changes.

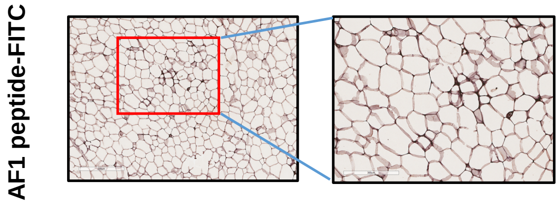

Figure. 2 AF1 peptide shows signal in eWAT. The signal of FITC was detected in eWAT of db/db mice using IHC.

3. Guillaume M et al have revealed the specific role of ERα-AF1 activation is sufficient to elicit metabolic protection (2017).

Response: We acknowledged this finding in the manuscript. In Guillaume et al's study, they focused on the ligand-dependent effect of ERα-AF1. In our study, we identified a ligand-independent effect of ERα and found that ERα-AF1 domain plays a key role in mediating ERα ligand-independent effect. We included this point in the discussion section of the revised manuscript with track changes on page 23 (line 519-523).

4. In conducting mechanistic studies of hepatic ERα deficiency (DKO and TKO mouse), whether studies after a high-fat diet were performed? (Fig 3)

Response: The studies of DKO and TKO mice were performed under regular chow diet. We clarified this point in the legend of Fig. 3 of the revised manuscript.

5. For mechanistic studies, the authors locked in the ubiquitination of IRS. How was ubiquitination found?

Response: We found that mRNA expression levels of IRS1 were not changed by ERα deletion (Fig. 4e), whereas IRS1 proteins levels were significantly decreased in ERα

deficient primary hepatocytes (Fig. 4a). This result indicates that ERα regulates IRS1 protein expression potentially through posttranslational modification. To detect whether ubiquitin-proteasome system mediates the effect of ERα deficiency on IRS1 protein levels, we treated primary hepatocytes with MG132, a proteasome inhibitor. We found that MG132 treatment rescued ERα deficiency-mediated downregulation of IRS1 protein levels. We included this result in Fig. S4a of the revised manuscript. Therefore, we focus on the regulation of ERα in ubiquitination of IRS1. We clarified this point in the Result section on page 12 (line 266-271) of the revised manuscript with track changes.

6. In addition to the existing results, what about liver morphological changes, such as hepatocyte steatosis and fat deposition?

Response: We further analyzed the H&E staining in the livers of db/db mice treated with AF1 peptide. Both CNTR and AF1 peptide-treated db/db mice showed hepatocellular ballooning in livers. AF1 peptide treatment decreased hepatic steatosis structure (Fig. 7e in the revised manuscript). We quantified the liver fat percentage and found that AF1 peptide significantly decreased fat accumulation in the liver of db/db mice. These results are included in Fig. 7e. In addition, we also performed Oil Red O staining to detect liver steatosis. We found that AF1 peptide decreased liver steatosis in db/db mice. We included this result in Fig. S7b.

7. Does ERα-derived AF1 peptide have a role in insulin sensitivity in aged female mice?
Response: We treated 12-month-old female mice with 5 mg/kg body weight AF1 peptide for 5 weeks via intravenous injection (twice per week). The results showed that AF1 peptide led to an increasing trend in glucose tolerance and modestly increased insulin sensitivity in 12-month-old female mice. These results indicate that AF1 peptide potentially improves glucose homeostasis and increases insulin sensitivity in aging female mice. However, the effect of AF1 peptide in aged female mice (18-month-old) needs to be further detected in future study. We included these results in Fig. S8g-h. We discussed this result on page 25 (line 570-574) of the revised manuscript with track changes.

Reviewer #3 (Remarks to the Author):

The manuscript "An estrogen receptor α-derived peptide improves glucose homeostasis during obesity" by Yang et al. investigated the ligand-independent effect of ERα on the regulation of glucose homeostasis and identified an ERα-derived peptide as a potential insulin sensitizer. They found that ERα promoted hepatic insulin sensitivity by suppressing ubiquitination-induced IRS1 degradation. They further designed a peptide based on the ERα 1-280 domain and investigated the role of this peptide in enhancing insulin sensitivity. This study represents an important advance in the therapeutic intervention of T2DM by targeting the ligand-independent effect of ERα. The manuscript is clearly written and the conclusions are largely supported by their experiments. I'd like to recommend it be accepted after the authors address the following concerns.
1. Anti-obesity peptide designs have been extensively studied, and several recent advances in this field should cited and discussed in context. e.g. defensin-derived peptides (PMID: 35803703), ghrelin, etc.

Response: We cited study (PMID:35803703) and discussed the studies about anti-obesity peptide, including glucagon-like peptide-1 (GLP-1), natriuretic peptide, and defensin-derived peptide on page 24 (line 537-540) of the revised manuscript with track changes.

2. In Figure 2, the exact number of mice used in each group should be provided.

Response: We provided the exact number of mice used in the legend of all Figures.

3. In Figure 3, the authors should provide more evidence on the gender-specific effect of hepatic ERα on regulating glucose tolerance.

Response: To detect the gender-specific effect of hepatic ERα on the regulation of glucose tolerance, we detected the expression levels of genes responsible for gluconeogenesis, including *G6pc* and *Pck1*. We found that in male mouse livers, hepatic IRS1 and IRS2 deficiency significantly increased mRNA levels of *G6pc* and *Pck1* compared to control male mice. Hepatic ERα deficiency did not further changed mRNA levels of *G6pc* and *Pck1* in the livers of DKO male mice. In female mouse livers, hepatic IRS1 and IRS2 deficiency led to a nonsignificant increase in mRNA expression levels of *G6pc* and *Pck1* compared to control female mice. Importantly, hepatic ERα deletion significantly increased mRNA expression levels of *G6pc* and *Pck1* in the livers of DKO female mice. Compared to male DKO mice, mRNA expression levels of *G6pc* and *Pck1* were significantly decreased in female DKO mouse livers, which is abolished by ovary removal and rescued by E2 supplementation. We included these results in Fig. S3b, d, f, and h of the revised manuscript.

4. What's the protein similarity between IRS1 and IRS2? It's not clear to me why ERα can directly interact with IRS1 but not IRS2. Does IRS2 have a similar binding domain to that in IRS1?

Response: Our results showed that ERα can interact with both IRS1 and IRS2 (Fig. 4f in the revised manuscript). However, ERα only has a significant effect on IRS1 ubiquitination and its protein expression but not IRS2 (Fig. 4a, g, and h in the revised manuscript). Therefore, we mainly focused on the interaction between ERα and IRS1. IRS1 and IRS2 contain highly similar amino-terminal pleskstrin homology (PH) and phosphotyrosin-binding (PTB) domain. The tail region of IRS1 and IRS2 are poorly conserved. A previous study showed that E3 ubiquitin ligase MG53 promoted IRS1 ubiquitination and degradation but has no effect on IRS2[12]. Therefore, we assume that the distinct effects of ERα on IRS1 and IRS2 protein expression might be attributed to different regulation of E3 ubiquitin ligase in IRS1 and IRS2. We included this point in the Discussion section on page 21 (line 478-484) of the revised manuscript with track changes.

5. The authors need to explain how to optimize their AF1 peptides. For example, how to evaluate the interaction score between the AF1 domain and IRS1. A quantitative assay on different designed peptides may help answer this question.

Response: We appreciate the reviewer's advice. In the revised manuscript, we provided the explanation for the optimization of AF1 peptides. We analyzed the interaction SVM score between truncated ERα 1-60 domain and IRS1. We set "0" as threshold. If SVM score is higher than "0", it is assumed that there exists interaction. A higher SVM score indicates higher potential to interact with IRS1. We found that 1-6 and 55-60 aa of ERα 1-60 domain had no effect on interaction between ERα 1-60 domain and IRS1. However, 7-12 and 49-54 amino acids of ERα 1-60 domain impaired their interaction. Finally, we further shortened the functional domain to 34 aa, which achieves a high SVM score. We included this result in Fig. S6a of the revised manuscript. Please find details in the Result section on page 15 (line 329-344) of the revised manuscript with track changes.

6. Does this AF1 peptide work in other DIO animal models, say, rat?

Response: It is a good question. It is important to further test the effect of AF1 peptide in other DIO animal models. In our lab, we only use mouse model and don't have access to other animal models. We apologize that we cannot perform further experiments to test the effect of AF1 peptide in other animal models, such as rats. However, we compared the sequence similarity of IRS1 and ERα among humans, mice, and rats. We found that both IRS1 and ERα showed a very high similarity (94.45% and 95.39%,

respectively) among human, mouse, and rat (Figure. 3 below). Moreover, AF1 peptide showed a high similarity among different species (95.59%). This result was included in Fig. S6a of the revised manuscript. These results indicate that AF1 peptide potentially plays a role in regulating insulin sensitivity in other DIO animal models, such as rats. However, further experiments in other DIO animal models are warranted. We included this limitation in the Discussion section on age 26 (line 590-592) of the revised manuscript with track changes.

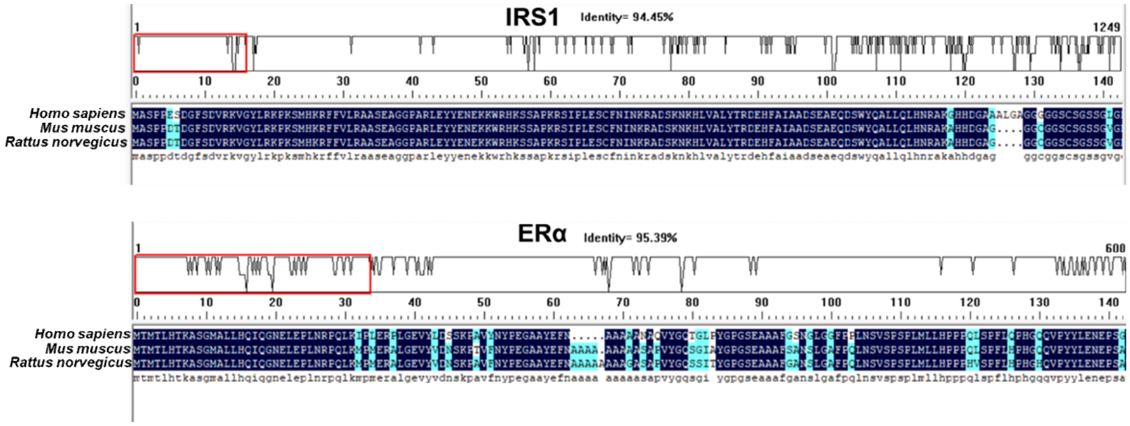

Figure. 3 Amino acid sequence alignment of IRS1 and ERα among humans, mice, and rats.

Reference

1. Allard C*, et al.* Loss of nuclear and membrane estrogen receptor-α differentially impairs insulin secretion and action in male and female mice. *Diabetes* **68**, 490-501 (2019).

2. Hancock ML*, et al.* Insulin receptor associates with promoters genome-wide and regulates gene expression. *Cell* **177**, 722-736. e722 (2019).

3. Prisco M*, et al.* Nuclear translocation of insulin receptor substrate-1 by the simian virus 40 T antigen and the activated type 1 insulin-like growth factor receptor. *Journal of Biological Chemistry* **277**, 32078-32085 (2002).

4. Davis WJ, Lehmann PZ, Li W. Nuclear PI3K signaling in cell growth and tumorigenesis. *Frontiers in cell developmental biology* **3**, 24 (2015).

5. Dong XC*, et al.* Inactivation of hepatic Foxo1 by insulin signaling is required for adaptive nutrient homeostasis and endocrine growth regulation. *Cell metabolism* **8**, 65-76 (2008).

6. Bryzgalova G*, et al.* Evidence that oestrogen receptor-α plays an important role in the regulation of glucose homeostasis in mice: insulin sensitivity in the liver. *Diabetologia* **49**, 588-597 (2006).

7. Galluzzo P, Rastelli C, Bulzomi P, Acconcia F, Pallottini V, Marino M. 17β-Estradiol regulates the first steps of skeletal muscle cell differentiation via ER-α-mediated signals. *American Journal of Physiology-Cell Physiology* **297**, C1249-C1262 (2009).

8. Iñigo MR*, et al.* Estrogen receptor-α in female skeletal muscle is not required for regulation of muscle insulin sensitivity and mitochondrial regulation. *Molecular metabolism* **34**, 1-15 (2020).

9. Heine P, Taylor J, Iwamoto G, Lubahn D, Cooke P. Increased adipose tissue in male and female estrogen receptor-α knockout mice. *Proceedings of the National Academy of Sciences* **97**, 12729-12734 (2000).

10. Davis KE*, et al.* The sexually dimorphic role of adipose and adipocyte estrogen receptors in modulating adipose tissue expansion, inflammation, and fibrosis. *Molecular metabolism* **2**, 227-242 (2013).

11. Ohlsson C*, et al.* Obesity and disturbed lipoprotein profile in estrogen receptor-α-deficient male mice. *Biochemical biophysical research communications* **278**, 640-645 (2000).

12. Song R*, et al.* Central role of E3 ubiquitin ligase MG53 in insulin resistance and metabolic disorders. *Nature* **494**, 375-379 (2013).

REVIEWERS' COMMENTS

Reviewer #1 (Remarks to the Author):

The authors have answered all my points satisfactorily. I recommend this work to be published.

Reviewer #3 (Remarks to the Author):

The authors have properly addressed all my concerns. I'd like to recommend it to be accepted.
